# Rehabilitation at Home Using Mobile Health for Older Adults Hospitalized for Ischemic Heart Disease: The RESILIENT Randomized Clinical Trial

**DOI:** 10.1001/jamanetworkopen.2024.53499

**Published:** 2025-01-02

**Authors:** John A. Dodson, Samrachana Adhikari, Antoinette Schoenthaler, Judith S. Hochman, Greg Sweeney, Barbara George, Kevin Marzo, Lee A. Jennings, Lara C. Kovell, Matthew Vorsanger, Stephanie Pena, Yuchen Meng, Ashwini Varghese, Camila Johanek, Michelle Rojas, Riley McConnell, Jonathan Whiteson, Andrea B. Troxel

**Affiliations:** New York University Langone Medical Center, New York; New York University Langone Medical Center, New York; New York University Langone Medical Center, New York; New York University Langone Medical Center, New York; New York University Langone Medical Center, New York; New York University Langone Medical Center, New York; New York University Langone Medical Center, New York; Geriatrics and Palliative Medicine, College of Medicine, The University of Oklahoma Health Sciences Center, Oklahoma City; Division of Cardiology, Department of Medicine, University of Massachusetts, Worcester; New York University Langone Medical Center, New York; New York University Langone Medical Center, New York; New York University Langone Medical Center, New York; New York University Langone Medical Center, New York; New York University Langone Medical Center, New York; New York University Langone Medical Center, New York; New York University Langone Medical Center, New York; New York University Langone Medical Center, New York; New York University Langone Medical Center, New York

## Abstract

**IMPORTANCE:**

Among older adults with ischemic heart disease, participation in traditional ambulatory cardiac rehabilitation (CR) remains low. While mobile health CR (mHealth-CR) provides a novel opportunity to deliver care, age-specific impairments to technology use may limit uptake, and efficacy data are currently lacking.

**OBJECTIVE:**

To test whether mHealth-CR improves functional capacity in older adults.

**DESIGN, SETTING, AND PARTICIPANTS:**

The RESILIENT phase 2, multicenter, randomized clinical trial recruited patients aged 65 years or older with ischemic heart disease (defined as a hospital visit for myocardial infarction or coronary revascularization) from 5 academic hospitals in New York, Connecticut, and Massachusetts between January 9, 2020, and April 22, 2024.

**INTERVENTION:**

Participants were randomized 3:1 to mHealth-CR or usual care. mHealth-CR consisted of commercially available software delivered on a tablet computer, coupled with remote monitoring and weekly exercise therapist telephone calls, delivered over a 3-month duration. As RESILIENT was a trial conducted in a routine care setting to inform decision-making, participants in both arms were also allowed to receive traditional CR at their cardiologist’s discretion.

**MAIN OUTCOMES AND MEASURES:**

The primary outcome was change from baseline to 3 months in functional capacity, measured by 6-minute walk distance (6MWD). Secondary outcomes were health status (12-Item Short Form Health Survey [SF-12]), residual angina, and impairment in activities of daily living.

**RESULTS:**

A total of 400 participants (median age, 71.0 years [range, 65.0–91.0 years]; 291 [72.8%] male) were randomized to mHealth-CR (n = 298) or usual care (n = 102) and included in the intention-to-treat analysis. Of those, 356 participants (89.0%) returned in person for 6MWD assessment at 3 months. For the primary outcome, there was no adjusted difference in 6MWD between participants receiving mHealth-CR vs usual care (15.6 m; 95% CI, −0.3 to 31.5 m; *P* = .06). Among subgroups, there was an improvement in 6MWD among women (36.6 m; 95% CI, 8.7–64.4 m). There were no differences in any secondary outcomes between groups (eg, adjusted difference in SF-12 physical component scores at 3 months: −1.9 points; 95% CI, −3.9 to 0.2 points). Based on inverse propensity score weighting, there was no effect of mHealth-CR on 6MWD among those who did not attend traditional CR (25.7 m; 95% CI, −8.7 to 60.2 m).

**CONCLUSIONS AND RELEVANCE:**

In this randomized clinical trial of mHealth-CR vs usual care, mHealth-CR did not significantly increase 6MWD or result in improvements in secondary outcomes. The findings suggest the older adult population may require more age-tailored mHealth strategies to effectively improve outcomes.

**TRIAL REGISTRATION:**

ClinicalTrials.gov Identifier: NCT03978130

## Introduction

For patients with ischemic heart disease, participation in traditional ambulatory cardiac rehabilitation (CR) remains low despite decades of evidence about its benefits.^1–3^ Use of CR is even lower among older adults due to barriers that include physical limitations, sensory impairments, lack of transportation, and cost.^4,5^ Therefore, while older adults may have the greatest potential to benefit from CR because of their higher risk of adverse disease-related sequelae, they are also the least likely to participate.^4,6,7^

Mobile health CR (mHealth-CR), which involves delivery of rehabilitation at home via portable electronic devices, has proliferated rapidly in recent years as an alternative approach to traditional ambulatory CR.^5,8,9^ mHealth-CR programs vary but typically include exercise documentation, remote hemodynamic monitoring, video education, and electronic communication with an exercise therapist.^5,8^ While mHealth-CR has the potential to reduce participation barriers, to date it remains largely untested among the older adult population, and whether these individuals experience clinical benefit is therefore unclear.

In this context, we designed the Rehabilitation at Home Using Mobile Health in Older Adults After Hospitalization for Ischemic Heart Disease (RESILIENT) trial. RESILIENT was a prospective, multicenter, nonblinded randomized clinical trial (with blinded assessment of the primary end point). The main objective of RESILIENT was to evaluate whether mHealth-CR improved functional capacity, as measured by 6-minute walk distance (6MWD), compared with usual care among patients aged 65 years or older with ischemic heart disease.

## Methods

### Trial Conduct and Oversight

A full description of the RESILIENT trial (NCT03978130) was published previously.^10^ In brief, the trial was conducted at 5 academic hospitals within 4 health systems: NYU Langone Health–Main Campus (New York, New York), NYU Langone Health–Long Island (Mineola, New York), Bellevue Hospital (New York, New York), University of Massachusetts (Worcester, Massachusetts), and Yale New Haven Health (New Haven, Connecticut). Enrollment occurred between January 9, 2020, and January 10, 2024, with the last follow-up visit on April 22, 2024. NYU Langone Health served as the coordinating center for both study administration and data management. We designed RESILIENT using pragmatic trial principles that included minimal exclusion criteria, use of currently employed exercise therapists, and collection of several patient-reported outcome measures and allowed for clinicians to still refer trial patients to traditional CR if deemed clinically necessary.^11,12^ The full RESILIENT trial protocol is included in Supplement 1. We followed the Consolidated Standards of Reporting Trials (CONSORT) reporting guideline in our data analysis and writing of the manuscript. The study operated through a single institutional review board (IRB) mechanism using the NYU Langone Health IRB, and all study participants provided written informed consent. An independent data safety monitoring board met every 6 months to review enrollment and potential study-related adverse events (eAppendix 1 in Supplement 2).

### Trial Population

Patients were eligible for inclusion in RESILIENT if they were aged 65 years or older and had an acute event related to ischemic heart disease, which was operationalized as a hospital visit for either acute myocardial infarction (AMI) or coronary revascularization (percutaneous coronary intervention [PCI] or coronary artery bypass grafting [CABG]). Exclusion criteria were designed to ensure safety and are listed in the eMethods in Supplement 2.

Participants were identified through daily electronic health record screening of hospital lists with the index condition of AMI, elective PCI, or CABG. After providing informed consent, participants underwent the 6-minute walk test (6MWT) to measure baseline 6MWD. Those who completed the 6MWT were then randomly allocated in a 3:1 manner (using permuted block randomization with variable block sizes of 4 and 8) to intervention (mHealth-CR) or control. One study statistician (S.A.) generated the randomization code, and assignments were administered through REDCap. The rationale behind 3:1 randomization was to provide sufficient power to explore differential patterns of engagement with the mHealth-CR intervention while preserving statistical power for the comparison between mHealth-CR and usual care for future research.

Other measures obtained during the baseline study visit included the 7-Item Seattle Angina Questionnaire (SAQ-7),^13^ 12-Item Short Form Health Survey (SF-12),^14^ activities of daily living (ADLs) and instrumental ADLs (IADLs),^15^ and goal attainment scaling,^16^ all of which were also collected as outcomes at 3 months. In addition, for descriptive purposes, depressive symptoms were measured via the 8-Item Patient Health Questionnaire,^17^ and frailty was measured based on the criteria described by Fried et al.^18^ Race and ethnicity were based on self-report and were collected to describe the diversity of the study sample. Race categories were Asian, Black, White, and multiracial or other (included American Indian or Alaska Native, Native Hawaiian or Other Pacific Islander, or participants self-selecting “other” without further specification); ethnicity categories were Hispanic and non-Hispanic. Other relevant clinical data were obtained via abstraction from the electronic health record. The full schedule of study activities is listed in the eMethods in Supplement 2.

### Study Intervention

Participants assigned to the study intervention received an mHealth-CR program that consisted of 3 components designed to work in concert: (1) mHealth-CR software, (2) counseling by an exercise therapist, and (3) remote physiologic monitoring. The intervention took place for a duration of 3 months, consistent with the duration of traditional ambulatory CR programs. The intervention has been described previously^10^ and is briefly summarized herein.

The mHealth-CR software was a commercially available product developed by Moving Analytics and was purchased for use in the trial (eFigure 1 in Supplement 2). All participants received a tablet computer with software for the duration of the trial. Counseling by the exercise therapist included an initial in-person visit and weekly remote counseling sessions conducted by telephone. Participants were instructed to exercise for at least 5 of 7 days/week, with an ideal goal of 150 minutes/week of moderate-intensity exercise.^19^ Participants rated their exercise intensity with the mHealth-CR software using the Borg Rating of Perceived Exertion (target range, 11–14 on a scale from 6 [no exertion] to 20 [maximum effort]).^20^ During weekly telephone calls, exercise recommendations were titrated based on self-reported exertion and step count data. Encounters were recorded, and periodic auditing was performed by a study investigator with expertise in behavioral interventions (A.S.) to ensure fidelity to the study protocol. Remote physiologic monitoring was obtained with 2 devices provided at baseline: an activity monitor (Fitbit Inspire) and blood pressure cuff (Omron HEM-9200T). Both devices were connected via Bluetooth to the study tablet at the baseline visit, with data viewable through the Moving Analytics platform.

As RESILIENT was designed as a pragmatic trial (ie, conducted in a routine care setting to inform decision-making), participants in both the intervention and control arms were permitted to attend traditional ambulatory CR programs in accordance with current standard of care.^3^ This was left to the discretion of the treating cardiologist.

### End Points

The prespecified primary end point was change in 6MWD, reflective of functional capacity, measured by 6MWT. We chose 6MWD given its reproducibility, correlation with outcomes (mortality, ischemic events),^21–23^ and use by ambulatory CR programs as a measure of effectiveness.^24^ 6MWD was obtained in person at baseline and at 3 months by a clinically credentialed assessor (nurse or exercise therapist) who was blinded to treatment assignment. This assessor was only present for the period during which the 6MWT was performed. The 6MWT was performed in accordance with consensus guidelines.^25^

Secondary end points obtained at 3 months included angina burden (SAQ-7 angina frequency score),^13^ general health status (SF-12),^14^ ADLs and IADLs,^15^ and goal attainment (goal attainment scaling).^26^ We analyzed the proportion of participants with any angina (SAQ-7 score <100 [score range 0–100, with higher scores indicating less frequent angina]) vs no angina at 3 months in a similar manner to prior studies.^27,28^ From SF-12, we calculated the physical component score and mental component score, both of which range from 0 to 100, with higher values indicating better health status. For ADLs and IADLs, we considered a response of “impaired” in any domain to constitute the presence of impairment. Goal attainment, given its complexity in measurement and analysis, will be reported separately.

Safety end points included hospitalization for events during the 3-month intervention period that may plausibly have been related to home exercise: fall-related injury, acute coronary syndrome, or unstable arrhythmia. All serious adverse events were collected via both electronic health record surveillance and participant interviews and classified in accordance with National Institutes of Health guidelines.

### Statistical Analysis

The null hypothesis was that mHealth-CR would not improve functional capacity as measured by change in 6MWD between intervention and control arms at 3 months. We estimated that to reject the null hypothesis and detect a clinically important difference in functional capacity (25 m, based on prior literature^29^), 320 participants (240 intervention, 80 control) with complete primary end points would provide approximately 90% power using a 2-sided test with an α level of 0.05 and a conservative SD estimate of 60 m.^24^ This also provided 80% power to detect a difference as small as 22 m. There were no interim analyses planned before completion of the trial. We enrolled 400 study participants to account for a projected attrition rate of 20% between baseline and 3 months.

All statistical analyses were performed using R, version 4.2.2 (R Project for Statistical Computing). We used covariate-adjusted analysis for primary and secondary end points, adjusting for prespecified prognostic covariates and study site.^30^ For analysis of the primary end point (change in 6MWD), we used a linear regression model. We regressed 3-month 6MWD on a binary indicator of treatment group, with adjustment for baseline 6MWD, the stratification factor (enrollment site), age, and presence of diabetes. These covariates were chosen a priori given their plausible relationship with the study outcome. We proceeded in a similar manner for secondary end points, using linear regression for continuous outcomes and logistic regression for binary outcomes. For the primary end point, we reported point estimates and 95% CIs of the regression coefficient, and we tested for statistical significance using a 2-sided threshold of *P* < .05. For secondary end points, we reported point estimates and corresponding 95% CIs. We did not adjust for multiplicity when conducting tests for secondary outcomes, and these 95% CIs should not be used to draw inferences.

We performed the primary analysis based on the principle of intention to treat. We conducted primary and secondary outcome analyses for all randomized participants (intention-to-treat sample) by imputing outcomes for those who did not complete the trial. We used multiple imputation using chained equations for imputation after assessing summaries of baseline variables among those with and without missingness (further described in eAppendix 2 in Supplement 2). Baseline variables, randomization status, all primary and secondary end points, and a priori identified subgroups were included in the imputation model. We performed 50 imputations and combined results from the imputed datasets using the Rubin rule.^31^

We assessed the primary end point, difference in 6MWD between treatment arms, in clinically relevant prespecified subgroups. We evaluated the primary end point by treatment arm within each stratum of the subgroups using the aforementioned methods.

As a supportive analysis, we used a bayesian framework to assess the posterior predictive distribution of the difference in 6MWD change between the 2 arms and assessed model convergence visually based on trace plots and using the Gelman-Rubin convergence diagnostic.^32^ We also assessed the change in primary outcome after adjusting for traditional CR attendance using inverse propensity score weighting. These analyses are further detailed in eAppendix 2, eFigure 2, and eTable 3 in Supplement 2.

## Results

### Patients

**Figure 1.**
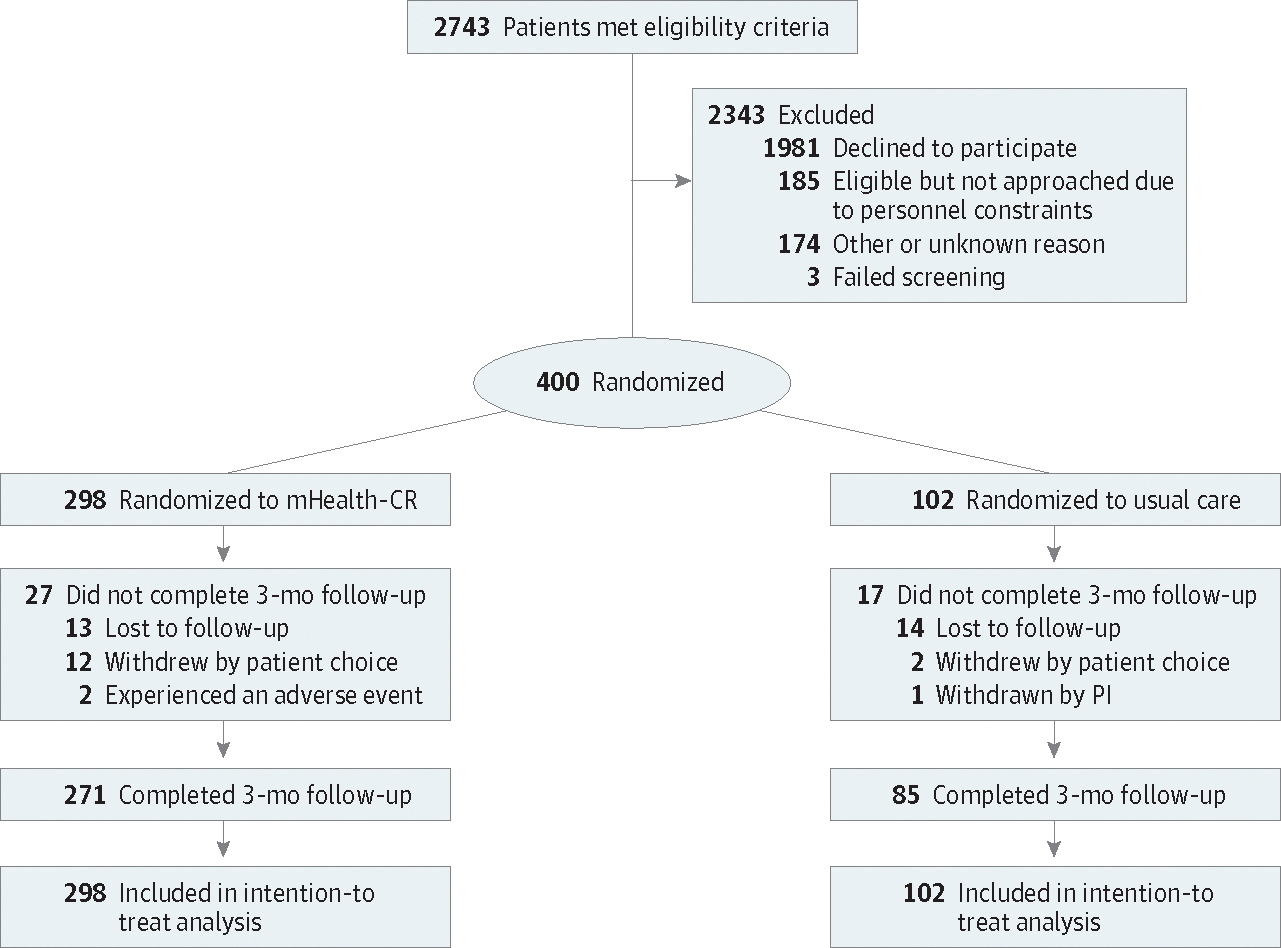
CONSORT Flow Diagram for Randomization and Follow-Up of Study Participants mHealth-CR indicates mobile health–enabled cardiac rehabilitation; PI, principal investigator.

A total of 2743 patients met eligibility criteria, of whom 1981 (72.2%) declined informed consent to participate. Between January 9, 2020, and January 10, 2024, 400 patients were enrolled (median age, 71.0 years [range, 65.0–91.0 years]; 105 [26.2%] aged ≥75 years); 291 (72.8%) were men, and 109 (27.2%) were women. Of these patients, 254 (63.5%) enrolled with the entry criterion of elective PCI. A total of 17 patients (4.2%) were Asian, 36 (9.0%) were Black, 303 (75.8%) were White, and 44 (11.0%) were multiracial or other race; 34 (8.5%) were Hispanic, and 366 (91.5%) were non-Hispanic. Most (370 [92.5%]) had 2 or more chronic medical conditions, and 261 (65.2%) were classified as having either frailty or prefrailty. Additional baseline characteristics are shown in Table 1. There were 298 participants assigned to the mHealth-CR group and 102 assigned to the control group (Figure 1). Median time from discharge to initiation of mHealth-CR was 18 days (IQR, 13–24 days). There were 356 participants who returned in person at 3 months for 6MWD assessment (89.0% of the enrolled study sample; 271 [90.9%] in the mHealth-CR group and 85 [83.3%] in the usual care group). There were no deaths among study participants between baseline and 3 months. Those lost to follow-up at 3 months were more often female (16 [36.4%] vs 93 [26.1%]) and were older (72.5 years [range, 65.0–89.0] years vs 71.0 years [range, 65.0–91.0] years) compared with those retained. Full data on characteristics of those lost to follow-up are provided in eTables 1 and 2 in Supplement 2. Between baseline and 3 months, 38 of 298 participants (12.8%) in the intervention arm and 26 of 102 participants (25.5%) in the usual care arm attended traditional ambulatory CR.

### Efficacy

#### Primary End Point

**Figure 2.**
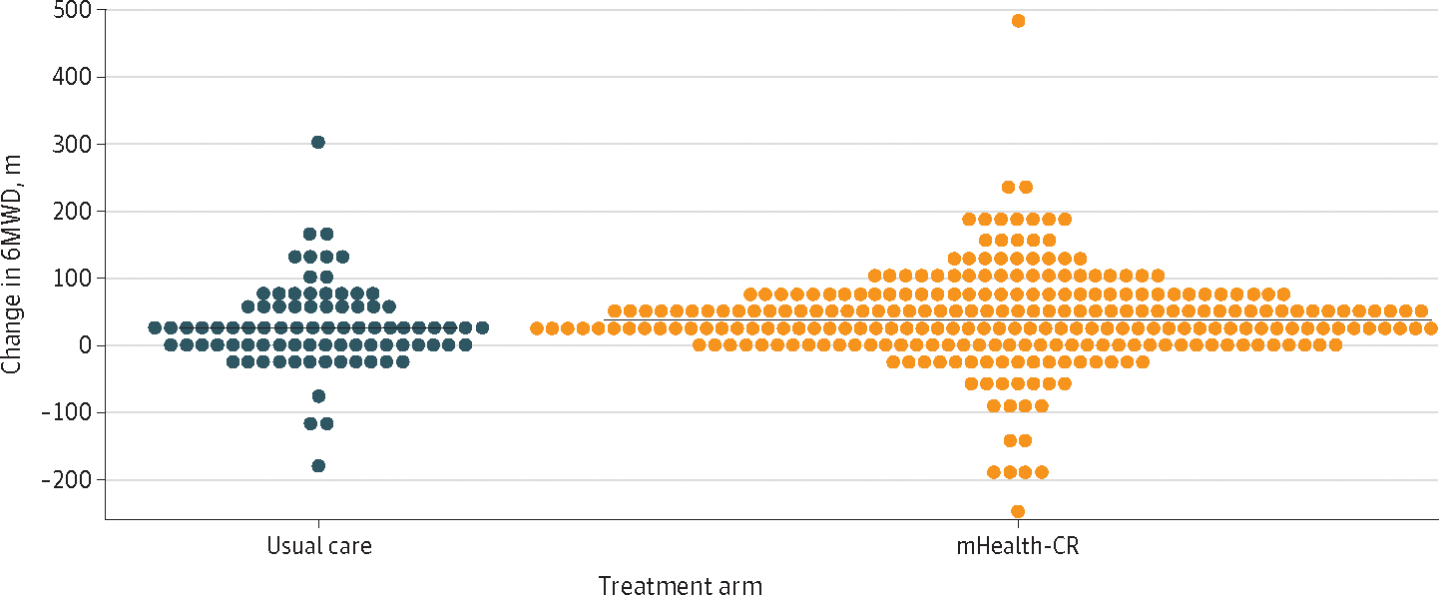
Change in 6-Minute Walk Distance (6MWD) From Baseline to 3 Months Horizontal lines represent mean change in 6MWD. Blue indicates usual care, and orange, mobile health– enabled cardiac rehabilitation (mHealth-CR).

**Figure 3.**
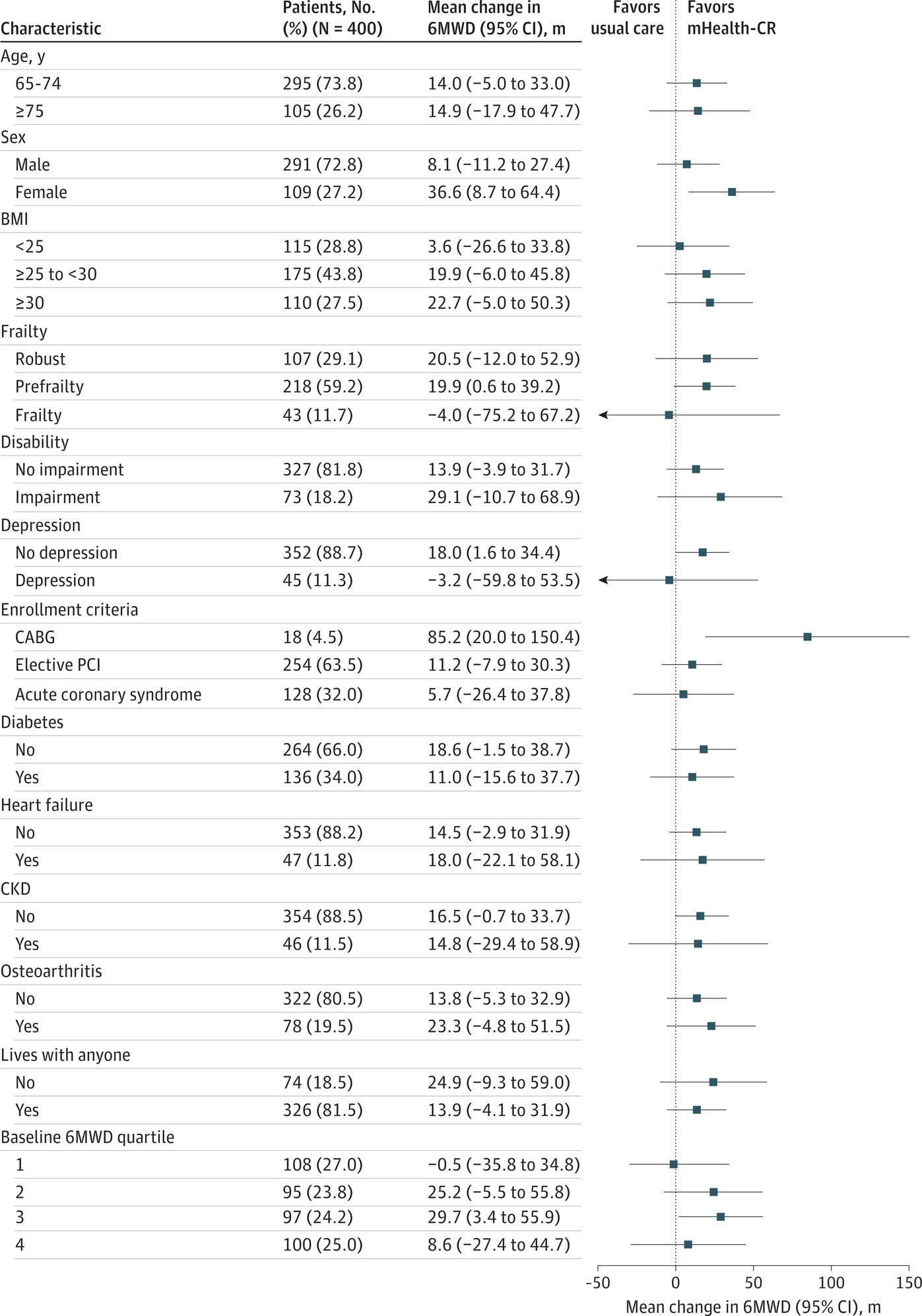
Forest Plot of Study Intervention Effect on 6-Minute Walk Distance (6MWD) by Subgroup There were 32 patients missing for frailty and 3 patients missing for depression. BMI indicates body mass index (calculated as weight in kilograms divided by height in meters squared); CABG, coronary artery bypass graft; CKD, chronic kidney disease; mHealth-CR, mobile health–enabled cardiac rehabilitation; PCI, percutaneous coronary intervention.

For the primary outcome of functional capacity, compared with usual care, participants who received mHealth-CR had no significant improvement in 6MWD at 3 months (mean [SD] change, 41.2 [1.5] m vs 25.6 [2.7] m; adjusted difference, 15.6 m [95% CI, −0.3 to 31.5 m]; *P* = .06) (Figure 2). The adjusted differences and 95% CIs for clinically relevant subgroups are shown in Figure 3. There appeared to be an effect of mHealth-CR vs usual care on 6MWD among women (improvement, 36.6 m; 95% CI, 8.7–64.4 m) and participants who had undergone CABG (improvement, 85.2 m; 95% CI, 20.0–150.4 m). However, the study was not powered to test for significance within these subgroups.

#### Secondary End Points

For general health status, mean (SD) 3-month SF-12 physical component score was 46.3 (1.0) points in the mHealth-CR arm and 48.2 points (1.1) in the control arm (adjusted difference, −1.9 points; 95% CI, −3.9 to 0.2 points) and mean (SD) SF-12 mental component scores were similar (51.5 [0.7] vs 51.6 [0.7]; adjusted difference, −0.1 [95% CI, −2.0 to 1.9]) (Table 2). The proportion of study participants with residual angina (SAQ-7 < 100) at 3 months, using imputed data, was similar in both groups (mean [SD], 25.7% [0.8%] vs 20.9% [1.7%]; odds ratio [OR], 1.32; 95% CI, 0.72–2.39). The proportion with ADL or IADL impairment at 3 months was similar in the mHealth-CR and usual care groups (mean [SD], 9.7% [0.6%] vs 10.6% [1.1%]; OR, 0.92; 95% CI, 0.43–1.96).

#### Bayesian Analysis

eFigure 2 in Supplement 2 displays the prespecified secondary bayesian analysis of change in 6MWD. The posterior median of the 6MWD change was 14.2 m (95% credible interval, −2.4 to 31.3 m). The posterior probability that the change was greater than 0 m was 96%, greater than 10 m was 71%, greater than 20 m was 25%, and greater than 25 m was 10%. Therefore, based on the posterior probability, there was a high chance of any difference in improvement but a low likelihood that improvement was clinically significant (>25 m change based on prior literature^29^).

#### Analysis by Receipt of Any CR

Per the trial protocol, participants could still be referred to traditional CR at the discretion of their cardiologist. We therefore performed an analysis of change in 6MWD between the mHealth-CR and usual care treatment arms stratified by attendance in traditional CR. There were 64 participants who attended traditional CR, with a median number of 16.5 sessions (range, 1–36 sessions). Based on a propensity score weighting analysis, there was no significant effect of mHealth-CR among those who did not attend traditional CR (change in 6MWD, 25.7 m; 95% CI, −8.7 to 60.2 m) (eTable 3 in Supplement 2).

### Safety

There were 19 of 298 participants (6.4%) in the mHealth-CR group and 4 of 102 participants (3.9%) in the usual care group who experienced hospitalizations. One hospitalization (0.3%) in the intervention group (traumatic fall) occurred during study-related exercise. There were no deaths in either the intervention group or the control group at 3 months. A full listing of adverse events is given in eTable 4 in Supplement 2.

### Engagement With Study Intervention

Participants in the mHealth-CR arm used the mHealth-CR app a median of 3 times per week (IQR, 0–5 times per week) and achieved a median of 6157 steps per day (IQR, 3810–8679 steps per day). There were a median of 10.5 (IQR, 7–12) telephone encounters with exercise therapists among mHealth-CR arm participants over the 3-month study duration. There were variable patterns of engagement among study participants, with a generalized decline in engagement over time, which is illustrated in eFigure 3 in Supplement 2. Based on available data from the activity monitor, 231 of 298 intervention patients (77.5%) achieved 150 minutes of exercise per week on average.

## Discussion

In this phase 2 randomized clinical trial, mHealth-CR (compared with usual care) did not improve 6MWD beyond a clinically meaningful threshold of 25 m among adults aged 65 years or older. The adjusted mean (SD) improvement with mHealth-CR was 41.2 (1.5) m compared with 25.6 (2.7) m in the usual care arm (mean difference, 15.6 m; 95% CI, −0.3 to 31.5 m). The secondary end points of quality of life, residual angina, and disability were also not meaningfully different between groups. Based on a propensity score weighting analysis, there was no improvement in 6MWD in the mHealth-CR group among those who did not attend traditional CR (25.7 m; 95% CI, −8.7 to 60.2 m), although this was an exploratory finding. To our knowledge, this is the largest randomized clinical trial to date of mHealth-CR in older adults.

The idea of remote cardiac rehabilitation is not new; several decades ago, telephone-based rehabilitation programs were studied in patients unwilling or unable to attend a facility-based program.^33^ More recently, the concept of mHealth-CR has been developed to include home-based programs that incorporate digital technologies that have the potential to provide a more interactive and engaging experience for patients. Several recent studies of remote CR (with or without mHealth components) have reported similar or better functional outcomes compared with traditional CR programs.^34,35^ For example, a pragmatic nonrandomized trial of 237 patients with ischemic heart disease at 3 Veterans Health Administration medical centers showed greater gains in 6MWD with remote CR at 3 months compared with a traditional program (95 m vs 41 m; *P* < .001).^35^

There are several key limitations to prior work that informed the design of RESILIENT. First, some investigators used nonrandomized designs,^9^ and therefore, outcomes were influenced by confounding. Second, many studies^5,8^ enrolled highly engaged patients who were committed to carrying out a remote or in-person exercise program to completion; these individuals are typically different from many patients seen in practice. Third, enrollment of older adults in prior studies has been considerably limited; in a 2021 meta-analysis of 14 randomized clinical trials comparing remote vs traditional CR, mean age was less than 65 years in all but 2 of them.^36^

We designed RESILIENT to address many of these limitations, particularly in light of older adults having both a high burden of ischemic heart disease and considerable barriers to traditional CR. While mHealth-CR may be appealing for this population due to its ease of use and scalability, mobile technology use among older adults still lags compared with that among younger individuals. This is due to barriers that include utility cost (frustration with technology, resistance to change), physical limitations (vision impairment, arthritis), cognitive challenges (poor memory, impaired reasoning), and overall limited digital health literacy.^37^ All of these factors may plausibly impede the uptake and effectiveness of mHealth-CR in an older adult population despite its theoretical benefits. Notably, 92.5% of RESILIENT trial participants had a high degree of multimorbidity (defined as 2 chronic medical conditions), and 65.2% were classified as having frailty or prefrailty using criteria adapted from Fried et al,^18^ which emphasizes that we captured a largely geriatric phenotype (rather than those who were simply chronologically aged 65 years).

Aside from our primary outcome, there are several other important findings from RESILIENT. First, serious adverse events were rare: only 1 participant in the intervention arm experienced an exercise-related fall that required hospitalization. Second, there were subgroups that achieved a clinically meaningful benefit, most notably women (improvement of 36.6 m; 95% CI, 8.7–64.4 m). However, this finding is exploratory and should be treated as hypothesis generating. Third, engagement with mHealth-CR in the intervention arm varied widely, with a generalized decline of engagement over the 3-month period. Similar patterns have been described with adherence to medications among older adults.^38^ This declining engagement may account for the overall negative findings in our study, and understanding predictors of engagement is an important area for future research.

### Limitations

There are several important limitations to our work. First, RESILIENT was designed as a pragmatic trial, and participants could still receive traditional CR at the discretion of their cardiologist as recommended by guidelines. There was greater receipt of traditional CR in the usual care arm, which introduced a potential source of postrandomization confounding. We made this decision so as not to deny standard of care to patients in light of limited evidence about the efficacy of mHealth-CR in older patients. Second, 72.2% of participants who were eligible declined informed consent, and there is a potential selection bias whereby healthier or more technologically literate individuals agreed to participate. This may limit the generalizability of our findings. Third, only 27.2% of the enrolled sample were women, although notably, this matched screening demographics at our participating institutions, and this proportion was higher than in a prior meta-analysis of remote CR (21.8% women).^36^ Fourth, our choice of a 25-m difference in 6MWD as representing a clinically meaningful change was not validated in exclusively older patients. However, we chose this value as it is a commonly cited threshold for ischemic heart disease^29^ and below the level of improvement reported by many traditional CR programs^24^; in our estimation, 25 m therefore represents a relatively small threshold to clear. Fifth, the early part of the RESILIENT trial took place during the first year of the COVID-19 pandemic, which likely influenced attendance rates at traditional CR programs and may also have influenced the decision of over two-thirds of eligible patients to decline informed consent. Finally, technology changes rapidly, and because RESILIENT started enrollment in 2019, we are unable to state whether newer technologies would benefit our target population. Notably, several ongoing randomized clinical trials, including the mTECH-Rehab trial^39^ and the MCNAIR study,^40^ will generate further effectiveness data about the capabilities of mHealth-CR. We look forward to these results, which will inform populations that may best benefit from mHealth-CR.

## Conclusions

In this randomized clinical trial of mHealth-CR vs usual care, mHealth-CR did not achieve a significant improvement in functional capacity among older adults with ischemic heart disease. There are several findings from our work, including improvement in functional capacity with mHealth-CR among women, that deserve further study. Our overall negative finding also suggests that more age-tailored mHealth strategies may be required to broadly improve functional outcomes for the older adult population.

## Supplementary Material

Supplement 1 Trial Protocol

Supplement 2 **eAppendix 1.** List of Study Sites for the RESILIENT Trial and Adverse Event Reporting eMethods
**eAppendix 2.** Additional Statistical Analysis Details
**eTable 1.** Characteristics of Participants With Completed vs Missing Measurement of Primary End Point at 3 Months
**eTable 2.** Missing End Point Data by Study Site
**eTable 3.** Analysis by Receipt of Any CR
**eTable 4.** Adverse Events
**eFigure 1.** Sample Layout of mHealth-CR Software
**eFigure 2.** Results of Bayesian Analysis
**eFigure 3.** mHealth-CR Engagement Among Intervention Arm Participants

Supplement 3 Data Sharing Statement

## Figures and Tables

**Table 1.** Demographic and Clinical Characteristics at Baseline

Characteristic	Participants (N = 400)^a^	
	mHealth-CR (n = 298)	Usual care (n = 102)
Age, median (range), y	71.0 (65.0–91.0)	71.0 (65.0–89.0)
Sex		
Men	216 (72.5)	75 (73.5)
Women	82 (27.5)	27 (26.5)
Race^b^		
Asian	11 (3.7)	6 (5.9)
Black	27 (9.1)	9 (8.8)
White	229 (76.8)	74 (72.5)
Multiracial or other	31 (10.4)	13 (12.7)
Ethnicity		
Hispanic	23 (7.7)	11 (10.8)
Non-Hispanic	275 (92.3)	91 (89.2)
Hypertension	254 (85.2)	83 (81.4)
Diabetes	94 (31.5)	42 (41.2)
Heart failure	34 (11.4)	13 (12.7)
Atrial fibrillation	36 (12.1)	11 (10.8)
Chronic lung disease	40 (13.4)	13 (12.7)
BMI, median (range)	27.3 (14.7–46.8)	27.7 (18.4–44.4)
EGFR, mL/min/1.73 m^2^		
Mean (SD)	76.9 (24.2)	75.3 (16.7)
<30	6 (2.0)	0
30–59	41 (13.8)	21 (20.6)
≥60	251 (84.2)	81 (79.4)
History of any tobacco use	75 (25.2)	22 (21.6)
ADL or IADL impairment	54 (18.1)	19 (18.6)
Frailty^c^		
Frailty	37 (12.4)	6 (5.9)
Prefrailty	154 (51.7)	64 (62.7)
Robust	82 (27.5)	25 (24.5)
Missing	25 (8.4)	7 (6.9)
Depressive symptoms^d^	35 (11.7)	10 (9.8)
Medication use at discharge		
β-Blocker	203 (68.1)	69 (67.6)
Calcium channel blocker	86 (28.9)	25 (24.5)
Long-acting nitrate	40 (13.4)	9 (8.8)
Aspirin	272 (91.3)	93 (91.2)
P2Y12 inhibitor	275 (92.3)	92 (90.2)
Statin	278 (93.3)	91 (89.2)
Enrollment criteria		
Acute coronary syndrome		
Acute myocardial infarction with PCI	74 (24.8)	22 (21.6)
Acute myocardial infarction without PCI	7 (2.3)	2 (2.0)
Unstable angina with PCI	18 (6.0)	5 (4.9)
Elective PCI	186 (62.4)	68 (66.7)
Coronary artery bypass graft	13 (4.4)	5 (4.9)

Abbreviations: ADL, activity of daily living; BMI, body mass index (calculated as weight in kilograms divided by height in meters squared); EGFR, estimated glomerular filtration rate; IADL, instrumental activity of daily living; mHealth-CR, mobile health-enabled cardiacrehabilitation; PCI, percutaneous coronary intervention.

a Data are presented as number (percentage) of participants unless otherwise indicated.

b Based on self-identified category. Other includes American Indian or Alaska Native, Native Hawaiian or Other Pacific Islander, or participants self-selecting "other" without further specification.

c Defined using criteria from Fried et al.^18^

d Based on the 8-item Patient Health Questionnaire.^17^

**Table 2.** Study Outcomes

Outcome^a^	mHealth-CR (n = 298)	Usual care (n = 102)	Treatment effect (95% CI)^b^
Primary outcome			
Change in 6MWD, mean (SD), m	41.2 (1.5)	25.6 (2.7)	15.6 (−0.3 to 31.5)^c^
Secondary outcomes			
SF-12 physical component score, mean (SD)	46.3 (1.0)	48.2 (1.1)	−1.9 (−3.9 to 0.2)
SF-12 mental component score, mean (SD)	51.5 (0.7)	51.6 (0.7)	−0.1 (−2.0 to 1.9)
Residual angina, mean (SD), %^d^	25.7(0.8)	20.9 (1.7)	1.32 (0.72 to 2.39)
Any ADL or IADL impairment, mean (SD), %	9.7 (0.6)	10.6 (1.1)	0.92 (0.43 to 1.96)

Abbreviations: 6MWD, 6-minute walk distance; ADL, activity of daily living; IADL, instrumental activity of daily living; mHealth-CR, mobile health-enabled cardiac rehabilitation; SF-12,12-Item Short Form Health Survey.

a Rates are based on estimates from the model using imputed data.

b Treatment effects are shown as adjusted mean differences (adjusted for baseline instrument measure, age, presence of diabetes, and study site) for 6MWD, SF-12 physical component score, and SF-12 mental component score and as odds ratios for residual angina and ADL or IADL impairment.

c *P* = .06. Significance testing was performed for the primary outcome only, as the trial was not powered for multiple comparisons.

d Score of less than 100 on the 7-Item Seattle Angina Questionnaire (total score range, 0-100, with higher scores indicating less frequent angina).

## Data Availability

See Supplement 3.

## Key Points

### Question

Does mobile health cardiac rehabilitation (mHealth-CR) improve functional capacity among older adults with ischemic heart disease?

### Findings

In this randomized clinical trial of 400 adults aged 65 years or older with ischemic heart disease, receipt of mHealth-CR vs usual care did not significantly improve functional capacity beyond a clinically meaningful threshold of 25 m in 6-minute walk distance.

### Meaning

The findings suggest that mHealth-CR interventions may not benefit the older adult population and more age-tailored strategies may be required.
