# Supplementary material for: Rehabilitation at Home Using Mobile Health for Older Adults Hospitalized for Ischemic Heart Disease: The RESILIENT Randomized Clinical Trial
Source: JAMA Netw Open. Author manuscript; Available in PMC 2025 Jul 11. (PMC12247035; doi:10.1001/jamanetworkopen.2024.53499)
Supplement: Supplement 1 — Trial Protocol [file NIHMS2086641-supplement-Supplement_1.pdf]

NYU School of Medicine
NYU LANGONE MEDICAL CENTER

RESILIENT

A phase II, 3:1 randomized open-label, prospective, pragmatic, multi-center clinical study evaluating the effects of mobile health cardiac rehabilitation (mHealth-CR) on participants age ≥65 years diagnosed with ischemic heart disease (IHD).

**Principal Investigator:**	John A. Dodson, MD, MPH<br>Department of Medicine – NYU School of Medicine<br>Leon H. Charney Division of Cardiology<br>227 E. 30th Street, New York, NY 10016<br>John.Dodson@nyulangone.org<br>646-501-2714
**Additional Investigators:**	Samrachana Adhikari, PhD<br>Department of Population Health – NYU School of Medicine<br>180 Madison Ave, New York, NY 10016<br>samrachana.adhikari@nyulangone.org<br>646-501-3647<br><br>Caroline Blaum, MD, MS<br>Department of Geriatrics – NYU School of Medicine<br>530 1st Avenue, HCC Suite 7B, New York, NY 10016<br>Caroline.Blaum@nyulangone.org<br>646-501-2325<br><br>Antoinette Schoenthaler, EdD, FAACH<br>Department of Population Health – NYU School of Medicine<br>227 E. 30th Street, New York, NY 10016<br>Antoinette.Schoenthaler@nyulangone.org<br>646-501-3434<br><br>Sarwat Chaudhry, MD<br>Department of Medicine – Yale School of Medicine<br>367 Cedar St. Harkness A Building, PO Box 208093,<br>New Haven, CT 06520-8093<br>sarwat.chaudhry@yale.edu<br><br>Thomas Gill, MD<br>Department of Geriatrics – Yale School of Medicine<br>874 Howard Avenue, New Haven, CT 06519<br>thomas.gill@yale.edu<br><br>Lara C. Kovell, MD<br>University of Massachusetts School of Medicine<br>55 Lake Avenue North, Worcester, MA, 01650<br>Lara.kovell@umassmemorial.org
**NYULMC Study Number:**	s18-02017
**Funding Sponsor:**	National Institutes of Health/National Institutes of Aging
**Study Device Provider:**	Moving Analytics (San Francisco, CA), FitBit (San Francisco, CA), BodyTrace (Palo Alto, CA), Omron (Kyoto, Japan)
**ClinicalTrials.gov Number**	NCT03978130

CONFIDENTIAL
This material is the property of the NYU School of Medicine and Langone Medical Center. Do not disclose or use except as authorized in writing by the study sponsor

Initial version: 3.0 [1/30/19]
Amended: v 4.0 11/26/2019
Amended: v 5.0 2/24/20
Amended: v 6.0 8/14/20
Amended v 7.0 12/01/20
Amended v 8.0 01/06/21
Amended v 9.0 03/31/21
Amended v 10.0 07/16/21
Amended v 11.0 09/10/21
Amended v 12.0 12/03/21
Amended v 13.0 02/02/22
Amended v 14.0 01/26/23
Amended v 15.0 01/30/23

CONFIDENTIAL
This material is the property of the NYU School of Medicine and Langone Medical Center. Do not disclose or use except as authorized in writing by the study sponsor

Statement of Compliance

This study will be conducted in accordance with the Code of Federal Regulations on the Protection of Human Subjects (45 CFR Part 46), 21 CFR Parts 50, 56, 312, and 812 as applicable, any other applicable US government research regulations, and institutional research policies and procedures. The International Conference on Harmonisation ("ICH") Guideline for Good Clinical Practice ("GCP") (sometimes referred to as "ICH-GCP" or "E6") will be applied only to the extent that it is compatible with FDA and DHHS regulations. The Principal Investigator will assure that no deviation from, or changes to the protocol will take place without prior agreement from the sponsor and documented approval from the Institutional Review Board (IRB), except where necessary to eliminate an immediate hazard(s) to the trial participants. All personnel involved in the conduct of this study have completed Human Subjects Protection Training.

CONFIDENTIAL
This material is the property of the NYU School of Medicine and Langone Medical Center. Do not disclose or use except as authorized in writing by the study sponsor

Table of Contents

STATEMENT OF COMPLIANCE .... **II**
LIST OF ABBREVIATIONS .... **VI**
PROTOCOL SUMMARY **7**
SCHEMATIC OF STUDY DESIGN **8**
1 KEY ROLES **9**
2 INTRODUCTION, BACKGROUND INFORMATION AND SCIENTIFIC RATIONALE **10**
2.1 Background Information and Relevant Literature 10
2.2 Rationale 10
2.3 Potential Risks & Benefits 11
2.3.1 Known Potential Risks *11*
2.3.2 Known Potential Benefits *12*
3 OBJECTIVES AND PURPOSE **13**
3.1 Primary Objective 13
3.2 Secondary Objectives 13
4 STUDY DESIGN AND ENDPOINTS **13**
4.1 Description of Study Design 13
4.2 Study Endpoints 13
4.2.1 Primary Study Endpoints *13*
4.2.2 Secondary Study Endpoints *14*
4.2.3 Exploratory Endpoints *15*
5 STUDY ENROLLMENT AND WITHDRAWAL **15**
5.1 Inclusion Criteria 15
5.2 Exclusion Criteria 15
5.3 Vulnerable Subjects 16
5.4 Strategies for Recruitment and Retention 16
5.4.1 Use of Epic Information for Recruitment Purposes *17*
5.5 Duration of Study Participation 18
5.6 Total Number of Participants and Sites 18
5.7 Participant Withdrawal or Termination 18
5.7.1 Reasons for Withdrawal or Termination *18*
5.7.2 Handling of Participant Withdrawals or Termination *19*
5.7.3 Premature Termination or Suspension of Study *19*
6 BEHAVIORAL/SOCIAL INTERVENTION **19**
6.1 Study Behavioral or Social Intervention(s) Description 19
6.1.1 Administration of Intervention *19*
6.1.2 Procedures for Training Interventionalists and Monitoring Intervention Fidelity *20*
6.1.3 Assessment of Subject Compliance with Study Intervention *21*
7 STUDY PROCEDURES AND SCHEDULE **22**
7.1 Study Procedures/Evaluations 22
7.1.1 Study Specific Procedures *22*
7.1.2 Standard of Care Study Procedures *25*
7.2 Study Schedule 25
7.2.1 Screening *25*
7.2.2 Enrollment/Baseline *26*
7.2.3 Intermediate Visits *27*

This material is the property of the NYU School of Medicine and Langone Medical Center. Do not disclose or use except as authorized in writing by the study sponsor

7.2.4 Final Study Visit *27*
7.2.5 Withdrawal/Early Termination Visit *27*
7.2.6 Unscheduled Visit *27*

8 ASSESSMENT OF SAFETY 27

8.1 Specification of Safety Parameters 27
8.1.1 Definition of Adverse Events (AE) *27*
8.1.2 Definition of Serious Adverse Events (SAE) *28*
8.1.3 Definition of Unanticipated Problems (UP) *28*
8.2 Classification of an Adverse Event 28
8.2.1 Severity of Event *28*
8.2.2 Relationship to Study Intervention *29*
8.2.3 Expectedness *29*
8.3 Time Period and Frequency for Event Assessment and Follow-Up 29
8.4 Reporting Procedures – Notifying the IRB 30
8.4.1 Adverse Event Reporting *30*
8.4.2 Serious Adverse Event Reporting *30*
8.4.3 Unanticipated Problem Reporting *30*
8.4.4 Reporting of Pregnancy *31*
8.5 Reporting Procedures – Notifying the Study Sponsor 31
8.6 Reporting Procedures – Participating Investigators 31
8.7 Study Halting Rules 31
8.8 Safety Oversight 32
8.8.1 Events/Data to be Reviewed by DSMB *32*
8.8.2 Pre-Specified Stopping Rules *32*
8.8.3 Dissemination of Decision by DSMB *33*

9 CLINICAL MONITORING 33

10 STATISTICAL CONSIDERATIONS 33

10.1 Statistical and Analytical Plans (SAP) 33
10.2 Statistical Hypotheses 33
10.3 Analysis Datasets 34
10.4 Description of Statistical Methods 34
10.4.1 General Approach *34*
10.4.2 Analysis of the Primary Efficacy Endpoint(s) *34*
10.4.3 Analysis of the Secondary Endpoint(s) *35*
10.4.4 Safety Analyses *35*
10.4.5 Adherence and Retention Analyses *36*
10.4.6 Baseline Descriptive Statistics *37*
10.4.7 Planned Interim Analysis *37*
10.4.8 Additional Sub-Group Analyses *37*
10.4.9 Multiple Comparison/Multiplicity *37*
10.4.10 Tabulation of Individual Response Data *37*
10.4.11 Exploratory Analyses *37*
10.5 Sample Size 38
10.6 Measures to Minimize Bias 39
10.6.1 Enrollment/Randomization/Masking Procedures *39*
10.6.2 Evaluation of Success of Blinding *40*
10.6.3 Breaking the Study Blind/Participant Code *40*

11 SOURCE DOCUMENTS AND ACCESS TO SOURCE DATA/DOCUMENTS 40

12 QUALITY ASSURANCE AND QUALITY CONTROL 40

13 ETHICS/PROTECTION OF HUMAN SUBJECTS 41

13.1 Ethical Standard 41
13.2 Institutional Review Board 41

This material is the property of the NYU School of Medicine and Langone Medical Center. Do not disclose or use except as authorized in writing by the study sponsor

13.3 INFORMED CONSENT PROCESS ... 41
13.3.1 Consent/Assent and Other Informational Documents Provided to Participants ... 41
13.3.2 Consent Procedures and Documentation ... 41
13.4 PARTICIPANT AND DATA CONFIDENTIALITY ... 42
13.4.1 Research Use of Stored Human Samples, Specimens, or Data ... 43

14 DATA HANDLING AND RECORD KEEPING ... 43

14.1 DATA COLLECTION AND MANAGEMENT RESPONSIBILITIES ... 43
14.2 STUDY RECORDS RETENTION ... 44
14.3 PROTOCOL DEVIATIONS ... 44
14.4 PUBLICATION AND DATA SHARING POLICY ... 44

15 STUDY FINANCES ... 45

15.1 FUNDING SOURCE ... 45
15.2 COSTS TO THE PARTICIPANT ... 45
15.3 PARTICIPANT REIMBURSEMENTS OR PAYMENTS ... 45

16 STUDY ADMINISTRATION ... 45

16.1 STUDY LEADERSHIP ... 45

17 CONFLICT OF INTEREST POLICY ... 46

18 REFERENCES ... 46

19 ATTACHMENTS ... 53

This material is the property of the NYU School of Medicine and Langone Medical Center. Do not disclose or use except as authorized in writing by the study sponsor

List of Abbreviations

6MWD/T	6-Minute Walking Distance/Test
AE	Adverse Event/Adverse Experience
AMI	Acute Myocardial Infarction
CABG	Coronary Artery Bypass Graft
CFR	Code of Federal Regulations
CR	Cardiac Rehabilitation
CRF	Case Report Form
DCC	Data Coordinating Center
DHHS	Department of Health and Human Services
DSMB	Data and Safety Monitoring Board
EHR	Electronic Health Records
GAS	Goal Attainment Scoring
GCP	Good Clinical Practice
HIPAA	Health Insurance Portability and Accountability Act
ICF	Informed Consent Form
ICH	International Conference on Harmonisation
IHD	Ischemic Heart Disease
IRB	Institutional Review Board
mHealth-CR	Mobile health cardiac rehabilitation
MOP	Manual of Procedures
N	Number (typically refers to participants)
NIH	National Institutes of Health
OHRP	Office for Human Research Protections
OHSR	Office of Human Subjects Research
PCI	Percutaneous Coronary Intervention
PHQ-8	Patient Health Questionnaire (8 questions)
PI	Principal Investigator
QC	Quality Control
SAE	Serious Adverse Event/Serious Adverse Experience
SAQ-7	Seattle Angina Questionnaire (Short Version)
SF-12	12-Item Short Form Survey
SOP	Standard Operating Procedure
UBACC	University of California, San Diego Brief Assessment of Capacity to Consent
US	United States

CONFIDENTIAL
This material is the property of the NYU School of Medicine and Langone Medical Center. Do not disclose or use except as authorized in writing by the study sponsor

Protocol Summary

Title	REhabilitation at home uSIng mobiLe health In oldEr adults after hospitalizatioN for ischemic hearT disease (RESILIENT)
Short Title	RESILIENT
Brief Summary	This study aims to evaluate whether mHealth-CR improves functional capacity in older adults (age ≥65) with IHD compared to usual care. Both groups will also receive a referral to (but not mandatory attendance of) traditional CR. A total of 400 eligible patients will be randomized in 3:1 manner to mHealth-CR versus usual care for assessment of primary endpoint. Enrollment will occur over approximately 42 months with an expected minimum of 3 months follow-up per participant.
Phase	Phase 3
Objectives	The study includes objectives related to both efficacy and engagement. The primary efficacy objective is to evaluate whether mHealth-CR improves functional capacity in older adults (age ≥65) with IHD compared with usual care alone. Secondary efficacy objectives are to evaluate whether mHealth improves: (1) goal attainment; (2) health status, (3) basic and instrumental activities of daily living, (4) hospital readmission, and (5) death in participants compared to usual care. The primary engagement objective is to characterize the engagement of participants with mHealth-CR and to elucidate barriers to participation.
Methodology	Open label, randomized clinical trial with blinded assessment of the primary endpoint.
Endpoint	Primary endpoint (efficacy) is functional capacity, measured by the change in 6 Minute Walk Distance (6MWD). Secondary endpoints (efficacy) are: (1) goal attainment, measured by goal attainment scoring (GAS); (2) change in reported health status, measured by SF-12 and SAQ-7; (3) changes in activities of daily living; (4) hospital readmission; and (5) death. Primary endpoint (engagement) is based on patterns of mHealth-CR use over study duration.
Study Duration	53 months (Month 7, Year 1 – Month 60, Year 5)
Participant Duration	3 months (from baseline visit to follow-up visit)
Duration of IP administration	42 months (throughout participant enrollment)
Population	Older adults (age ≥65) diagnosed with IHD within the New York, New Haven and Massachusetts metropolitan areas.
Study Sites	NYU Langone Health System, UMass Memorial Health Care (UMMHC), Bellevue Hospital, and Yale New Haven Health
Number of participants	400 participants expected to be enrolled across 2 sites.
Description of Study Devices	Devices provided to intervention arm only: portable tablet (Samsung Galaxy); activity tracking device (FitBit), blood pressure cuff (BodyTrace, Palo Alto, CA, Omron Kyoto, Japan), mHealth-CR software application (Moving Analytics, San Francisco, CA)
Reference Therapy	Usual care, which includes standard therapies post-AMI as deemed clinically appropriate. Both intervention and usual care arms will receive a referral to traditional ambulatory CR in accordance with best practice. However, attendance at ambulatory CR will not be facilitated or mandated by the study in the interest of a pragmatic trial.

CONFIDENTIAL
This material is the property of the NYU School of Medicine and Langone Medical Center. Do not disclose or use except as authorized in writing by the study sponsor

Statistical Analysis	Primary endpoint (efficacy): Change in 6WMD will be assessed by calculating difference scores for each participant and comparing the intervention and usual care groups with an independent group t-test. Regression of 3-month 6MWD on a binary indicator of treatment group will also be measured, with adjustment for baseline 6MWD and the stratification factor (enrollment site). Secondary endpoint (efficacy): Differences in secondary endpoints will be analyzed by the appropriate statistical test for continuous or categorical measures. Primary endpoint (engagement): Measurements of engagement will use latent class analysis to identify profiles of engagement and assess trajectories.

Schematic of Study Design

Flow Diagram

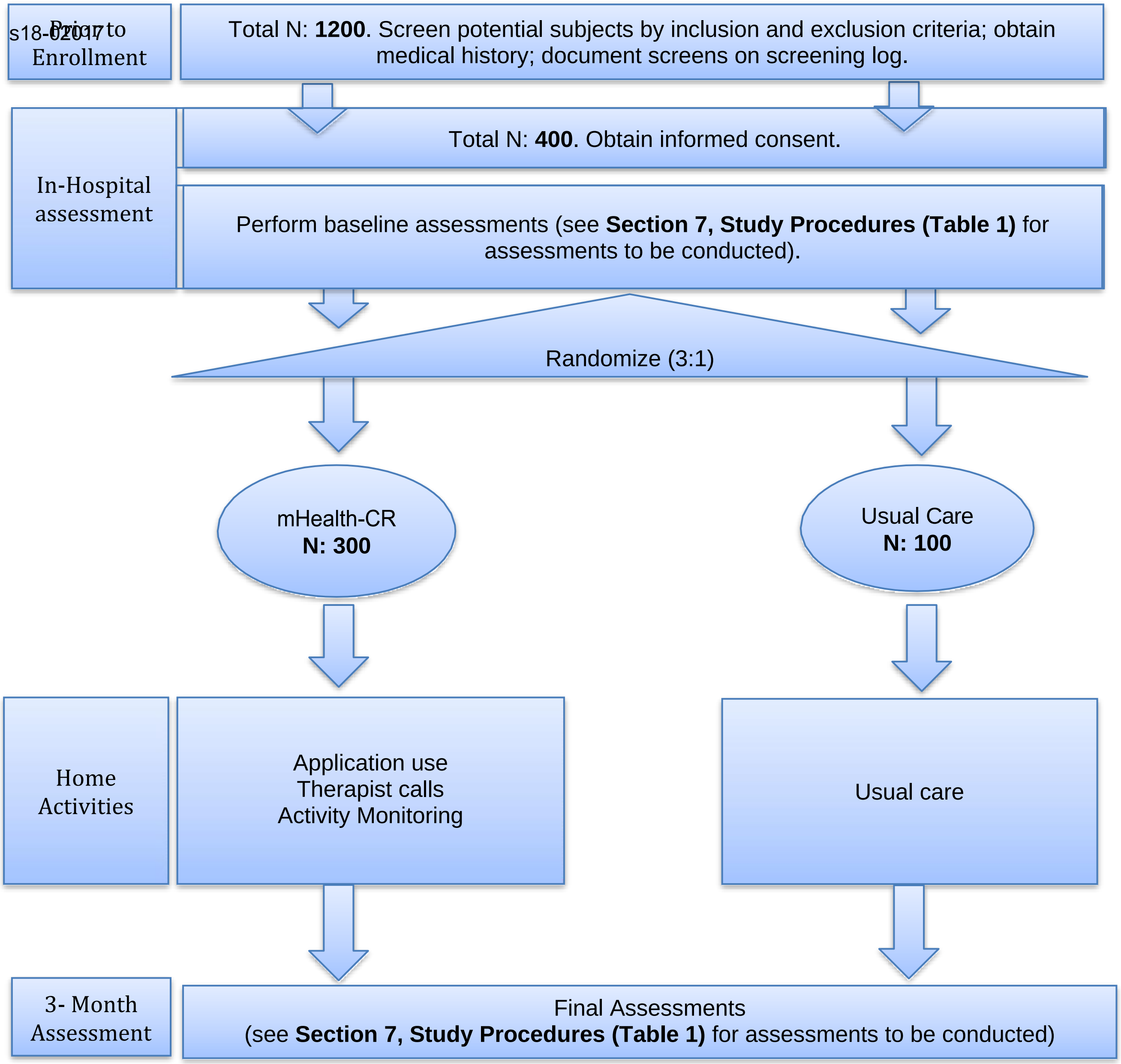

1 Key Roles

John A. Dodson, MD, MPH – Principal Investigator
Leon H. Charney Division of Cardiology –NYU School of Medicine
227 E. 30th Street, New York, NY 10016
John.Dodson@nyulangone.org

Kevin P. Marzo, MD – Sub-Investigator
Center for Cardiovascular Lifestyle Medicine – NYU Winthrop Hospital
212 Jericho Turnpike, Mineola, NY 115011

Barbara J. George EdD, RCEP, MSN, AGNP-C – Sub-Investigator
Center for Cardiovascular Lifestyle Medicine – NYU Winthrop Hospital
212 Jericho Turnpike, Mineola, NY 115011

Samrachana Adhikari, PhD –Co-Investigator, Biostatistician
Department of Population Health – NYU School of Medicine
180 Madison Ave, New York, NY 10016
samrachana.adhikari@nyulangone.org

Patrick Xin – Data Management, NYU DataCore
Department of Population Health – NYU School of Medicine
360 Park Avenue South, Room 7-206, New York, NY 10010
Patrick.Xin@nyumc.org

Caroline Blaum, MD, MS – Co-Investigator
Department of Geriatrics –NYU School of Medicine
530 1st Avenue, HCC Suite 7B, New York, NY 10016
Caroline.Blaum@nyulangone.org

Antoinette Schoenthaler, EdD – Co-Investigator
Department of Population Health – NYU School of Medicine
227 E. 30th Street, New York, NY 10016
Antoinette.Schoenthaler@nyulangone.org

Judith S. Hochman, MD – Co-Investigator
Leon H. Charney Division of Cardiology –NYU School of Medicine
227 E. 30th Street, New York, NY 10016
Judith.hochman@nyulangone.org

Sarwat Chaudhry, MD—Yale Site Principal Investigator
Yale School of Medicine
367 Cedar St. Harkness A Building, New Haven, CT 06520
sarwat.chaudhry@yale.edu

Thomas Gill, MD –Yale Site Co-Investigator
Yale School of Medicine
874 Howard Avenue, New Haven, CT 06519
thomas.gill@yale.edu

Lee Jennings, MD, MSHS –Consultant, Goal Attainment Scaling
University of Oklahoma Health Sciences Center
1122 N.E. 13th Street, ORB 1200, Oklahoma City, OK 73117
lee-jennings@ouhsc.edu

CONFIDENTIAL
This material is the property of the NYU School of Medicine and Langone Medical Center. Do not disclose or use except as authorized in writing by the study sponsor

JP Pollak, PhD –Consultant, Computer Science
Cornell Tech
2 W Loop Rd, New York, NY 10044
jpp9@cornell.edu

Lara Kovell, MD – UMMHC Site Principal Investigator
University of Massachusetts School of Medicine
55 Lake Avenue North Worcester, MA, 01650
Lara.Kovell@umassmemorial.org

2 Introduction, Background Information and Scientific Rationale

2.1 Background Information and Relevant Literature

Among older adults with ischemic heart disease (IHD), participation in ambulatory cardiac rehabilitation (CR) remains stubbornly low despite decades of evidence about its benefits; by recent estimates, fewer than two-thirds of eligible patients are referred, and even among those referred only half attend the first session.[1–3] In addition to barriers faced by the general population (limited facilities, competing time demands, high out-of-pocket costs, prolonged wait time for space), older adults face additional particular barriers including lack of transportation, physical limitations, and cognitive impairments that make it especially difficult to use existing CR paradigms.[4,5] Therefore, while older adults may have the greatest potential to benefit from CR because of their higher risk of adverse IHD-related sequelae, they are also the least likely to participate.[4,6,7] In addition, it is unclear whether the usual goals set by ambulatory CR programs (e.g. risk factor modification, improvement in aerobic capacity) are aligned with older patients' goals, which often include outcomes such as independence and preservation of physical function.[8,9]

Mobile health-enabled CR (mHealth-CR) for IHD, which involves delivery of rehabilitation via portable electronic devices, has proliferated rapidly in recent years.[5,10,11] mHealth-CR programs are variable but typically include exercise documentation, hemodynamic assessment, video education, and electronic communication with an exercise therapist; these may be stand-alone components or an adjunct to traditional ambulatory CR programs.[5,11] Professional guidelines recommend that CR be technically innovative and patient-centered,[22] and mHealth-CR provides a novel opportunity to meet these goals; mHealth-CR involves mobile device-based software for patients to document exercise, control risk factors (e.g., blood pressure [BP]), access education materials, review physical activity, and communicate electronically with clinicians. mHealth-CR, used as a standalone intervention or as an adjunct to traditional CR,[5,10] promises to promote physical activity and self-care skills, and to allow outreach to those who cannot access traditional CR due to geographical or financial barriers.[23]

2.2 Rationale

While mHealth-CR has the potential to increase engagement by reducing participation barriers, it remains largely untested outside of small studies in relatively young, healthy persons. It is therefore unclear what proportion of older adults with IHD and barriers to traditional CR (who may benefit the most) are able to engage with mHealth-CR, and whether mHealth-CR in addition to usual care leads to better outcomes than usual care in this population. Further, to our knowledge, mHealth-CR has never been individualized to align with the unique functional limitations and goals of older adults with IHD.

We therefore propose to conduct a clinical trial within two academic health systems to evaluate mHealth-CR in patients age ≥65 years. In this trial embedded within the current standard of care, patients in both arms will receive a standardized referral to traditional ambulatory CR at hospital discharge, in accordance with professional guidelines[2] (typically started within 4 weeks at our institutions). Intervention arm participants will also receive mHealth-CR, initiated immediately, for 3 months.

This material is the property of the NYU School of Medicine and Langone Medical Center. Do not disclose or use except as authorized in writing by the study sponsor

Our central hypotheses related to efficacy are that mHealth-CR will (1) improve functional capacity (primary outcome), (2) improve goal attainment, health status, and activities of daily living, and (3) lower rates of hospital readmission and death (secondary outcomes). We expect the benefits of mHealth-CR to mirror those of traditional ambulatory CR on cardiorespiratory fitness and risk factor modification, which is grounded in behavior change theory.[30,31] Implementation of mHealth-CR has the potential to engage patients earlier than traditional CR, and with more frequent interaction, while simultaneously reducing barriers such as transportation and cost.

Our central hypothesis related to engagement is that we will identify distinct trajectories of engagement and characteristics that predict membership in each category. By design, we will only approach patients over age 65. While we expect that mHealth-CR will increase access, residual barriers are likely. Our hypothesis is driven by the Technology Acceptance Model v.3 (TAM3),[32] based on the Theory of Reasoned Action, which asserts that perceptions of usefulness and ease of use influence adoption of a new technology. As with other technologies, selected subgroups are likely to have distinct engagement patterns,[33–35] although these characteristics with mHealth-CR are as yet undefined. Our study therefore provides necessary foundational work needed to understand which patients may be optimal candidates for future mHealth interventions.

mHealth-CR represents an innovative approach to behavior change that takes advantage of mobile devices and combines remote monitoring technology with personalized feedback to improve patient care. This intervention has the potential to overcome existing barriers to CR utilization by engaging patients in their daily lives to promote physical activity and other healthy lifestyle behaviors. Because older adults may experience challenges with the technology, our design couples mHealth-CR with personal contact by an exercise therapist; we believe this hybrid approach may be particularly successful in engaging older adults.

2.3 Potential Risks & Benefits

2.3.1 Known Potential Risks

Submaximal exercise testing. The 6-minute walk test (6MWT) is a submaximal exercise test in which the pace is self-selected by the participant. Theoretical risks include hypotension (drop in systolic BP ≥15 mmHg), angina, arrhythmia, dyspnea, leg cramps, and falls. For purposes of our study, we have operationalized the definition of IHD as either acute myocardial infarction (AMI), percutaneous coronary intervention (PCI), or coronary artery bypass graft (CABG); the AMI group (which in practice, may undergo PCI or CABG) is theoretically at higher risk during 6MWT. Patients undergoing elective PCI or elective CABG (by definition, not with AMI) are at lower risk, since they have not experienced an acute ischemic event. While guidance from the American Thoracic Society published 15 years ago and often cited for instructions on performing the test[52] recommended against performing submaximal exercise testing within 1 month of AMI, guidance from the American College of Cardiology and American Heart Association stated that such testing was acceptable prior to hospital discharge.[113] Multiple subsequent studies have shown that events during 6MWT in patients hospitalized for IHD-related sequelae (including AMI) are rare in practice. Peixoto and colleagues reported that among 88 patients with AMI who completed 6MWT prior to hospital discharge, there were no adverse events.[62] Oliveira performed 6MWT on 60 patients who underwent cardiac surgery (including CABG for IHD) prior to discharge, and reported that all participants successfully completed the test with no adverse events.[61] In a more recent study, Diniz et al. performed 6MWT in 152 patients with AMI prior to discharge, and found that 3.9% of patients experienced either angina, hypotension, or arrhythmia (ventricular tachycardia), although all of these resolved upon cessation of the test.[114] Understandably in these studies there may have been a selection bias whereby more acutely ill patients were excluded from enrollment.

Accordingly, for our study, patients may be excluded from participation by their treating physician on any clinical grounds which the physician deems would potentially lead to safety issues. Study entry criteria to exclude potential participants at greater risk of adverse events, including conditions at increased fall risk (Parkinson's disease or other progressive neurologic movement disorder, severe osteoarthritis, recent orthopedic surgery). In addition, since the baseline 6MWT will be performed in the hospital, emergency equipment and personnel are readily available to deal with unusual situations should they arise.

Home-based cardiac rehabilitation. Theoretical risks of home-based cardiac rehabilitation involve risks

This material is the property of the NYU School of Medicine and Langone Medical Center. Do not disclose or use except as authorized in writing by the study sponsor

attributable to study-related exercise, which may occur at any point during the 3-month intervention period. We have defined major adverse events as the following: (1) Fall-related injury (operationalized as any fall requiring acute medical care); (2) Hospitalization for acute coronary syndrome (symptom onset within 30 minutes of exercise); (3) Hospitalization for unstable arrhythmia (symptom onset within 30 minutes of exercise). Based on decades of research on home-based CR interventions, adverse events are rare. Ades et al. reported that out of 3100 home-based rehab sessions, 7 sessions (0.2%) were canceled for symptoms (low-level angina, dyspnea, or arrhythmia), although none of these led to serious adverse consequences.[74] Jolly et al. reported zero adverse events in 263 participants for the duration of a 3-month home-based cardiac rehabilitation program.[73] Nonetheless, we understand the importance of active surveillance for potential study related adverse events in our enrolled participants, especially given that by definition we are limiting our sample to older adults (age ≥65).

To minimize risk, all procedures will be performed in clinically stable participants who meet eligibility criteria under the direct supervision of the Principal Investigator and other research personnel with appropriate training and licensure. The baseline visit will be performed in-hospital, and the 3-month follow-up visit will be performed in dedicated space adjacent to the outpatient cardiology Faculty Group Practice, on the main campus of NYU Langone Health (550 First Avenue, New York, NY). For NYULH-Winthrop participants, this will be at the outpatient office of the FGP of Winthrop Cardiology Associates located at 212 Jericho Turnpike (2nd floor) Mineola, New York. For UMMHC participants, this will be at the Ambulatory Care Center (1st floor) located at 55 Lake Ave North, Worcester, MA. For participants at the Bellevue site, the CTSI Bellevue research suites or designated areas in the NYU main hospital campus will be used. For Yale participants, this will be at outpatient ambulatory space adjacent to Yale-New Haven Hospital (hospital address: 20 York Street, New Haven, CT). All locations have immediate access to emergency support equipment and personnel in the event of deterioration in clinical status. Participants with severe cardiac symptoms during baseline assessment (6MWT) that would place them at potentially increased risk for adverse events with home-based exercise (e.g. hypotension (drop in systolic BP ≥15 mmHg), severe angina, unstable arrhythmia) will not undergo randomization, and the DSMB will be notified. In addition, participants with severe orthopedic complaints during 6MWT (e.g. intolerable back pain due to spinal stenosis), who need to stop walking entirely before the test is completed because of this pain, will not undergo randomization. These events will constitute a “screen failure.”

Confidentiality. As with any study that collects patient-level data, there is the potential for a breach of confidentiality due to unintentional release of research records.

For our study, all patient data will be kept strictly confidential, except when published for purposes of reporting data. In that case, the patients are never identified. All electronic data will be de-identified and transmitted and stored with secure systems that meet or exceed Federal guidelines. The Principal Investigator will maintain files with identifying information in a locked cabinet in a locked room or in password-protected files on a password-protected computer. Since IHD is a common condition and the study is being conducted in a densely populated area, it is extremely unlikely that participants could be identified from an anonymized dataset.

Financial incentives. Low-income participants (e.g. Medicaid beneficiaries) may be vulnerable to financial incentives to enroll in the research study. We will therefore delineate in the informed consent that compensation should not be the sole grounds for participation in the research project and should not cause participants to assume risks that they would not ordinarily find acceptable. We designed study compensation to be modest in order to not unduly influence participation. Simultaneously, we will also aim to ensure that participants do not incur additional costs from the study that may be burdensome. We have therefore built in transportation costs for the 3-month follow-up assessment visit. In addition, study equipment will be provided to participants at no charge, and a toll-free number will be provided to reach the research coordinator for ongoing communication as necessary. There are no anticipated out-of-pocket costs for participants using the mobile health software application. A home WiFi network is not required. Tablet devices will have cellular capability, provided by the study.

2.3.2 Known Potential Benefits

There is no definitive direct benefit to participants from study treatment or other study procedures during the course of the study. If the mHealth-CR therapy turns out to be beneficial, then participants assigned to this study arm will benefit, although this will not be known until conclusion of the study.

CONFIDENTIAL
This material is the property of the NYU School of Medicine and Langone Medical Center. Do not disclose or use except as authorized in writing by the study sponsor

3 Objectives and Purpose

The study includes objectives relate to both efficacy and engagement.

3.1 Primary Objective

The *primary efficacy objective* is to determine whether mHealth-CR, in patients age ≥65 with ischemic heart disease (IHD), improves functional capacity as assessed by 6 Minute Walk Distance (6MWD), compared to usual care.

The *primary engagement objective* is to characterize engagement of participants with mHealth-CR and elucidate barriers to participation.

3.2 Secondary Objectives

The *secondary efficacy objectives* are to evaluate whether mHealth-CR improves the endpoints of (1) goal attainment, as measured by goal attainment scaling (GAS); (2) health status (general and disease-specific); (3) basic and instrumental activities of daily living (ADLs); (4) hospital readmission; and (5) death, in participants receiving mHealth-CR versus usual care.

The *ancillary efficacy objectives* are to assess whether mHealth-CR improves the quantitative endpoints of (1) mean systolic blood pressure at 3-month follow-up visit; (2) change in frailty status; (3) attendance rate at traditional (ambulatory) CR; (4) change in PHQ-8 depression score (baseline to 3 months).

4 Study Design and Endpoints

4.1 Description of Study Design

RESILIENT is a phase II, multicenter, prospective, 3:1 open-label pragmatic randomized clinical trial (with blinded assessment of primary endpoint). Participants with IHD will be identified at the time of acute myocardial infarction (AMI), percutaneous coronary intervention (PCI), or coronary artery bypass graft (CABG), and will be randomized in a 3:1 ratio to a mobile health cardiac rehabilitation program (mHealth-CR) in addition to usual care or to usual care alone for 3 months. Randomization will be stratified by institution (NYU-main campus, NYU-Winthrop, Bellevue Hospital, and UMMHC) to ensure balance across treatment arms given between-hospital population differences. We will randomize a total of 400 participants within the NYU School of Medicine / NYU Langone Health System, Bellevue Hospital, and UMMHC, which serve a diverse patient population in the New York City and Massachusetts. We plan to enroll a target of 200 participants within the NYU School of Medicine/ NYU Langone Health System, and 150 participants within UMMHC and 50 within Bellevue Hospital.

Study enrollment has been paused indefinitely at the Yale New Haven site. This site might contribute to enrollment again in the future.

4.2 Study Endpoints

4.2.1 Primary Study Endpoints

<u>Primary Efficacy Endpoint.</u> The primary endpoint of this study is the change in the 6MWD between the baseline visit and 3-month follow-up visit. Change in 6MWD, reflective of functional capacity, is measured by the 6-minute walk test (6MWT). The 6MWT will be performed during baseline hospitalization and at the 3-month follow-up visit by a blinded research nurse or exercise therapist.

<u>Primary Engagement Endpoint</u>. Our main measure of engagement is weekly percent completion of the mHealth-CR program. Completion of mHealth-CR analyzed at weekly intervals will allow us to determine distinct engagement trajectories over the 3-month study period. Weekly engagement will be measured as the fraction of the following 11 elements completed each week: (1–7) daily entry of exercise data and RPE; (8) completed weekly phone call with exercise therapist; (9) at least one electronic communication with exercise therapist; (10) watching educational video (which will vary by week); and (11) at least one home BP measurement. Engagement will be assessed as a pseudo-continuous outcome, as the score can range from 0% (0 activities

This material is the property of the NYU School of Medicine and Langone Medical Center. Do not disclose or use except as authorized in writing by the study sponsor

completed) to 100% (all 11 activities completed). We will also explore determination of a threshold for minimally sufficient engagement that is associated with improved outcomes.

4.2.2 Secondary Study Endpoints

Protocol-defined secondary endpoints are: (1) goal attainment, as measured by goal attainment scaling (GAS); (2) change in participant reported health status: general (SF-12) and disease specific (SAQ-7); (3) change in activities of daily living (ADLs); (4) hospital readmission; and (5) death, in participants receiving mHealth-CR versus usual care.

- *Goal attainment*, defined as whether a person's individual functional goals are achieved as a result of the study intervention, will be measured using a 5-point goal attainment scale (GAS). Using the SMART goal framework,[63] GAS describes the person's expected level of goal achievement over 3 months, ranging from no change (scored as -2) to much better than expected (scored as +2). Scales are dynamically set according to a person's needs, while measurement of attainment is standardized. Goal attainment, through goal setting, is an especially important outcome in older adults who may begin an intervention with a variety of deficits (therefore necessitating individualized therapy towards realistic goals).[64]

- *Participant reported health status* is measured using the SF-12[65] (general health status) and Seattle Angina Questionnaire 7 (SAQ-7)[66] (disease-specific health status). Health status encompasses a person's functional status, symptoms, and well-being, and is increasingly recognized as an important patient-centered outcome.[67,68] Both the SF-12 and SAQ-7 have been validated and are convenient for participants (<5 minutes to administer). For the SF-12, we will analyze change from baseline to 3 months using a threshold of 5 points in SF-12 physical component score (PCS) as a clinically meaningful change.[69] For SAQ-7, we will analyze at a single time point (3 months) the number of participants who have residual angina (SAQ-7<100) vs. no angina (SAQ-7=100).[66]

- *Change in ADLs* is defined as any improvement or worsening in basic (BADLs) or instrumental (IADLs) activities of daily living over 3 months. BADLs are basic self-care behaviors: feeding, toileting, bathing, dressing, and ambulating.[70] IADLs are activities that allow a person to live independently (e.g. food preparation, medication management, transportation, shopping, managing finances, using the telephone, and housekeeping).[70] ADL preservation is repeatedly cited by older adults as an important outcome,[9] and prior research has shown that many patients with IHD experience ADL decline after hospitalization.[71]

- *Hospital readmission* is defined as an overnight stay (including observation) in any hospital within 3 months of discharge. Readmissions in older adults with IHD are common, costly, and disruptive; multiple entities within the U.S. healthcare system emphasize reducing potentially preventable readmissions.[72] Since these data are obtainable via the electronic health record (EHR), we will also ascertain readmission events at 6 months and 1 year.

- *Death* is defined by death from any cause within 3 months of enrollment. Given our sample size, we do not expect to detect a significant difference in mortality rates, but a descriptive analysis may place other endpoints in context. In addition, absolute mortality rates can assist with planning of subsequent studies. Similar to readmission, we will also ascertain death at 6 months and 1 year through the EHR.

Safety Endpoint. The safety endpoint will be assessed at the 3-month ambulatory visit and includes (1) fall-related injury (operationalized as any fall requiring acute medical care); (2) hospitalization for acute coronary syndrome; and (3) hospitalization for unstable arrhythmia. Separately, among intervention participants, study staff will monitor potential exercise-related adverse events on an ongoing basis. Points of contact will include weekly phone calls with the exercise therapist as well as electronic communication via the mHealth app which is checked daily. All adverse events will be reported directly to the Principal Investigator and to the DSMB. In order to reduce the likelihood of these events, participants will complete the baseline 6MWT in-hospital; if any adverse event occurs during 6MWT (drop in SBP ≥15 mmHg, chest pain, ventricular arrhythmia), participants will be removed (not randomized). Other exclusion criteria (severe osteoarthritis, recent joint replacement,

CONFIDENTIAL
This material is the property of the NYU School of Medicine and Langone Medical Center. Do not disclose or use except as authorized in writing by the study sponsor

moderate or severe cognitive impairment) are also intended to minimize risk. Prior studies of home-based CR have reported that adverse events are uncommon.[73,74]

4.2.3 Exploratory Endpoints

Ancillary endpoints include: (1) mean systolic BP at 3 months; (2) change in frailty status; (3) attendance at traditional CR; (4) change in depressive symptoms (PHQ-8); (5) barriers to and facilitators of participation from qualitative interviews (subsample of participants).

Ancillary endpoints *(systolic BP, change in frailty status, traditional CR attendance, change in depressive symptoms)* will be measured at the baseline visit, at-home (for intervention group), and at 3-month follow-up visit. Home BP in the intervention arm will be measured with a digital blood pressure cuff provided to participants, which synchronizes with our mHealth-CR software. Frailty will be assessed using the Fried criteria (unintentional weight loss, weakness, exhaustion, slow gait, low physical activity);[82] we will analyze the number of participants in each group who transition from "frail" to "not frail" between baseline and 3 months. For attendance at traditional CR, we will measure (for each participant) whether traditional CR attended (yes/no), date of first traditional CR visit, number of days from hospital discharge to first traditional CR visit, and total number of traditional CR sessions attended.

The System Usability Scale (SUS) will be used to measure the usability of technology and software in intervention arm participants at the 3-month follow up visit. The SUS is a 10-item questionnaire with five response options ranging from "Strongly Agree" to "Strongly Disagree". This questionnaire will be administered by the Research Coordinator during the 3-month follow-up visit and open ended feedback will be solicited from the intervention arm subjects

5 Study Enrollment and Withdrawal

5.1 Inclusion Criteria

In order to be eligible to participate in this study, an individual must meet all of the following criteria:

1. Age ≥65.
2. Currently hospitalized for AMI, PCI, or CABG ***or***
   Hospitalized for AMI, PCI or CABG within prior 4 weeks.
3. Capable of self-consent.
4. Understand and are able to perform study procedures (i.e. 6-minute walk test, use mHealth in English or Spanish).

Given logistical considerations, we expect that most participants with AMI and/or PCI will be recruited from the inpatient setting, with the baseline assessment taking place before discharge. However, given recent trends in AMI and PCI care (shorter hospital stays, including same-day discharge after PCI), we may not be able to capture all eligible participants at the time of hospitalization. In this case, the baseline study visit will take place at the first ambulatory cardiology visit (provided this occurs within 4 weeks of hospitalization). Since patients undergoing CABG have exposure to much greater physiologic stress as they recover from surgery, our expectation is that they will not be enrolled in the hospital (given inability to participate in mHealth-CR immediately after discharge), but instead will be enrolled at the first ambulatory visit (provided within 4 weeks of discharge). For patients with AMI or PCI, they can be enrolled either in the hospital or the first ambulatory visit (provided within 4 weeks of discharge).

5.2 Exclusion Criteria

An individual who meets any of the following criteria will be excluded from participation in this study:

1. Non-ambulatory.
2. Moderate or severe cognitive impairment.

CONFIDENTIAL
This material is the property of the NYU School of Medicine and Langone Medical Center. Do not disclose or use except as authorized in writing by the study sponsor

3. Unable/unwilling to consent.
4. PCI-related groin hematoma that precludes brisk walking.
5. Incarcerated.
6. Unable to use mHealth software in English or Spanish.
7. Severe osteoarthritis, or joint replacement within last 3 months.

Parkinson's disease or other progressive movement disorder.

8. Regular use of walker for ambulation.
9. Projected life expectancy <3 months.
10. Clinical judgment concerning other safety or nonadherence issues.
11. Participants admitted from long-term care facility.
12. Currently listed for heart transplant.
13. Left ventricular assist device recipient.
14. Completion of ambulatory cardiac rehabilitation program within prior 3 months.

5.3 Vulnerable Subjects

The target study population (adults ≥65 years) for this research study is unlikely to include women who are of childbearing potential, pregnant or breastfeeding. Participants who are incarcerated are not eligible to participate in this research study. Participants who become incarcerated after randomization will be discontinued from study participation and will be referred to his/ her medical provider for appropriate follow-up treatment. Exclusion of prisoners or incarcerated individuals is due to their inability to use mobile devices and limited internet access during their incarceration.

The target study population may include eligible participants with mild cognitive impairment, since this condition naturally occurs in a significant subset of the older adult population. However, we will exclude participants with overt dementia (cognitive impairment with impaired daily function) if this is mentioned as a diagnosis in the electronic medical record. This is because patients with dementia are unlikely to be able to complete the study protocol.

Impaired decision-making capacity with mild cognitive impairment is very rare.[119] If there is any question by the RC regarding impaired decision-making capacity (e.g. due to mild cognitive impairment, delirium, or psychiatric illness), then the UBACC will be administered by a member of the clinical team.

In the event of a new medical illness that has the potential to affect a participant's capacity (e.g. stroke), the UBACC will be re-administered by a member of the clinical team. If at this time the participant no longer has the capacity to consent, the RC or study team member will notify the PI and the participant will be discontinued from the study. Given the relatively short duration of the study, we anticipate that loss of capacity due to many chronic medical illnesses is unlikely.

5.4 Strategies for Recruitment and Retention

Recruitment. Inclusion and exclusion criteria will be determined through screening of the EHR.

The research coordinator will screen the list of patients and send potentially eligible patients to the Principal Investigator for review. At the NYU site, Dr. Dodson, a clinically trained cardiologist, will be responsible for reviewing the list of eligible patients and whether inclusion/exclusion criteria are met. For those deemed eligible, the research team will send an email to the attending physician (usually a cardiologist) notifying that the study team will approach the patient for participation in the research study. Dr. Marzo and Dr. George will be responsible at the NYU-Winthrop location for reviewing and confirming eligible patients identified by the NYU-Winthrop coordinator, while Dr. Kovell will be responsible at the UMMHC site for reviewing and confirming eligible patients identified by the UMMHC research coordinator(s). Dr. Chaudhry will be responsible at the Yale site for reviewing and confirming eligible patients identified by the Yale research coordinator(s).

This material is the property of the NYU School of Medicine and Langone Medical Center. Do not disclose or use except as authorized in writing by the study sponsor

For eligible patients identified at Bellevue Hospital, the Research Coordinator will send a letter introducing the trial as well as the most current IRB-approved version of the informed consent form. Eligible patients will be called for approach after the letters are sent in order to allow patients to reference the informed consent form during the phone approach. This method will account for high rates of patients at Bellevue Hospital who do not or cannot use email for receipt of informed consent after a phone approach.

Enrollment. For participants currently hospitalized for AMI, PCI, or CABG at the time of screening, participants will be approached in-person by the study team for potential enrollment in the study during a period of clinical stability in the inpatient setting (usually this is close to the time of hospital discharge). Participants who have been discharged from the hospital but are eligible due to recently diagnosed AMI, PCI, or CABG (within 4 weeks of the screening date) will receive a message through the EPIC electronic health record, introducing the participant to the study, and will have the option to "opt out" of being approached by replying to the message. If they do not opt out, they will be approached by the research coordinator for potential enrollment at the time of the next outpatient cardiology visit, provided this occurs within 4 weeks of AMI, PCI, or CABG. In the circumstance that it is not possible for the research coordinator to approach an eligible subject in-person in an inpatient setting, or within 4 weeks of discharge during an outpatient appointment, the research coordinator will call the eligible subject at the preferred phone number in their chart to introduce the study. Eligible patients will have the option of scheduling a research visit to undergo the informed consent process and to complete the baseline visit. For outpatients enrolled in the study, the baseline visit will take place in close proximity to dedicated ambulatory space at NYU Main Campus (Cardiology Faculty Group Practice), NYU Winthrop Campus (Winthrop Cardiology Associates), UMMHC (Ambulatory Care Center), or Yale-New Haven Health (Yale Cardiovascular Medicine, Outpatient Practice).

Participants will be explicitly informed that participation in the present study is voluntary, and will not affect their normal standard of care should they choose not to participate. Participants will be informed that the Principal Investigator will be available by phone or in person for any additional questions participants may have about their involvement. Comprehension of the key elements of the study procedures and risks will be tested with verbal questions of the consent form content. Participants will also be notified of all study-related activities, period of involvement, and contact information of all study personnel. Participants will also be asked to opt-in for future contact by study personnel for additional study-related activities. If the patient is willing to participate, they will sign and date the signature page of the IRB-approved informed consent form. A copy of the signed consent form will be given to the participant and the original will be placed in the research record.

Target enrollment is 400 participants between the NYU, Bellevue, UMass and Yale sites. Male and female participants of all racial backgrounds and ethnicities will be enrolled, without exclusion. We propose a recruitment target for women of at least 40% of the target enrollment participants. We aim to enroll at least 19% nonwhite patients, with at least 12% being black patients, and at least 12% patients of Hispanic ethnicity. If we find that we are not meeting targets for nonwhite and Hispanic enrollment early in our recruitment efforts, we aim to take corrective action including potential addition of other sites to increase diversity, such as Bellevue Hospital.

Retention. At the time of participant enrollment, the research coordinator will complete a participant contact sheet, which will include the participant's name, address, phone number and preferred time for study-related phone calls. The participants will also be asked to provide the contact information (phone numbers) for at least 2 friends or relatives who are aware of the participant's involvement in the study as additional contact person. These individuals will only be contacted in the event that the study participant is unresponsive to communication attempts or if the participant missed a scheduled study visit. Regular phone calls and reminders will be made with participants to maintain retention in the study.

COVID-19 Pandemic: Transportation. In the scenario that study participants encounter difficulties with transportation that would prevent them from attending the baseline and/or follow-up visits (such as inability to schedule their research visit on the same day as a standard of care visit and/or reasons relating to COVID-19 fears) all study participants will be offered free car service until the mask mandate is discontinued by New York State.

5.4.1 Use of Epic Information for Recruitment Purposes

Inclusion and exclusion criteria will be determined through screening of the EHR primarily by the study research coordinator. A daily report from Epic will be generated based on (1) diagnostic data (classifying patients as having AMI, or undergoing elective coronary revascularization) entered for patients admitted to the hospital from the time of last update (generally last 24 hours during the week except Monday, which captures last 72 hours inclusive of Friday and weekend admissions); and (2) outpatient cardiology appointments scheduled for patients diagnosed with either AMI or who recently underwent elective coronary revascularization (PCI or CABG) within the past 4 weeks. The daily report will be sent to the study research coordinator, who will screen for patients within inclusion criteria and send the list of potentially eligible patients to the Principal Investigator via email for review. Any recruitment information sent by email will utilize Send Safe email.

<u>MyChart Recruitment. If the recruitment sites' electronic health record system supports this feature,</u> participants who have been discharged from the hospital but are eligible due to recently diagnosed AMI, PCI, or CABG (within 4 weeks of the screening date) will receive a MyChart message through the EPIC electronic health record introducing the participant to the study. Participants will have the option to "opt out" of being approached by replying to the message. If they do not opt out or do not have a MyChart account, they will be approached by the research coordinator for potential enrollment at the time of the next outpatient cardiology visit, provided this occurs within 4 weeks of AMI, PCI, or CABG. DataCore will request a daily report of all participants' responses to the MyChart message and send to the research coordinator, who will screen patients within inclusion criteria for potential subject eligibility. Participants who are potentially eligible will be sent to the Principal Investigator via email for review. Any recruitment information sent by email will utilize Send Safe email.

Once contact is made, approved recruitment language will be used to communicate the reason they are being contacted and subjects will be asked if they are interested in participating in this specific study. Should the potential subjects agree, the study team will provide the subjects with information regarding the next steps for participation.

If a subject requests information regarding opting out of further recruitment for all research, subjects will be directed to contact study coordinator or have subjects contact research-contact-optout@nyumc.org or 1-855-777-7858.

Study members who will have access to the EPIC search results include the Principal Investigator and research coordinator(s). EPIC search results will include the participants' name, date of birth, age, medical record number, medical diagnoses (ICD-9 or ICD-10), lab values, diagnostic test and results (e.g. EKG, echocardiogram), procedural notes and results (e.g. PCI or CABG), and all physician inpatient and outpatient notes in order to determine subject eligibility.

Once eligible subjects have been identified and confirmed by the Principle Investigator, the study team will notify the treating physician (TP) that they have patients eligible to participate in the study via email. If there are no concerns notified by the TP, the research coordinator will approach participant in the inpatient (or outpatient) setting for potential enrollment in the study.

To ensure equitability, the research coordinator will maintain screening logs to characterize the sociodemographics of the patient population and to identify reasons for exclusion. Screening forms will include inclusion/exclusion criteria and basic demographic information (e.g. race, ethnicity, sex).

5.5 Duration of Study Participation

Participants will be asked to participate in the study for 3 months.

5.6 Total Number of Participants and Sites

Recruitment will end when approximately 400 participants are enrolled. Given an estimated 20% drop out rate, the study will enroll approximately 400 participants in order to produce 320 evaluable participants.

CONFIDENTIAL
This material is the property of the NYU School of Medicine and Langone Medical Center. Do not disclose or use except as authorized in writing by the study sponsor

5.7 Participant Withdrawal or Termination

5.7.1 Reasons for Withdrawal or Termination

Participants are free to withdraw from participation in the study at any time upon request. An investigator may terminate participation in the study if:

- Any clinical adverse event (AE), laboratory abnormality, or other medical condition or situation occurs such that continued participation in the study would not be in the best interest of the participant
- The participant meets an exclusion criterion not caused by study procedures (either newly developed or not previously recognized) that precludes further study participation.

5.7.2 Handling of Participant Withdrawals or Termination

Participants wishing to withdraw or that are terminated from the study will be asked if they may continue to be monitored for safety endpoints to capture AEs, SAEs, and unanticipated problems. Survival data on all participants who are lost to follow-up will be kept in the REDCap database (Research Electronic Data Capture), a secure, web-based Electronic Data Capture (EDC) system. Participants who withdraw or are terminated will not return for the 3-month ambulatory assessment.

Participants will be contacted by the study team personnel via phone call at least three times and/or contact with next-of-kin, if possible, before being considered as lost to follow-up. A certified letter will be sent by the study personnel to the participants as final proof of contact.

5.7.3 Premature Termination or Suspension of Study

This study may be temporarily suspended or prematurely terminated if there is sufficient reasonable cause. Written notification, documenting the reason for study suspension or termination, will be provided by the suspending or terminating party to the Principal Investigator (PI), Dr. John Dodson, and the National Institutes of Health (NIH). If the study is prematurely terminated or suspended, the PI will promptly inform the IRB and will provide the reason(s) for the termination or suspension.

Circumstances that may warrant termination or suspension include, but are not limited to:

- Determination of unexpected, significant, or unacceptable risk to participants
    - Major risks include fall-related injury requiring medical care (inpatient hospitalization) that occurs during study-directed exercise, hospitalization for acute coronary syndrome, or hospitalization for unstable arrhythmia.
- Demonstration of efficacy and engagement that would warrant stopping
- Insufficient compliance to protocol requirements
- Data that are not sufficiently complete and/or evaluable
- Determination of futility

Study may resume once concerns about safety, protocol compliance, or data quality are addressed and satisfy the applicable governing bodies.

6 Behavioral/Social Intervention

6.1 Study Behavioral or Social Intervention(s) Description

In this pragmatic trial embedded within the current standard of care, patients in both arms will receive a standardized referral to traditional ambulatory CR at hospital discharge, in accordance with professional guidelines[2] (typically started within 4 weeks at our institutions). Intervention arm participants will also receive mHealth-CR, initiated immediately, for 3 months.

This material is the property of the NYU School of Medicine and Langone Medical Center. Do not disclose or use except as authorized in writing by the study sponsor

6.1.1 Administration of Intervention

Study participants randomized to the intervention (mHealth-CR) arm during the (in-hospital) baseline visit will receive 3 components for their home activity: (1) communication with exercise therapist (in-hospital assessment/counseling followed by regular communication post-discharge), (2) mHealth-CR software, and (3) wearable activity monitoring device. These components are designed to work in concert, as described below:

Communication with exercise therapist. Study participants will meet the therapist immediately after enrollment (in-hospital). This 1-hour visit will include education on cardiac risk factor management, assessment of baseline functional status, and an introduction to mHealth-CR software. In the event that an in-person intake visit cannot be conducted with the exercise therapist, a video visit will take place instead, which will be conducted with the research coordinator physically present to facilitate interaction. We may use the Zoom Videoconferencing software to conduct the intake with the physical therapist through a password protected video phone call. A personalized exercise program will be designed that includes alternating aerobic exercise (walking, stair climbing) and low-level isometric resistance training (upper body strength exercises using elastic bands). Participants will be given the recommendation to exercise for at least 5 out of 7 days/week, with an ideal goal of 150 minutes/week of moderate intensity exercise (reflecting published U.S. guidelines);[77] however, recommendations may be tailored based on functional limitations or individual clinical scenarios (e.g. dyspnea on exertion due to heart failure). The therapist will identify potential barriers to this plan and develop mitigation strategies. Given our target population (age ≥65), the therapist will also complete a home safety protocol to engage the participant in removing fall hazards at home, and if there is additional concern they will determine eligibility for a home PT/OT evaluation after discharge. Following discharge, home exercise intensity will be rated by participants on mHealth-CR software, using relative perceived exertion (RPE) on the Borg Scale (target range 11-14). The exercise therapist will then make phone contact with participants twice in Week 1, twice in Week 2, and weekly for the remainder of the study. Exercise recommendations will be titrated during calls based on self-reported RPE and review of activity data. Participants will also receive a referral to (but not required attendance at) traditional ambulatory CR, which is the current standard of care. At NYU, Yale and UMass, the typical first visit for traditional CR occurs within 4 weeks of discharge.

mHealth-CR software. For the proposed study, we will use Moving Analytics version 2.0, with enhanced capabilities that include video conferencing, and the ability to automatically synchronize with a BP cuff (BodyTrace/Omron) and Fitbit device. To obviate barriers to portable electronic device ownership, participants will receive a tablet computer (Samsung Galaxy) with mHealth-CR software for the duration of the study. Devices will also have cellular capability (participants will not require a home Wi-Fi network). Software includes the following components: (1) participant data entry, including exercise duration and RPE; (2) “chat” function where participants can communicate questions or concerns about symptoms to the exercise therapist; (3) weekly series of educational videos, focused on secondary prevention, that include the following topics: introduction, understanding emotions, exercise guidelines, managing medications, heart healthy diet, stress reduction, smoking cessation; and (4) physiologic measurement with assessment of BP, heart rate, and physical activity. Participants will be instructed by the exercise therapist to use mHealth-CR for at least 5/7 days each week. Training will include education on BP cuff use, which will be measured by participants at home weekly. Software is available in English and Spanish. Baseline training on software use is further described in 7.2.2.

COVID-19 Pandemic: Supplement for mHealth-CR software. In the scenario that study participants in the intervention arm are unable to engage in prescribed exercise in public (outdoors or enclosed public spaces such as gyms or malls) due to fears of COVID-19 pandemic and winter weather, interventionists will provide intervention arm participants with a more robust set of activity recommendations that they are able to perform at home. Supplemental videos on a warm up, aerobic beginner, aerobic intermediate, aerobic advanced, and stretching exercises have been selected from an online exercise therapy program called “TherHab Fitness” by Dr. Patrice Hazan, accessible: https://www.hometherhabfit.com/resilient-study. Interventionists will prescribe these videos to participants who are unable to fully engage in the exercise prescription due to the aforementioned reasons. Access to the supplemental videos will be discontinued upon the lifting of the COVID-19 mask mandate by New York State.

Wearable activity monitoring device. Participants will be provided with a Fitbit wearable wrist device (Fitbit, San Francisco, CA). This is a commercially available product that measures physical activity based on number of steps per day. Activity is categorized (based on step count) as sedentary or mildly, moderately, or vigorously

CONFIDENTIAL
This material is the property of the NYU School of Medicine and Langone Medical Center. Do not disclose or use except as authorized in writing by the study sponsor

active. Data are automatically uploaded daily to Moving Analytics, and will be viewable by both the study participant and the exercise therapist. Weekly phone calls with the exercise therapist will include review of activity data including percent time spent in each category and total daily step count. Data on “wear time” (amount of day participant wears the device, versus device off participant) will also be collected.

All materials will be given to intervention participants during their baseline visit by the research study team. Estimated time for each visit is 1 hour, and 20 minutes for phone calls.

6.1.2 Procedures for Training Interventionalists and Monitoring Intervention Fidelity

To administer the study intervention, more than one exercise therapist will be hired at each site in order to enhance availability to enroll participants. We will add NYU exercise therapists as a study team member in NYU's IRB and Research Navigator portals prior to administering all study intervention. Monitoring of delivery of the study intervention in the manner specified, across therapists and study sites, is critical to maintain rigor and reproducibility, and ensure that delivery of the intervention is consistent. Accordingly, we will monitor treatment fidelity based on principles from the treatment fidelity framework outlined by NIH’s Behavior Change Consortium and described in a review by Borrelli (*J Public Health Dent* 2011; 71(s1):S52–S63).

We will take several steps to monitor fidelity across the duration of the trial. At the outset of the trial, the exercise therapists will undergo the same baseline training session in Year 1. Once the intervention has begun, a minimum of 50% of encounters with the first 50 subjects will be audiotaped and rated by the study investigators (Dr. Dodson - PI; Dr. Schoenthaler - Co-I) using a structured tool in order to monitor and remediate any protocol deviations. In the long-term, we will review a random sample of 20%. If the individual falls below the a priori performance criterion (i.e., a rating below the midpoint of the structured tool) based on ongoing review of this random sample, then returning to 50% monitoring may be warranted. Components evaluated will include (1) length of encounter, (2) number of elements covered (e.g. review of exercise activity, review of login frequency, addressing barriers to activity, and planning exercise for the next week), and (3) nonspecific factors (empathy, communication style) that may influence success of the intervention. To facilitate this process, after each session, therapists will complete an intervention form to document what happened during the session.

The intervention will follow the U.S. “Physical Activity Guidelines for Americans, 2nd Edition” (https://health.gov/paguidelines/second-edition/pdf/Physical_Activity_Guidelines_2nd_edition.pdf) which has been endorsed by the American Heart Association:

- At least 150 minutes of moderate-intensity exercise per week, or 75 minutes of vigorous intensity aerobic physical activity, or an equivalent combination. Activity should be spread throughout the week (e.g. 5 sessions of 30 minutes each).
- If 150 minutes of moderate-intensity activity is reached, participants will be encouraged to increase to 300 minutes of activity.
- Muscle strengthening activities at least 2 days per week. For purposes of the RESILIENT trial, participants will be provided with elastic resistance bands and trained on their use at the time of baseline visit.
- For those unable to achieve at least 150 minutes of moderate-intensity exercise (e.g. due to functional limitations), lower exercise targets will be adapted as endorsed by the guidelines. The principle of “move more, sit less” will be also be recommended.
- Balance training will also be incorporated into the treatment plan, as recommended by the guidelines as they pertain to older adults.

Therapists hired for the trial will have master’s level training in exercise therapy. As clinically trained professionals, they may be required to adapt the intervention based on individual study participants’ physical or sensory limitations, or specific rehabilitation needs. This concept is similar to traditional rehabilitation whereby the intervention is individualized. We will review encounters with this flexibility in mind.

6.1.3 Assessment of Subject Compliance with Study Intervention

Assessments of compliance to assigned treatment strategies will be conducted through the following:

(1) *Receipt of traditional CR*: Participants in both arms will receive standard referral to traditional (ambulatory) CR. We will summarize the uptake of this referral using attendance at initial and

This material is the property of the NYU School of Medicine and Langone Medical Center. Do not disclose or use except as authorized in writing by the study sponsor

subsequent CR program visits.

(2) *Engagement with mHealth-CR*: The mHealth-CR intervention includes a number of activities expected of participants randomized to this arm. The primary engagement endpoint is an 11-point score, tabulated weekly, indicating completion of each component of the mHealth-CR intervention: (1–7) daily entry of exercise data and rated perceived exertion (1 point for each day with total possible 7 points per week); (8) completed phone call with exercise therapist; (9) at least one electronic communication with exercise therapist; (10) watching educational video (which will vary by week); and (11) at least one home BP measurement. Engagement will be assessed as a pseudo-continuous outcome, as the score can range from 0% (0 activities completed) to 100% (all 11 activities completed).

(3) *Use of Fitbit*: Participants in the intervention arm will receive a Fitbit monitor, and data will be evaluated on a weekly basis to evaluate whether they are consistently using the device.

7 Study Procedures and Schedule

7.1 Study Procedures/Evaluations

7.1.1 Study Specific Procedures

Study procedures can be seen in Table 1.

Table 1. Schedule of Study Activities

	Baseline	Weekly	Ambulatory follow-up
**Study Week (approximate)**	**0**	**1-12**	**12**
Informed Consent	**X**		
Demographics	**X**		
Height	**X**		
Weight and Blood Pressure	**X**		**X**
6 Minute Walk Test	**X**		**X**
Short Form 12 (SF-12)	**X**		**X**
Seattle Angina Questionnaire 7 (SAQ-7)	**X**		**X**
Activities of Daily Living (ADLs)	**X**		**X**
Mini-Cog	**X**		
Goal Attainment Scaling (GAS)	**X**		**X**
Depression (PHQ-8)	**X**		**X**
Frailty Elements	**X**		**X**
Chart abstraction (comorbidities, medications, laboratory/cardiac test results)	**X**		
Randomization	**X**		
Education on cardiac risk factor management	◊		
Ascertainment of home environment and barriers to mobility	◊		
Introduction to mHealth-CR software	◊		
Personalized exercise plan	◊		
Daily passive activity monitoring (Fitbit)		◊	
Daily therapist-directed activity (walking, upper extremity resistance training)		◊	
Daily mHealth data entry		◊	
Weekly exercise therapist phone call		◊	

This material is the property of the NYU School of Medicine and Langone Medical Center. Do not disclose or use except as authorized in writing by the study sponsor

Weekly blood pressure measurement		◊	
Weekly video education		◊	
Hospital readmissions (questionnaire)			**X**
Chart abstraction (hospital readmission verification, attendance at traditional CR)		**Week 8**	**X**
		Biweekly/ Monthly	
Phone assessment of ADLs		**X**	
**X** = All participants ◊ = Intervention participants only			

- *Chart Abstraction will be performed by the research coordinator.* Elements will be entered into a secure Research Electronic Data Capture (REDCap) database created by NYU DataCore.
  - Medical history/comorbidities: hypertension, diabetes, heart failure, peripheral artery disease, atrial fibrillation, cancer, chronic lung disease, obesity, chronic kidney disease, chronic liver disease, gastrointestinal bleeding, cerebrovascular disease, dementia, osteoarthritis, tobacco use, alcohol use.
  - Outpatient medications.
  - Laboratory data: hemoglobin, platelet count, white blood cell count, sodium, potassium, BUN, creatinine, glucose, troponin (all based on admission blood draw). Peak troponin value will also be abstracted.
  - In-hospital diagnostic testing: left ventricular ejection fraction (based on transthoracic echocardiogram), coronary angiography results (number of vessels involved / % stenosis), PCI details (number and type of stents used, vessel treated).
  - Demographic data: sex, race, ethnicity, insurance status.
  - Physical examination and vital signs: height, weight, and BP.

- *Research Coordinator Assessment.* Procedures listed below will be electronically administered using standardized instruments on a secure, portable tablet computer using a REDCap portal. NYU DataCore will facilitate development of REDCap portal and secure transfer of collected data elements.
  - Additional demographic data: Marital status, education level.
  - Short Form 12 (SF-12) questionnaire.
  - Seattle Angina Questionnaire 7 (SAQ-7).
  - Activities of Daily Living (ADLs).
  - Goal Attainment Scaling. Research Coordinators will guide participants in setting 1 personal goal related to improving daily function or symptom control (e.g., walking with less dyspnea, attending family functions) to achieve over the next 3 months.
  - Mini-Cog screening instrument.
  - Depressive symptoms (PHQ-8).
  - Frailty elements. Unintentional weight loss (≥10 pounds in past 1 year), weak grip strength (measured by handheld dynamometer), exhaustion (measured by two questions from CES-D scale), and slow gait (measured in meters/second over a 15-foot walk).

- *Research Nurse Assessment.* The 6 Minute Walk Test (6MWT) is to be performed by a blinded research nurse (to measure 6MWD) following the assessment listed above. Randomization occurs after performance of 6MWT, so that patients with safety issues are excluded (as described under "potential risks", we anticipate such issues to be relatively rare). The research coordinator will alert the research nurse when the participant is enrolled and introduce the participant for 6MWT. After administering 6MWT (using standardized recommendations from the American Thoracic Society), the research nurse will complete a 6-minute Walk Test Worksheet to document what happened during the session. These forms will be periodically reviewed by the Project Coordinator.

  In the event that the research nurse is unavailable, a blinded exercise therapist unaffiliated with the study intervention activities can conduct the 6MWT during the baseline and follow-up visits. We believe this level of training is sufficient to ensure safety during the test, as 6MWT is routinely performed in cardiac patients by exercise therapists at our institution.

  Participants with severe cardiac symptoms during baseline assessment (6MWT) that would place them at potentially increased risk for adverse events with home-based exercise (e.g. hypotension (drop in systolic BP ≥15 mmHg), severe angina, unstable arrhythmia) will not undergo randomization, and the DSMB will be notified. In addition, participants with severe orthopedic complaints during 6MWT (e.g. intolerable back pain due to spinal stenosis), who need to stop walking entirely before this test is completed because of this pain, will not undergo randomization. These events will constitute a "screen failure."

CONFIDENTIAL
This material is the property of the NYU School of Medicine and Langone Medical Center. Do not disclose or use except as authorized in writing by the study sponsor

- *Randomization*. After providing informed consent and completing the baseline assessment, enrolled participants will be randomized in a 3:1 ratio to either intervention (mHealth-CR plus usual care) or control (usual care).

- *Baseline exercise therapist assessment (intervention arm only).* Participants who are randomized to the intervention arm will undergo a baseline visit by an exercise therapist hired to administer the study intervention. This will occur in the participant's room (inpatient setting) or in dedicated ambulatory research space (outpatient setting). The therapist visit is estimated to last approximately 1 hour. During this visit, participants will also be introduced to study intervention mHealth-CR components (described in 6.1.1.):
  (1) regular communication with an exercise therapist
  (2) mHealth-CR software
  (3) wearable activity monitoring device

  Participants will receive training from the exercise therapist at the baseline in-hospital (or outpatient) visit.

  Detail. Patients in the intervention group will receive an individualized exercise prescription including aerobic and strength training exercises. The CDC's Physical Activity Guidelines (PAG) goal of a minimum of 150 minutes per week of moderate intensity or 75 minutes per week of vigorous intensity exercise, including 2 days of muscle strengthening, will be used to develop the exercise prescription. The aerobic portion will be informed by the results of the six-minute walk test, while patients will also receive a general upper and lower body strength training program. The prescription will be reviewed with the patient during the therapy initial contact, either in person or remotely through Zoom video conference. As participants by definition are age ≥65, and functional impairments may be present in this population, adjustments to these goals will be made as clinically indicated (e.g. reduced initial number of target minutes in a participant with mobility impairment). Initial contact will take place within 4 weeks of hospitalization for myocardial infarction or coronary revascularization procedure. When feasible, participants will be enrolled in-hospital, but given logistical concerns (e.g. same day discharge after PCI) there will be the potential for enrollment during the first outpatient follow-up visit. The exercise prescription will be monitored on weekly phone calls to ensure compliance and will determine if adjustments need to be made (described 6.1.1).

  The exercise prescription and baseline cardiovascular fitness level will be determined from the six-minute walk test (6MWT) and initial therapy contact. Data collected during the 6MWT will include: resting and exertional vital signs, rate of perceived exertion (RPE), and distance walked (6MWD). The prescription will provide guidance on the frequency, intensity, type, and duration of an appropriate exercise session. Participants will be able to use established HR and RPE to achieve the desired exercise intensity. Participants will be provided suggestions for both aerobic and strength exercises.

  The initial exercise prescription will utilize the peak heart rate (HR), RPE, and distance walked to establish baseline goals. The primary goal for each week will focus on achieving 150 minutes of moderate exercise (typically at an RPE of 12-14/20 or HR 80-100% of peak rate) and 2 sessions of strength training using resistance bands or weights. Data collected during each week from the mHealth application and wearable device will be evaluated to determine if the individual has met the duration goal. If the individual has met the duration goal, the goal will be progressed by 15 minutes min/wk, until the individual is unable to achieve the goal or has reached 300 min/week. Intensity will monitored using the RPE and HR. Other motivators such as steps per day may also be utilized to help achieve the desired duration goal.

  Exercise Program
  Participants will be required to participate in an exercise program for study period. The exercise program will follow the FITT formula as follows:

  - **F**requency – most days of the week (5-7)

This material is the property of the NYU School of Medicine and Langone Medical Center. Do not disclose or use except as authorized in writing by the study sponsor

- Intensity – utilize HR, RPE, walk distance, and/or weekly/min goa

- **T**ype – walk, treadmill, home equipment, stairs, light resistance exercise
- **T**ime – 150-300min/week moderate intensity exercise

Participants will be encourage to include a warm-up for 5 minutes. This will be followed by exercise at the established exercise intensity. Each exercise session should end with a cool-down for 5 minutes.

Regular phone calls with study participants, after the baseline visit, will follow the same general script that includes the following elements:

1. Brief rapport building to make patient comfortable with the call
2. Check in to see how things have been progressing since last call (or initial assessment)
3. Review of prescribed exercise plain
4. Discussion of patient compliance with plan and perceived barriers/facilitators
5. Re-evaluation and modification of plan, if needed
6. Structured goals to attain until next session

- *Usual care arm*. Participants randomized to the usual care group will participate in scheduled phone calls with the research coordinator to complete the ADL assessment. ADL assessments will be conducted on a biweekly basis during the first month, and monthly during months 2 and 3.

- *3-month ambulatory assessment (all participants).* The research coordinator will schedule an ambulatory visit to take place 3 months after the baseline assessment. For NYU patients, this visit will occur at a designated research space in the main hospital campus (NYU Tisch Hospital). For participants at NYULH-Winthrop, the visit will be performed at the outpatient office of the FGP of Winthrop Cardiology Associates located at 212 Jericho Turnpike (2nd floor) Mineola, New York. For UMMHC patients, this visit will take place at the Ambulatory Care Center (1st floor) at 55 Lake Ave North, Worcester, MA. For participants at the Bellevue site, the CTSI Bellevue research suites or designated areas in the NYU main hospital campus will be used. For Yale patients, this visit will take place at ambulatory space adjacent to Yale-New Haven Hospital. The estimated time for the 3-month visit is 1 hour. Measures at 3 months will include a repeat of several baseline instruments to evaluate change over time (weight, BP, SF-12, SAQ-7, ADLs, PHQ-8). Participants will also be asked about hospital readmissions since time of last visit. 6MWT and Goal Attainment Scaling will be performed by the research nurse and/or a blinded assessor.

The Fitbit, portable tablet computer, and BP cuff will be collected. The research coordinator will also perform chart abstraction at 3-months to evaluate ambulatory CR attendance, and to verify self-report of hospital readmissions. For readmissions that occur outside the NYU, Bellevue, UMass or Yale systems, hospital records will be obtained.

7.1.2 Standard of Care Study Procedures

Both intervention and control arms will also receive a referral to (but not required attendance at) traditional ambulatory CR, which is the current standard of care. At NYU, Bellevue, UMass and Yale, the typical first visit for traditional CR occurs within 4 weeks of discharge.

7.2 Study Schedule

7.2.1 Screening

Inclusion and exclusion criteria will be determined through screening of the EHR.

Screening (1-5 days prior to enrollment, **Day -5 to Day -1**)

- A daily list is generated by EPIC based on diagnostic test results (inpatients with AMI) or the cardiac catheterization lab schedule (outpatients going for elective coronary angiography) from the time of last update (generally last 24 hours during the week except Monday, which captures last 72 hours inclusive of Friday and weekend admissions).
- A second list is generated daily by EPIC based on outpatient cardiology appointment schedules to capture participants' appointments with his/her respective cardiologist.

This material is the property of the NYU School of Medicine and Langone Medical Center. Do not disclose or use except as authorized in writing by the study sponsor

- The staff screen the list of patients and sends potentially eligible patients to the Principal Investigator for review.
- The PI (at NYU Tisch and Bellevue sites) will be responsible for reviewing the list of eligible patients and whether inclusion/exclusion criteria are met.
- For those deemed eligible, the staff will send an email to the treating clinician informing the clinician that the research coordinator will approach the patient for participation in the research study.
- If amenable to approach, RC will approach to enroll subject.
- If the research coordinator questions the capacity to self-consent, decisional impairment will be formally assessed by the using the University of California, San Diego Brief Assessment of Capacity to Consent (UBACC).

At the NYU-Winthrop location, Dr. Kevin Marzo and Barbara George (sub-investigators) will be responsible at the location for reviewing and confirming eligible patients identified by the NYU-Winthrop research coordinator. At the UMMHC site, Dr. Kovell (site PI) will be responsible at the UMMHC site for reviewing and confirming eligible patients identified by the UMMHC research coordinator. At the Yale site, Dr. Chaudhry (site PI) will be responsible at the Yale site for reviewing and confirming eligible patients identified by the Yale research coordinator.

7.2.2 Enrollment/Baseline

Enrollment/Baseline Visit (Visit 1, Day 1)

- Obtain informed consent of potential participant verified by signature on study informed consent form.
- Verify inclusion/exclusion criteria.
- Obtain demographic information, medical history, medication history, comorbidities, and laboratory/cardiac test results.
- Obtain height and weight.
- Record blood pressure.
- Administer study assessments:
  - 6-minute walk test. Standard protocol including vital signs measurement pre- and post-testing.
  - Frailty elements: Unintentional weight loss (≥10 pounds in past 1 year), weak grip strength (measured by handheld dynamometer), exhaustion (measured by two questions from CES-D scale), slow gait (measured in meters/second over a 15-foot walk), and low physical activity (measured by kilocalories per week expended).
- Conduct questionnaires:
  - SF-12
  - SAQ-7
  - ADLs
  - Mini-Cog
  - PHQ-8
  - GAS
- (Intervention Participants only):
  - Provide participants with tablet computer
  - Receive FitBit wearable activity monitor and BP cuff monitor
  - Education on cardiac risk factor management (in person or remote)
  - Ascertainment of home environment and barriers to mobility (in person or remote)
  - Introduction to the mHealth-CR software
  - Personalized exercise plan: Participants will be instructed by the exercise therapist to use mHealth-CR for at least 5/7 days each week. Training will include education on BP cuff use.
- All participants will be instructed to participate in phone calls with the research coordinator to complete ADL assessments.

This material is the property of the NYU School of Medicine and Langone Medical Center. Do not disclose or use except as authorized in writing by the study sponsor

7.2.3 Intermediate Visits

7.2.3.1 Home activities (Day 2-89)

- (All participants): Record all participants' Activities of Daily Living Assessment data once every two weeks during the first month, and then monthly during months 2 and 3 (+ 5 days post due date).
- (Intervention Participants only): Record participants' adherence to mHealth-CR program from Moving Analytics and FitBit platform
    - Daily therapist-directed activity (walking, jogging, etc.)
    - Weekly video education
    - FitBit tracking and review of steps
    - Daily mHealth data entry
- Record participants' weekly blood pressure data.
- Administer weekly therapist phone calls for counseling/activity review (+ 5 days post due date).
- Record adverse events as reported by participant or observed by investigator.

7.2.4 Final Study Visit

3-month Ambulatory Visit (Visit 2, Day 90 +30 days)

- Record adverse events as reported by participant or observed by investigator.
- Collect all study devices: tablet computer, FitBit, blood pressure cuff monitor
- All study activities to be performed during the final visit have been detailed in Table in Section 7.1.1.
- In order to minimize bias during the GAS assessment, GAS will be conducted by a blinded assessor at this visit.
- Every effort will be made to complete the 3-month visit at or as close to day 90 as possible.

7.2.5 Withdrawal/Early Termination Visit

Participants choosing to withdraw or terminate early from the study will not be required to attend the final study visit. Since they are provided with study-related equipment (tablet computer, or if applicable, FitBit and blood pressure cuff), they will be sent a prepaid box to return this equipment to the study team.

7.2.6 Unscheduled Visit

In the event of an unscheduled visit (hospitalization), the subject will undergo safety screening. Medical records will be evaluated to determine if any study-related adverse events have occurred. All adverse events reported by the subject or observed by the investigator will be documented and reported. Aside from adverse events, information gathered at these unscheduled visits will not be included in the statistical analysis.

8 Assessment of Safety

8.1 Specification of Safety Parameters

Prespecified endpoints related to efficacy and safety are detailed in Section 4.2, Study Endpoints.

8.1.1 Definition of Adverse Events (AE)

An ***adverse event*** (AE) is any symptom, sign, illness or experience that develops or worsens in severity during the course of the study. Intercurrent illnesses or injuries should be regarded as adverse events. Abnormal results of diagnostic procedures are considered to be adverse events if the abnormality:

- results in study withdrawal
- is associated with a serious adverse event

This material is the property of the NYU School of Medicine and Langone Medical Center. Do not disclose or use except as authorized in writing by the study sponsor

- is associated with clinical signs or symptoms
- leads to additional treatment or to further diagnostic tests
- is considered by the investigator to be of clinical significance

8.1.2 Definition of Serious Adverse Events (SAE)

Serious Adverse Event
Adverse events are classified as serious or non-serious. A ***serious adverse event*** is any AE that is:

- fatal
- life-threatening
- requires or prolongs hospital stay
- results in persistent or significant disability or incapacity
- a congenital anomaly or birth defect
- an important medical event

Important medical events are those that may not be immediately life threatening, but are clearly of major clinical significance. They may jeopardize the subject, and may require intervention to prevent one of the other serious outcomes noted above. For example, drug overdose or abuse, a seizure that did not result in in-patient hospitalization, or intensive treatment of bronchospasm in an emergency department would typically be considered important medical events.

All adverse events that do not meet any of the criteria for serious should be regarded as ***non-serious adverse events***.

8.1.3 Definition of Unanticipated Problems (UP)

Unanticipated Problems Involving Risk to Subjects or Others
Any incident, experience, or outcome that meets all of the following criteria:

- Unexpected in nature, severity, or frequency (i.e. not described in study-related documents such as the IRB-approved protocol or consent form, the investigators brochure, etc)
- Related or possibly related to participation in the research (i.e. possibly related means there is a reasonable possibility that the incident experience, or outcome may have been caused by the procedures involved in the research)
- Suggests that the research places subjects or others at greater risk of harm (including physical, psychological, economic, or social harm).

8.2 Classification of an Adverse Event

8.2.1 Severity of Event

For AEs not included in the protocol defined grading system, the following guidelines will be used to describe severity.

- **Mild** – Events require minimal or no treatment and do not interfere with the participant's daily activities.
- **Moderate** – Events result in a low level of inconvenience or concern with the therapeutic measures. Moderate events may cause some interference with functioning.
- **Severe** – Events interrupt a participant's usual daily activity and may require systemic drug therapy or other treatment. Severe events are usually potentially life-threatening or incapacitating.

CONFIDENTIAL
This material is the property of the NYU School of Medicine and Langone Medical Center. Do not disclose or use except as authorized in writing by the study sponsor

8.2.2 Relationship to Study Intervention

For all collected AEs, the clinician on the study team will determine the AE's causality based on temporal relationship and his/her clinical judgment. The degree of certainty about causality will be graded using the categories below.

- **Definitely Related** – There is clear evidence to suggest a causal relationship, and other possible contributing factors can be ruled out. The clinical event, including an abnormal laboratory test result, occurs in a plausible time relationship to drug administration and cannot be explained by concurrent disease or other drugs or chemicals. The response to withdrawal of the drug (dechallenge) should be clinically plausible. The event must be pharmacologically or phenomenologically definitive, with use of a satisfactory rechallenge procedure if necessary.
- **Probably Related** – There is evidence to suggest a causal relationship, and the influence of other factors is unlikely. The clinical event, including an abnormal laboratory test result, occurs within a reasonable time after administration of the drug, is unlikely to be attributed to concurrent disease or other drugs or chemicals, and follows a clinically reasonable response on withdrawal (dechallenge). Rechallenge information is not required to fulfill this definition.
- **Possibly Related** – There is some evidence to suggest a causal relationship (e.g., the event occurred within a reasonable time after administration of the trial medication). However, other factors may have contributed to the event (e.g., the participant's clinical condition, other concomitant events). Although an AE may rate only as "possibly related" soon after discovery, it can be flagged as requiring more information and later be upgraded to "probably related" or "definitely related," as appropriate.
- **Unlikely to be related** – A clinical event, including an abnormal laboratory test result, whose temporal relationship to drug administration makes a causal relationship improbable (e.g., the event did not occur within a reasonable time after administration of the trial medication) and in which other drugs or chemicals or underlying disease provides plausible explanations (e.g., the participant's clinical condition, other concomitant treatments).
- **Not Related** – The AE is completely independent of study drug administration, and/or evidence exists that the event is definitely related to another etiology. There must be an alternative, definitive etiology documented by the clinician.

8.2.3 Expectedness

The Principle Investigators and co-investigators will be responsible for determining whether an AE is expected or unexpected. An AE will be considered unexpected if the nature, severity, or frequency of the event is not consistent with the risk information previously described for the study intervention.

8.3 Time Period and Frequency for Event Assessment and Follow-Up

The occurrence of an AE or SAE may come to the attention of study personnel during study visits and interviews of a study participant presenting for medical care, or upon review by a study monitor. All AEs including local and systemic reactions not meeting the criteria for SAEs will be captured on the appropriate CRF. Information to be collected includes event description, time of onset, clinician's assessment of severity, relationship to study procedures (assessed only by those with the training and authority to make a diagnosis), and time of resolution/stabilization of the event. All AEs occurring during the active 3 month period of the study will be documented appropriately regardless of relationship. All AEs will be followed to adequate resolution.

Any medical condition that is present at the time that the participant is screened will be considered as baseline and not reported as an AE. However, if the study participant's condition deteriorates at any time during the study, it will be recorded as an AE. UPs will be recorded in the data collection system throughout the study.

CONFIDENTIAL
This material is the property of the NYU School of Medicine and Langone Medical Center. Do not disclose or use except as authorized in writing by the study sponsor

Changes in the severity of an AE will be documented to allow an assessment of the duration of the event at each level of severity to be performed. AEs characterized as intermittent require documentation of onset and duration of each episode.

The PI will record all reportable events with start dates occurring any time after informed consent is obtained until the last day of study participation. At each study visit, the investigator will inquire about the occurrence of AE/SAEs since the last visit. Related events will be followed for outcome information until resolution or stabilization.

8.4 Reporting Procedures – Notifying the IRB

8.4.1 Adverse Event Reporting

In the unlikely event that serious and unanticipated and related adverse events or unanticipated problems involving risks to participants or others occur, adverse events will be reported to the NYU SoM IRB, the NIH Program Officer, and the study's DSMB as appropriate (i.e. relationship to study intervention, severity). The Principal Investigators will inform fellow investigators and study personnel, via email, of all adverse events that occur during the conduct of this research project as they are reviewed. The National Institutes of Health will be informed of serious adverse events such as compromise of patient privacy with 7 days of the event becoming known to the principal investigators. Adverse events and serious adverse events occurring during the active period of the study (3 months after baseline visit) will be monitored and reported to DSMB for all study subjects.

8.4.2 Serious Adverse Event Reporting

Serious adverse events will be reported to the NYU SOM IRB, the NIH Program Officer and the study's DSMB as appropriate (i.e. relationship to study intervention, severity). For unanticipated serious adverse events thought to be possibly related to study procedures, the investigator will complete and submit an SAE Form to the NIA Program Officer and to the DSMB Chair or designated DSMB member within 48 hours of study team being notified of the event. A separate report would also be submitted to the NYU SOM IRB in compliance to their Reportable New Information policy as soon as possible, but in all cases within 5 working days. Copies of relevant anonymized medical records will be forwarded to the DSMB as soon as available.

The investigator must complete and submit a follow-up SAE form and additional medical records when additional relevant information becomes available. The investigator will follow all reportable events until resolution or stabilization.

In the event of participant deaths, in compliance with NIA requirements for investigators conducting human intervention studies, all deaths would require expedited reporting (approximately 24 hours after the study team is notified of the death). This report will be submitted to the NIA Program Officer and to the DSMB Chair or designated DSMB member.

Any serious adverse event that is ongoing when a patient completes his/her participation in the trial must be followed by the site investigator until any of the following occurs:

- The event resolves or stabilizes.
- The event returns to baseline condition or value (if a baseline value is available).

All adverse events that do not meet any of the criteria for serious should be regarded as non-serious adverse events.

8.4.3 Unanticipated Problem Reporting

Incidents or events that meet the OHRP criteria for UPs require the creation and completion of an UP report form. The UP report will include the following information:

- Protocol identifying information: protocol title and number, PI's name, and the IRB project number;

This material is the property of the NYU School of Medicine and Langone Medical Center. Do not disclose or use except as authorized in writing by the study sponsor

- A detailed description of the event, incident, experience, or outcome;

CONFIDENTIAL
This material is the property of the NYU School of Medicine and Langone Medical Center. Do not disclose or use except as authorized in writing by the study sponsor

- An explanation of the basis for determining that the event, incident, experience, or outcome represents an UP;
- A description of any changes to the protocol or other corrective actions that have been taken or are proposed in response to the UP.

To satisfy the requirement for prompt reporting, UPs will be reported using the following timeline:

- UPs that are SAEs will be reported to the NIH within 48 hours of the investigator becoming aware of the event, and to the IRB within 5 working days.
- Any other UP involving risk to study participants or others will be reported to the NIH within 72 hours of the investigator becoming aware of the problem and to the IRB within 5 working days.
- All UPs should be reported to appropriate institutional officials (as required by an institution's written reporting procedures), the supporting agency head (or designee), and OHRP within two weeks of the IR's receipt of the report of the problem from the investigator.

8.4.4 Reporting of Pregnancy

The target study population (adults ≥65 years) for this research study does not include women who are of childbearing potential, pregnant or breastfeeding.

8.5 Reporting Procedures – Notifying the Study Sponsor

The study clinician will complete a SAE Form within the following timelines:

- In the unlikely event that serious and unanticipated and related adverse events or unanticipated problems involving risks to participants or others occur, they will be reported in writing within 72 hours to the IRB and the National Institutes of Health.
- Other SAEs regardless of relationship will be submitted to the NIH within 48 hours of site awareness.

All SAEs will be followed until satisfactory resolution or until the site investigator deems the event to be chronic or the adherence to be stable. Other supporting documentation of the event may be requested by the sponsor and should be provided as soon as possible.

As a follow-up to the initial report, within the following 48 hours of awareness of the event, the investigator shall provide further information, as applicable, on the unanticipated event or the unanticipated problem in the form of a written narrative. This should include a copy of the completed Unanticipated Problem form, and any other diagnostic information that will assist the understanding of the event. Significant new information on ongoing unanticipated adverse effects shall be provided promptly to the study sponsor.

8.6 Reporting Procedures – Participating Investigators

The Principal Investigators will inform fellow investigators and study personnel, via email, of all adverse events that occur during the conduct of this research project as they are reviewed.

8.7 Study Halting Rules

Administration of study will be halted when the DSMB deems appropriate. The DSMB will review AE listing reports and convene an ad hoc meeting by teleconference or in writing as soon as possible. The DSMB will provide recommendations for proceeding with the study to the NIH.

CONFIDENTIAL
This material is the property of the NYU School of Medicine and Langone Medical Center. Do not disclose or use except as authorized in writing by the study sponsor

Other risks that may warrant the halt of the study are indicated in Section 5.7.3, Premature Termination or Suspension of Study.

8.8 Safety Oversight

It is the responsibility of the Principal Investigator to oversee the safety of the study at his/her site. This safety monitoring will include careful assessment and appropriate reporting of adverse events as noted above, as well as the construction and implementation of a site data and safety-monitoring plan. Medical monitoring will include a regular assessment of the number and type of serious adverse events.

Safety oversight will be under the direction of a DSMB composed of individuals with the appropriate expertise, including 3 physicians (at least 1 cardiologist and 1 geriatrician) and 1 biostatistician. The DSMB will meet in-person once during Year 1 (first DSMB meeting) and will meet at least twice per year via teleconference to review study progress, data quality and participant safety. The DSMB will operate under the rules of an approved charter that will be written and reviewed at the organizational meeting of the DSMB. At this time, each data element that the DSMB needs to assess will be clearly defined. The DSMB will provide its input to NIH staff.

8.8.1 Events/Data to be Reviewed by DSMB

The DSMB will review the following:

Safety Monitoring Events.

- Listing of all Adverse Event (AE) by hospital and participants over time – expected or unexpected, and related or non-related to treatment
- Listing of all Serious Adverse Event (SAE) by hospital and participants over time - expected or unexpected, and related or non-related to treatment
- Listing of Unanticipated Problems (UP) by hospital and participants over time - expected or unexpected, and related or non-related to treatment
- Listings of all currently unresolved AEs
- Listings of all currently unresolved SAEs

Data Management.

- Percentage of participants accrued via an accrual report, by hospital and month of enrollment
- Percentage of participants at various stages of the study protocol (e.g., screening, enrollment, follow-up, completed)
- Percentage of missed visits, by treatment group
- Number of participants who completed follow-up visits to date, by treatment group
- Number of screening failures by treatment group over time
- Number of participants who terminated the study early by treatment group over time

8.8.2 Pre-Specified Stopping Rules

Recommendations for stopping of the trial due to evidence of benefit or harm will be based on statistical guidelines specified before the start of the trial and other internal and external factors considered relevant by the Committee members.

Circumstances that may warrant termination or suspension include, but are not limited to:

- Determination of unexpected, significant, or unacceptable risk to participants
  - Major risks include fall-related injury requiring medical care (inpatient hospitalization) that occurs during study-directed exercise, hospitalization for acute coronary syndrome, or hospitalization for unstable arrhythmia.

This material is the property of the NYU School of Medicine and Langone Medical Center. Do not disclose or use except as authorized in writing by the study sponsor

- Demonstration of efficacy and engagement that would warrant stopping
- Insufficient compliance to protocol requirements
- Data that are not sufficiently complete and/or evaluable
- Determination of futility

Recommendations for stopping of the trial due to evidence of futility will not be based on pre-specified criteria, but rather will be based on a consensus that a combination of recruitment shortfall and/or other problems with adherence to the study protocol have created a situation in which the likelihood of successful assessment of the study aims is very low and not amenable to remediation through revision of the study protocol.

A decision to override the pre-specified stopping points must be based on a sound scientific analysis derived from internal or external data (e.g., ethical, statistical, practical, or financial) that provides a clear rationale for study continuation and must be unanimously approved by all DSMB voting members. After appropriate discussion, the Chairperson will summarize and encourage a consensus opinion.

8.8.3 Dissemination of Decision by DSMB

The Chairman of the DSMB will send a communication by email containing its recommendations to the Principal Investigator (Dr. Dodson) within 14 days after each meeting. The Principal Investigator will then communicate the decision from the DSMB to the UMMHC and Yale Principal Investigator within 24 hours.

9 Clinical Monitoring

Clinical site monitoring is conducted to ensure that the rights and well-being of human subjects are protected, that the reported trial data are accurate, complete, and verifiable, and that the conduct of the trial is in compliance with the currently approved protocol/amendment(s), with GCP, and with applicable regulatory requirement(s).

- The contact Principal Investigator (PI), Dr. Dodson, will be responsible for ensuring all participants' safety through targeted random review on a monthly basis throughout the study period.
- Independent audits will be conducted by the DSMB to ensure monitoring practices are performed consistently across all participating sites.

10 Statistical Considerations

10.1 Statistical and Analytical Plans (SAP)

The statistical plan is delineated for the primary, secondary, and exploratory outcomes below.

10.2 Statistical Hypotheses

Primary Endpoints

Efficacy: 6 minute walk distance (6MWD)
Hypothesis: Degree of improvement in 6MWD will be greater in participants assigned to mHealth-CR than in those assigned to usual care.

Engagement: Mobile health (mHealth) use

This material is the property of the NYU School of Medicine and Langone Medical Center. Do not disclose or use except as authorized in writing by the study sponsor

Hypotheses: (1) Among participants offered mHealth-CR, there will be distinct trajectories of engagement (e.g., sustained engagement, disengagement, reengagement, deteriorating engagement); and (2) there will be significant differences in participant characteristics among these groups.

Secondary Endpoints

1. Goal attainment (measured by Goal Attainment Scaling)
2. Health status (measured by SF-12 and SAQ)
3. Activities of daily living (ADLs)
4. Hospital readmission
5. Death

Hypotheses: Goal attainment scaling, health status, and ADLs will improve in participants who are offered mHealth-CR compared with usual care. Hospital readmission and death will be lower in participants who are offered mHealth-CR compared with usual care.

10.3 Analysis Datasets

All randomized participants will be analyzed according to the treatment group to which they were randomized, regardless of subsequent crossover. The intent-to-treat (ITT) population will include all subjects who receive a randomized treatment assignment, except for those with a documented violation of trial eligibility criteria.

10.4 Description of Statistical Methods

10.4.1 General Approach

Statistical comparisons will be performed using two-sided significance tests and two-sided confidence intervals. We will begin all analyses with descriptive summary statistics and graphical displays of all variables, with attention to assessing balance in these characteristics by intervention group, and with assessment of the distribution of variables, relevant to the choice of statistical tests.

10.4.2 Analysis of the Primary Efficacy Endpoint(s)

6MWD
We will assess the difference in 6MWD by calculating difference scores for each participant and comparing the intervention and usual care groups with an independent group t-tests allowing for unequal variances. We will also regress 3-month 6MWD on a binary indicator of treatment group, with adjustment for baseline 6MWD and the stratification factor (enrollment site). While randomization should obviate the need for additional adjustment, we will explore whether adjustment for participant-level characteristics (e.g., demographic factors or referral to ambulatory CR) is necessary, using the change-in-estimate criterion. We realize that engagement with the mHealth-CR program may also affect attendance and engagement with structured ambulatory CR programs. We intend to explore this as a mediator of the effect of assignment to the intervention arm. We will use structural equation models to estimate the direct and indirect effects of mHealth, where the direct effect is that of mHealth-CR on the 6MWD, and the indirect effect is mediated through attendance at structured ambulatory CR.

Engagement
We hypothesize that (1) among participants offered mHealth-CR, there will be distinct trajectories of engagement (e.g., sustained engagement, disengagement, reengagement, deteriorating engagement); and (2) there will be significant differences in participant characteristics among these groups. While our aim is largely exploratory (given the paucity of data on mHealth-CR engagement), we will test whether the following characteristics are significant: age (≥80 years), sex, race/ethnicity, comorbidity burden (≥2 chronic medical conditions), frailty, social support (based on living alone), cognitive impairment (based on Mini-Cog), and depressive symptoms (based on PHQ-8). We selected these factors based on literature related to engagement in other technologies. We will conduct latent class analysis to identify profiles of engagement and explore whether these factors indicate membership in a class; these models use maximum likelihood estimation,

This material is the property of the NYU School of Medicine and Langone Medical Center. Do not disclose or use except as authorized in writing by the study sponsor

implemented with the iterative EM algorithm, to identify a latent class solution for the set of indicators. We will evaluate model fit using the G^2 statistic and compare models with the likelihood-difference test for nested models and the Akaike and Bayesian information criteria (AIC, BIC) for non-nested models. While we have identified four potential classes a priori, we will use the parametric bootstrap likelihood ratio test to select the optimal number of classes supported by the data in conjunction with the AIC and BIC. The output of the model will be a set of "item-response" probabilities giving the likelihood of a particular characteristic within each latent class, and a set of posterior predicted probabilities of latent class membership; uncertainty in predicting class membership will be summarized using the odds of correct classification (OCC) diagnostic tool.

10.4.3 Analysis of the Secondary Endpoint(s)

Analysis of secondary efficacy endpoints will proceed in a similar fashion to the primary endpoints:

Goal attainment will be assessed at baseline and 3 months. At baseline, participants will set 1 personal goal related to improving daily function or symptom control (e.g., walking with less dyspnea, attending family functions). At 3 months, goal attainment will be rated using a 5-point GAS, scored as follows: no change from baseline (-2); less than expected (-1), expected (0), better than expected (+1), and much better than expected (+2). To preserve the ordinal nature of the data, we will calculate a median GAS score (-2 to +2) for each participant, and then compare the treatment and control groups using a Wilcoxon-Mann-Whitney rank-sum test.[89] Secondly, we will transform each participant's GAS score into a single aggregated T-score using the standardized formula: $T = 50 + ([10\sum(w_i x_i)] / [(1-\rho)(\sum w_i)^2 + \rho(\sum w_i)^2]^{1/2})$, where w_i = weight assigned to the goal; x_i = the attained score for the goal (-2 to +2); and ρ = the expected correlation of the goal scales, commonly estimated to be 0.3. For this study, all weights will be assigned a value of 1 (i.e., all goals are weighted equally). A T-score of 50 indicates that on average the expected level of goal attainment was achieved.[12] We will then compare mean T-scores for treatment and control groups at 3 months using a two-sample t-test. We will also compare the percentage of participants in the treatment and control groups who met their expected level of goal attainment on at least 1 goal using a chi-square test.

Health status at 3 months will be assessed using linear regression with adjustment for baseline levels and a binary treatment indicator; if necessary, health status scores will be log-transformed to improve the approximation to normality. Activities of daily living (BADLs and IADLs) will be assessed using longitudinal models for interval scores, with indicators to incorporate time, a binary indicator of treatment group, and patient-level random effects to accommodate repeated assessments within individuals; if interval scores are not approximately normally distributed, suitable transformations will be sought. Hospital readmission and death will be evaluated using Kaplan-Meier estimates and tested with logrank statistics, as well as investigated using Cox proportional hazard models with adjustment for confounders if necessary. As with the models for the primary outcome, in each model described we will assess the need for adjustment for confounders using the change-in-estimate criterion.

10.4.4 Safety Analyses

The safety endpoint will be assessed at the 3-month ambulatory visit and includes:

(1) fall-related injury (operationalized as any fall requiring acute medical care)
(2) hospitalization for acute coronary syndrome
(3) hospitalization for unstable arrhythmia

Separately, among intervention participants, study staff will monitor potential exercise-related adverse events on an ongoing basis and report any related AEs to the Principal Investigator. Points of contact will include weekly phone calls with the exercise therapist as well as electronic communication via the mHealth app which is checked daily.

In order to reduce the likelihood of these events, participants will complete the baseline 6MWT in-hospital; if any adverse event occurs during 6MWT (drop in SBP ≥15 mmHg, chest pain, ventricular arrhythmia),

CONFIDENTIAL
This material is the property of the NYU School of Medicine and Langone Medical Center. Do not disclose or use except as authorized in writing by the study sponsor

participants will be removed (not randomized). Other exclusion criteria (severe osteoarthritis, recent joint replacement, moderate or severe cognitive impairment) are also intended to minimize risk.

All adverse events will be reported directly to the Principal Investigator and to the DSMB, as outlined in Section 8, Assessment of Safety. Each adverse event will be counted only once for a given participant. Events will be described by start date, stop date, severity, relationship, outcome, and duration. If it is determined that the adverse event precludes further participation in the study among patients in the intervention arm, the study intervention will be discontinued, but participant data will be analyzed using the intention-to-treat principle.

For analysis of the safety endpoint between intervention and control arms, a two-sided significance test with two-sided confidence intervals will be performed (as with the efficacy endpoints).

10.4.5 Adherence and Retention Analyses

Adherence (termed engagement in our study, which is more reflective of patients' use of mHealth interventions) is a prespecified endpoint in our trial. To measure engagement with the assigned treatment strategy we will perform the following analyses:

(1) Engagement with mHealth-CR: The mHealth-CR intervention includes a number of activities expected of participants randomized to this arm. The primary engagement endpoint is an 11-point score, tabulated weekly, indicating completion of each component of the mHealth-CR intervention: (1–7) daily entry of exercise data and rated perceived exertion (1 point for each day with total possible 7 points per week); (8) completed phone call with exercise therapist; (9) at least one electronic communication with exercise therapist; (10) watching educational video (which will vary by week); and (11) at least one home BP measurement. Engagement will be assessed as a pseudo-continuous outcome, as the score can range from 0% (0 activities completed) to 100% (all 11 activities completed).

Participant engagement is a primary endpoint of the study. We will conduct latent class analysis to identify profiles of engagement and explore whether these factors indicate membership in a class; these models use maximum likelihood estimation, implemented with the iterative EM algorithm, to identify a latent class solution for the set of indicators. We will evaluate model fit using the G2 statistic and compare models with the likelihood-difference test for nested models and the Akaike and Bayesian information criteria (AIC, BIC) for non-nested models. While we have identified four potential classes a priori, we will use the parametric bootstrap likelihood ratio test to select the optimal number of classes supported by the data in conjunction with the AIC and BIC. The output of the model will be a set of "item-response" probabilities giving the likelihood of a particular characteristic within each latent class, and a set of posterior predicted probabilities of latent class membership; uncertainty in predicting class membership will be summarized using the odds of correct classification (OCC) diagnostic tool.

To elucidate barriers to participation, we will use procedures that focus on developing coding protocols to highlight issues, problems, and potential recommendations. The goals of these analyses are to identify key barriers and facilitators, as well as any emerging themes related to the use of mHealth-CR to improve functional recovery and other patient-centered outcomes. Focus groups and interviews will employ semi-structured interview guides. Two independent viewers will record, transcribe, and code sessions. Coding will involve breaking responses into discrete units (e.g., sentences, phrases) and categorizing concepts into thematic groups. Discrepancies in coding will be resolved by reaching consensus among two investigators.

(2) Receipt of traditional CR: Participants in both arms will receive standard referral to traditional (ambulatory) CR. We will summarize the uptake of this referral using attendance at initial and subsequent CR program visits.

CONFIDENTIAL
This material is the property of the NYU School of Medicine and Langone Medical Center. Do not disclose or use except as authorized in writing by the study sponsor

10.4.6 Baseline Descriptive Statistics

An enrollment summary will be provided giving the total number of enrolled patients by participating hospital, with both monthly and cumulative totals provided on an ongoing basis. Baseline demographic and clinical characteristics will be tabulated by hospital for the ITT population.

10.4.7 Planned Interim Analysis

There are no formal interim statistical analyses planned. The intervention (cardiac rehabilitation) has known benefits; the novelty of our trial is the delivery system for the intervention, which we anticipate will only have the (relatively low) risk of adverse events associated with exercise. In addition, the full sample size is needed to provide necessary information to achieve the study objectives.

10.4.7.1 Safety Review

Termination of the study intervention will occur when risks are posed to participants. Safety endpoints (designed to incorporate major risks) include fall-related injury requiring medical care (inpatient hospitalization) that occurs during study-directed exercise, hospitalization for acute coronary syndrome, or hospitalization for unstable arrhythmia.

Safety review will be performed independently by the DSMB. All adverse events will be reported to the DSMB and reviewed by the Principal Investigator on an ongoing basis. The procedure and scope of the safety monitoring parameters will be determined by the DSMB.

10.4.7.2 Efficacy Review

Not applicable as no interim analyses planned.

10.4.8 Additional Sub-Group Analyses

We will perform exploratory analyses stratified by sex race, and ethnicity, in order to investigate these characteristics as mediators of our endpoints.

10.4.9 Multiple Comparison/Multiplicity

Not applicable as there is one primary efficacy endpoint.

10.4.10 Tabulation of Individual Response Data

Individual participant data will be listed by measure and time point.

10.4.11 Exploratory Analyses

Exploratory analyses for ancillary endpoints (not primary or secondary endpoints), with rationale, are as follows:

-Mean systolic blood pressure (BP) at 3-month follow-up visit. Adequately controlled BP is an important component of secondary prevention for patients with IHD, and may be influenced by participation in mHealth-CR (mediated by direct effects of exercise, as well as by feedback from weekly home BP measurement which may influence medication management and adherence). Rather than determining a threshold for optimal BP control, which remains an area of debate, we will compare mean systolic BP in the intervention versus usual care groups at 3 months.

-Frailty. Frailty is a syndrome of increased physiologic vulnerability to stressors. In older adults with IHD,

CONFIDENTIAL
This material is the property of the NYU School of Medicine and Langone Medical Center. Do not disclose or use except as authorized in writing by the study sponsor

frailty is common and associated with a range of adverse outcomes. We will measure frailty at baseline and 3 months using the criteria described by Fried et al: unintentional weight loss (≥10 pounds), weak grip strength (measured by handheld dynamometer), exhaustion, slow gait, and low physical activity. Participants meeting 3/5 of these criteria will be classified as frail. Since mHealth-CR may mechanistically modulate the frailty phenotype by building muscle mass and promoting physical activity, we will analyze the number of participants in each group that transition from “frail” to “not frail” between baseline and 3 months.

-Attendance at traditional (ambulatory) CR. We will ask participants at 3 months whether they have attended ambulatory CR. Based on the study protocol, all participants will receive a referral to ambulatory CR at time of discharge. We will therefore examine the rates of attendance (rather than rates of referral) between participants in the intervention versus usual care arms. Since participants may not recall the number of sessions attended, we will count number of sessions via electronic health record (EHR) review.

-Change in depressive symptoms (PHQ-8) (baseline to 3 months). Depression is common among IHD patients and associated with poor outcomes. While baseline depressive symptoms may determine engagement (to be evaluated in Aim 5), participation in mHealth-CR may also influence subsequent depressive symptoms. The PHQ-8 is a commonly used screen for depressive symptoms that is publicly available, easy to administer, and has been used to assess responsiveness to therapy. For our analysis, we will analyze mean change in PHQ-8 score from baseline to 3 months (intervention versus usual care

A PHQ-8 score of 10 or greater is suggestive of major depressive disorder (MDD), while a score of 20 or greater is suggestive of severe major depressive disorder. Study participants with a score of 10 or greater will be asked if they are already in treatment for depressive symptoms (defined as prescribed antidepressant medication or in active counseling.) If the subject is not in treatment, we will ask for their consent to notify their primary care provider or cardiologist about the result of the screening questionnaire.

-Barriers to and facilitators of participation from qualitative interviews (subsample of participants). The ancillary endpoint of barriers to, and facilitators of, participation will be collected through focus groups, which will be conducted with a purposive sampling of participants (8-10 per category) across levels of engagement (e.g., sustained engagement, disengagement, reengagement, deteriorating engagement).83 To optimize recruitment success, we will identify members of each group through interim data analyses. Participants will be eligible after the 3 month visit is complete. Depending on engagement patterns, questions will include topics such as reasons for change in mHealth use, features that influenced engagement, perceived satisfaction, ease of use, and recommended changes to sustain engagement. Each group will last one hour, and will be audiotaped. Dr. Schoenthaler (Co-I) will conduct all focus groups using a semi-structured moderator’s guide that will include structured prompts to elicit themes. Interviews will be transcribed verbatim and coded using Atlas.ti software.

10.5 Sample Size

We will randomize 400 participants using a 3:1 allocation, resulting in 300 participants in the intervention group and 100 participants in the usual care group. While we will aim to minimize participant dropout, we include adjustment for up to 20% dropout in each arm, resulting in about 320 participants with evaluable endpoints (240 intervention, 80 usual care). We wish to be able to detect a clinically meaningful difference between groups in our primary efficacy endpoint, change in 6MWD from baseline to 3 months. A recent meta-analysis[57] found an average difference in 6MWD of about 60 meters before and after traditional CR. Perera et al. determined this degree of change in 6MWD to be clinically meaningful based on its relationship to other health status measures.[98] Minneboo et al., however, also estimated an improvement in 6MWD in a control group of approximately 35 meters.[59] We therefore require adequate power to detect a difference between groups of less than 25 meters in change in 6MWD (i.e., the difference between a 60-meter improvement in the intervention group and a 35-meter improvement in the usual care group); this is the same amount estimated by Gremeaux et al. as a minimal clinically important difference in patients with IHD.[99] Assuming a conservative standard deviation estimate of 60 meters,[57] 320 participants provide approximately 90% power to detect a difference between groups of 25 meters, using a two-sided, 0.05-level test; there is 80% power to detect a difference

This material is the property of the NYU School of Medicine and Langone Medical Center. Do not disclose or use except as authorized in writing by the study sponsor

between groups as small as 22 meters. This sample size also provides at least 80% power to detect reasonable effect sizes in secondary endpoints.

While the sample size was determined to provide sufficient power to detect a clinically meaningful difference in the primary outcome of 6MWD, it enables comparisons of secondary efficacy endpoints as well. The table below shows clinically meaningful effect sizes for these outcomes, and gives the power to detect these effect sizes

CONFIDENTIAL
This material is the property of the NYU School of Medicine and Langone Medical Center. Do not disclose or use except as authorized in writing by the study sponsor

with our expected sample of 320 patients, using two-sided, 0.05-level tests; we will not implement multiple comparisons correction for these secondary outcomes. Baseline event rates and standard deviations are estimated form the literature.[115–118] For example, we have 90% power to detect a difference between groups of 5 units in the SF-12 score.

All comparisons between the randomized groups in this trial will be performed according to the principle of "intention-to-treat"; that is, participants will be analyzed according to the treatment group to which they were randomized, regardless of subsequent crossover. The intent-to-treat (ITT) population will include all subjects who receive a randomized treatment assignment, except for those with a documented violation of trial eligibility criteria.

The table below shows clinically meaningful effect sizes for secondary efficacy outcomes, and gives the power to detect these effect sizes with our expected sample of 320 patients, using two-sided, 0.05-level tests; we will not implement multiple comparisons correction for these secondary outcomes.

Outcome	Endpoint definition	Effect size	Estimated s.d.	Power
Goal attainment scaling	GAS score	5	15	73%
Health status	SF-12	5	12	90%
Health status	Residual angina on SAQ	15-20%	n/a	67-93%
ADLs	Standardized ADL score	15-20%	1	64-87%
Hospital readmission	Admission by 3 months	15%	n/a	91%
Death	Death by 3 months	10%	n/a	82%

10.6 Measures to Minimize Bias

10.6.1 Enrollment/Randomization/Masking Procedures

Randomization. After providing informed consent and completing the baseline assessment, enrolled participants will be randomized by REDCap in a 3:1 ratio to either intervention (mHealth-CR) or control (usual care. The rationale for 3:1 randomization is to efficiently address both the primary comparison of mHealth-CR and usual care with respect to the 6MWD and to allow an adequate sample size to estimate engagement trajectories. The proposed 3:1 randomization enables a 50% increase in the sample size, while also providing excellent power for the primary hypothesis test. Randomization will be stratified by hospital (NYU-main campus, NYU Winthrop, Bellevue, UMMHC, Yale New Haven) to ensure balance across treatment arms given between-hospital population differences. The randomization code will be created by an independent biostatistician, and given to a DataCore staff member, with a backup copy retained by the independent biostatistician.

Missing data. In general, all available data will be included in data listings and tabulations. Population denominators will be displayed in column headers. Individual denominators will be displayed for each summary such that the amount of missing values will be evident. Patients who withdraw from the study or are lost to follow-up will still be included in the denominators for any proportions where data are available.

Masking. To minimize bias and ensure safety, 6MWT will be performed by a blinded research nurse or blinded exercise therapist unaffiliated with the study intervention. The 6MWT will be performed during baseline hospitalization and at the 3-month follow-up visit. Designating a blinded research nurse serves two purposes: first, since the research coordinator will know the participant's study assignment at 3 month follow-up, a blinded research nurse will reduce the potential for measurement bias (e.g. no differential encouragement in the intervention arm). Second, having a nurse perform the assessment ensures optimal monitoring of potential safety issues (e.g. unsteady gait, lightheadedness) that may warrant cessation of the test. The research coordinator will alert the research nurse when the participant is enrolled and introduce the participant for 6MWT. The research nurse, after administering 6MWT (using standardized recommendations from the American Thoracic Society) will inform the research coordinator of the result, which the coordinator will then enter into REDCap.

This material is the property of the NYU School of Medicine and Langone Medical Center. Do not disclose or use except as authorized in writing by the study sponsor

We do not plan to unmask the blinded 6MWT assessor as this may interfere with integrity of the study randomization. We anticipate that adverse events during 6MWT will be very rare, and if they occur, it will not be necessary for the assessor to know the participants' treatment assignment.

10.6.2 Evaluation of Success of Blinding

The blinded 6MWT assessor will not be given access to any information that may reveal the subjects' study arm; it is not anticipated that they will become unblinded. After completion of the 3-month follow-up visit, the 6MWT assessor will be asked if they had become aware of the subject's study arm assignment prior to completing the 6MWT evaluation. If the assessor affirms unblinding, this information will be noted on the 6MWT CRF.

10.6.3 Breaking the Study Blind/Participant Code

There are no plans to unmask the blinded research nurse/6MWT assessor at any time. No other study staff or participants will be blinded.

11 Source Documents and Access to Source Data/Documents

Source data is all information, original records of clinical findings, observations, or other activities in a clinical trial necessary for the reconstruction and evaluation of the trial. Source data are contained in source documents. Examples of these original documents, and data records include: hospital records, clinical and office charts, laboratory notes, memoranda, subjects' diaries or evaluation checklists, pharmacy dispensing records, recorded data from automated instruments, copies or transcriptions certified after verification as being accurate and complete, microfiches, photographic negatives, microfilm or magnetic media, x-rays, subject files, and records kept at the pharmacy, at the laboratories, and at medico-technical departments involved in the clinical trial.

The study case report form (CRF) is the primary data collection instrument for the study. All data requested on the CRF must be recorded. All missing data must be explained. If a space on the CRF is left blank because the procedure was not done or the question was not asked, write "N/D". If the item is not applicable to the individual case, write "N/A". All entries should be printed legibly in black ink. If any entry error has been made, to correct such an error, draw a single straight line through the incorrect entry and enter the correct data above it. All such changes must be initialed and dated. DO NOT ERASE OR WHITE OUT ERRORS. For clarification of illegible or uncertain entries, print the clarification above the item, then initial and date it.

Access to study records will be limited to IRB-approved members of the study team. The investigator will permit study-related monitoring, audits, and inspections by the IRB/EC, government regulatory bodies, and University compliance and quality assurance groups of all study related documents (e.g. source documents, regulatory documents, data collection instruments, study data etc.). The investigator will ensure the capability for inspections of applicable study-related facilities (e.g. pharmacy, diagnostic laboratory, etc.).

Participation as an investigator in this study implies acceptance of potential inspection by government regulatory authorities and applicable University compliance and quality assurance offices.

12 Quality Assurance and Quality Control

QC procedures will be implemented beginning with the data entry system and data QC checks that will be run on the database will be generated. Any missing data or data anomalies will be communicated to the site(s) for clarification/resolution.

CONFIDENTIAL
This material is the property of the NYU School of Medicine and Langone Medical Center. Do not disclose or use except as authorized in writing by the study sponsor

Following written SOPs, the monitors will verify that the clinical trial is conducted and data are generated, documented (recorded), and reported in compliance with the protocol, GCP, and the applicable regulatory requirements (e.g., Good Laboratory Practices (GLP), Good Manufacturing Practices (GMP)).

The investigational site will provide direct access to all trial related sites, source data/documents, and reports for the purpose of monitoring and auditing and inspection by local and regulatory authorities.

13 Ethics/Protection of Human Subjects

13.1 Ethical Standard

The investigator will ensure that this study is conducted in full conformity with Regulations for the Protection of Human Subjects of Research codified in 45 CFR Part 46, 21 CFR Part 50, 21 CFR Part 56, and/or the ICH E6.

13.2 Institutional Review Board

The protocol, informed consent form(s), recruitment materials, and all participant materials will be submitted to the IRB for review and approval. Approval of both the protocol and the consent form must be obtained before any participant is enrolled. Any amendment to the protocol will require review and approval by the IRB before the changes are implemented to the study. All changes to the consent form will be IRB approved; a determination will be made regarding whether previously consented participants need to be re-consented.

13.3 Informed Consent Process

13.3.1 Consent/Assent and Other Informational Documents Provided to Participants

Consent forms describing in detail the study procedures, and risks are given to the participant and written documentation of informed consent is required prior to starting intervention/administering study product. The following consent materials are submitted with this protocol: Template Consent Form.

13.3.2 Consent Procedures and Documentation

Informed consent is a process that is initiated prior to the individual’s agreeing to participate in the study and continues throughout the individual’s study participation. Extensive discussion of risks and possible benefits of participation will be provided to the participants and their families. Consent forms will be IRB-approved and the participant will be asked to read and review the document. The RC will explain the research study to the participant and answer any questions that may arise. The PI will be available for any questions that cannot be answered by the RC. All participants will receive a verbal explanation in terms suited to their comprehension of the purposes and potential risks of the study and of their rights as research participants. Participants will have the opportunity to carefully review the written consent form and ask questions prior to signing. The participants should have the opportunity to discuss the study with their surrogates or think about it prior to agreeing to participate. The participant will sign the informed consent document prior to any procedures being done specifically for the study. The participants may withdraw consent at any time throughout the course of the trial. A copy of the signed informed consent document will be given to the participants for their records. The rights and welfare of the participants will be protected by emphasizing to them that the quality of their medical care will not be adversely affected if they decline to participate in this study.

A copy of the signed informed consent document will be stored in the subject’s research record. The consent process, including the name of the individual obtaining consent, will be thoroughly documented in the subject’s research record. Any alteration to the standard consent process (e.g. use of a translator, consent

CONFIDENTIAL
This material is the property of the NYU School of Medicine and Langone Medical Center. Do not disclose or use except as authorized in writing by the study sponsor

from a legally authorized representative, consent document presented orally, etc.) and the justification for such alteration will likewise be documented.

13.4 Participant and Data Confidentiality

Information about study subjects will be kept confidential and managed according to the requirements of the Health Insurance Portability and Accountability Act of 1996 (HIPAA). Those regulations require a signed subject authorization informing the subject of the following:

- What protected health information (PHI) will be collected from subjects in this study
- Who will have access to that information and why
- Who will use or disclose that information
- The rights of a research subject to revoke their authorization for use of their PHI.

At Bellevue Hospital, a Waiver of HIPAA Authorization granted by the NYU Langone IRB will be used to conduct standard screening and recruitment procedures outlined in section 7.2.1.

In the event that a subject revokes authorization to collect or use PHI, the investigator, by regulation, retains the ability to use all information collected prior to the revocation of subject authorization. For subjects that have revoked authorization to collect or use PHI, attempts should be made to obtain permission to collect at least vital status (i.e. that the subject is alive) at the end of their scheduled study period.

Participant confidentiality is strictly held in trust by the participating investigators, their staff, and the sponsor(s) and their agents. This confidentiality is extended to cover testing of biological samples and genetic tests in addition to the clinical information relating to participants. Therefore, the study protocol, documentation, data, and all other information generated will be held in strict confidence. No information concerning the study or the data will be released to any unauthorized third party without prior written approval of the sponsor.

The study monitor, other authorized representatives of the sponsor, or representatives of the IRB may inspect all documents and records required to be maintained by the investigator, including but not limited to, medical records (office, clinic, or hospital) and pharmacy records for the participants in this study. The clinical study site will permit access to such records.

For participants receiving the mHealth-CR software intervention, the following information will be collected through their respective applications:

- FitBit (wearable activity monitor): passive physiological activity monitoring (e.g. step count, time of sedentary vs. active activity). Activity is categorized (based on step count) as sedentary or mildly, moderately, or vigorously active. Data are automatically uploaded daily to Moving Analytics, and will be viewable by both the study participant and the exercise therapist.
- Moving Analytics (mHealth-CR software): passive physiological activity monitoring (e.g. step count synced with FitBit); self-reported step count; self-reported exercise activity, duration, and RPE; self-reported blood pressure measurements; and communication (chat messages) with the study team.

No PHI will be collected through the mHealth-CR software platforms or the tablet. Patients will be provided with a random study number that is linked to a master file in the core dataset, therefore ensuring confidentiality of data from devices. All data obtained from the intervention platforms will be initially stored in a secure HIPAA-compliant cloud-based platform provided by its respective companies (FitBit, Moving Analytics), and later securely transferred to research staff at NYU for storage in the REDCap database. All study participants in the intervention arm will be advised during the consenting process to only use the provided study device (tablet) for research-related purposes only in order to prevent leakage of PHI-related data.

The study participant's contact information will be securely stored at each clinical site for internal use during the study. At the end of the study, all records will continue to be kept in a secure location for as long a period as dictated by local IRB and Institutional regulations.

CONFIDENTIAL
This material is the property of the NYU School of Medicine and Langone Medical Center. Do not disclose or use except as authorized in writing by the study sponsor

Study participant research data, which is for purposes of statistical analysis and scientific reporting, will be transmitted to and stored at the NYU Translational Research Building in password-protected computers and physically locked in cabinet files at the desks of the research coordinator. The study data entry and study management systems used by clinical sites and by NYU Langone Medical Center research staff will be secured and password protected. At the end of the study, all study databases will be de-identified and archived at the NYU Langone Medical Center.

To further protect the privacy of study participants, a Certificate of Confidentiality will be obtained from the NIH. This certificate protects identifiable research information from forced disclosure. It allows the investigator and others who have access to research records to refuse to disclose identifying information on research participation in any civil, criminal, administrative, legislative, or other proceeding, whether at the federal, state, or local level. By protecting researchers and institutions from being compelled to disclose information that would identify research participants, Certificates of Confidentiality help achieve the research objectives and promote participation in studies by helping assure confidentiality and privacy to participants.

13.4.2 Research Use of Stored Human Samples, Specimens, or Data

No human samples or specimens are collected for this study. Data will be collected.

- Intended Use: Data collected under this protocol may be used to study ischemic heart disease and its benefits from cardiac rehabilitation. No laboratory or genetic testing will be performed.
- Storage: Data will be stored using codes assigned by the investigators. Data will be kept in password-protected computers. Only investigators and the RCs will have access to the data.
- Tracking: Data will be tracked using REDCap.
    - Disposition at the completion of the study: All stored data will be sent to the REDCap database. Study participants who request destruction of data will be notified of compliance with such request and all supporting details will be maintained for tracking.

14 Data Handling and Record Keeping

14.1 Data Collection and Management Responsibilities

The research material will be the prospectively collected data from described study procedures comprised of clinical data collection, questionnaires, and physiological data recordings collected from each participant. All of these research resources will be obtained specifically for research purposes. All records with identifying information for NYU participants will be kept in a locked cabinet in the office of the contact Principal Investigator (Dr. Dodson) or as a password-protected file on the secure NYU network or in a password protected computer. Records with identifying information for NYU-Winthrop, UMMHC and Yale participants will initially be stored at NYU-Winthrop, UMMHC and Yale (in the office of Site Sub-Investigators and Site PI, respectively, or on a secure network) and subsequently transferred securely to NYU for analysis. For intervention participants, data on engagement with the study intervention (Moving Analytics) will initially be stored in a secure HIPAA- compliant cloud-based platform provided by Moving Analytics, which has been previously used in both clinical and research settings. For analytic purposes, these data will then be securely transferred to staff at NYU Langone Health DataCore.

Data collection is the responsibility of the clinical trial staff at the site under the supervision of the site PI. The investigator is responsible for ensuring the accuracy, completeness, legibility, and timeliness of the data reported.

All source documents should be completed in a neat, legible manner to ensure accurate interpretation of data. Black ink is required to ensure clarity of reproduced copies. When making changes or corrections, cross out the original entry with a single line, and initial and date the change. DO NOT ERASE, OVERWRITE, OR USE CORRECTION FLUID OR TAPE ON THE ORIGINAL.

CONFIDENTIAL
This material is the property of the NYU School of Medicine and Langone Medical Center. Do not disclose or use except as authorized in writing by the study sponsor

Copies of the electronic CRF (eCRF) will be provided for use as source documents and maintained for recording data for each participant enrolled in the study. Data reported in the eCRF derived from source documents should be consistent with the source documents or the discrepancies should be explained and captured in a progress note and maintained in the participant's official electronic study record.

Clinical data (including AEs, concomitant medications, and expected adverse reactions data) will be entered into REDCap, a 21 CFR Part 11-compliant data capture system provided by the NYULH DataCore. The data system includes password protection and internal quality checks, such as automatic range checks, to identify data that appear inconsistent, incomplete, or inaccurate. Clinical data will be entered directly from the source documents.

14.2 Study Records Retention

Study documents will be retained for the longer of 3 years after close-out, 5 years after final reporting/publication, or 2 years after the investigation is discontinued. No records will be destroyed without the written consent of the sponsor, if applicable. It is the responsibility of the sponsor to inform the investigator when these documents no longer need to be retained.

14.3 Protocol Deviations

A protocol deviation is any noncompliance with the clinical trial protocol or GCP requirements. The noncompliance may be either on the part of the participant, the investigator, or the study site staff. As a result of deviations, corrective actions are to be developed by the site and implemented promptly.

These practices are consistent with ICH E6:

- 4.5 Compliance with Protocol, sections 4.5.1, 4.5.2, and 4.5.3
- 5.1 Quality Assurance and Quality Control, section 5.1.1
- 5.20 Noncompliance, sections 5.20.1, and 5.20.2.

It is the responsibility of the site PI/study staff to use continuous vigilance to identify and report deviations within 5 working days of identification of the protocol deviation, or within 5 working days of the scheduled protocol-required activity.

All protocol deviations must be addressed in study source documents, reported to NIA Program Official.

Protocol deviations must be reported to the local IRB per their guidelines. The site PI/study staff is responsible for knowing and adhering to their IRB requirements. Further details about the handling of protocol deviations will be included in the MOP.

14.4 Publication and Data Sharing Policy

This study will comply with the NIH Public Access Policy, which ensures that the public has access to the published results of NIH funded research. It requires scientists to submit final peer-reviewed journal manuscripts that arise from NIH funds to the digital archive PubMed Central upon acceptance for publication.

The International Committee of Medical Journal Editors (ICMJE) member journals have adopted a clinical trials registration policy as a condition for publication. The ICMJE defines a clinical trial as any research project that prospectively assigns human subjects to intervention or concurrent comparison or control groups to study the cause-and-effect relationship between a medical intervention and a health outcome. Medical interventions include drugs, surgical procedures, devices, behavioral treatments, process-of-care changes, and the like. Health outcomes include any biomedical or health-related measures obtained in patients or participants, including pharmacokinetic measures and adverse events. The ICMJE policy, and the Section

This material is the property of the NYU School of Medicine and Langone Medical Center. Do not disclose or use except as authorized in writing by the study sponsor

801 of the Food and Drug Administration Amendments Act of 2007, requires that all clinical trials be registered in a public trials registry such as ClinicalTrials.gov, which is sponsored by the National Library of Medicine. Other biomedical journals are considering adopting similar policies. For interventional clinical trials performed under NIH IC grants and cooperative agreements, it is the grantee's responsibility to register the trial in an acceptable registry, so the research results may be considered for publication in ICMJE member journals. The ICMJE does not review specific studies to determine whether registration is necessary; instead, the committee recommends that researchers who have questions about the need to register err on the side of registration or consult the editorial office of the journal in which they wish to publish.

FDAAA mandates that a "responsible party" (i.e., the sponsor or designated principal investigator) register and report results of certain "applicable clinical trials":

- Trials of Drugs and Biologics: Controlled, clinical investigations, other than Phase I investigations of a product subject to FDA regulation;
- Trials of Devices: Controlled trials with health outcomes of a product subject to FDA regulation (other than small feasibility studies) and pediatric postmarket surveillance studies.
- NIH grantees must take specific steps to ensure compliance with NIH implementation of FDAAA.

15 Study Finances

15.1 Funding Source

This study is financed through a grant from the US National Institute of Health.

15.2 Costs to the Participant

The study has built-in transportation costs for the 3-month follow-up assessment visit. In addition, study equipment will be provided to participants at no charge, and a toll-free number will be provided to reach the research coordinator for ongoing communication as necessary. There are no anticipated out-of-pocket costs for participants using the mobile health software application. A home WiFi network is not required. Tablet devices will have cellular capability, provided by the study.

Study-related costs are funded by the grant.

However, if a participant loses any of the devices during the study, s/he may be responsible for the cost of replacing the equipment. If there are damages made to the devices, the participant may be responsible for the cost of repair or replacement.

15.3 Participant Reimbursements or Payments

A total compensation of $115.00 will be provided for study participants who complete all study related visits.

$25 will be compensated to participants after completion of the first baseline visit, and $90 will be compensated to participants after completion of the 3-month ambulatory visit.

16 Study Administration

16.1 Study Leadership

The Steering Committee will govern the conduct of the study. The Committee will comprise of the site PIs, co-investigators, the Project Coordinator, and the NIA Program Officer. The Steering Committee will meet

This material is the property of the NYU School of Medicine and Langone Medical Center. Do not disclose or use except as authorized in writing by the study sponsor

convene three times a year; once in person (at the American College of Cardiology Scientific Sessions) and twice via teleconference.

17 Conflict of Interest Policy

The independence of this study from any actual or perceived influence, such as by the pharmaceutical industry, is critical. Therefore any actual conflict of interest of persons who have a role in the design, conduct, analysis, publication, or any aspect of this trial will be disclosed and managed. Furthermore, persons who have a perceived conflict of interest will be required to have such conflicts managed in a way that is appropriate to their participation in the trial. The study leadership in conjunction with the NIH/NIA has established policies and procedures for all study group members to disclose all conflicts of interest and will establish a mechanism for the management of all reported dualities of interest.

Any investigator who has a conflict of interest with this study (patent ownership, royalties, or financial gain greater than the minimum allowable by their institution, etc.) must have the conflict reviewed by the NYU Langone Conflict of Interest Management Unit (CIMU) with a Committee-sanctioned conflict management plan that has been reviewed and approved by the study sponsor prior to participation in this study. All NYULMC investigators will follow the applicable conflict of interest policies.

18 References

1. Mazzini MJ, Stevens GR, Whalen D, Ozonoff A, Balady GJ. Effect of an American Heart Association Get With the Guidelines Program-Based Clinical Pathway on Referral and Enrollment Into Cardiac Rehabilitation After Acute Myocardial Infarction. *Am J Cardiol*. 2008;101:1084-1087.
2. Thomas RJ, King M, Lui K, Oldridge N, Piña IL, Spertus J. AACVPR/ACC/AHA 2007 performance measures on cardiac rehabilitation for referral to and delivery of cardiac rehabilitation/secondary prevention services. *Circulation*. 2007;116:1611-1642.
3. Aragam KG, Dai D, Neely ML, et al. Gaps in referral to cardiac rehabilitation of patients undergoing percutaneous coronary intervention in the United States. *J Am Coll Cardiol*. 2015;65:2079-2088.
4. Dunlay SM, Witt BJ, Allison TG, et al. Barriers to participation in cardiac rehabilitation. *Am Heart J*. 2009;158:852-859.
5. Varnfield M, Karunanithi M, Lee C-K, et al. Smartphone-based home care model improved use of cardiac rehabilitation in postmyocardial infarction patients: Results from a randomised controlled trial. *Heart*. 2014;100:1770-1779.
6. Grace SL, Shanmugasegaram S, Gravely-Witte S, Brual J, Suskin N, Stewart DE. Barriers to cardiac rehabilitation: Does age make a difference? *J Cardiopulm Rehabil Prev*. 2009;29:183-187.
7. Witt BJ, Jacobsen SJ, Weston SA, et al. Cardiac rehabilitation after myocardial infarction in the community. *J Am Coll Cardiol*. 2004;44:988-996.
8. Fried TR, McGraw S, Agostini J V., Tinetti ME. Views of older persons with multiple morbidities on competing outcomes and clinical decision-making. *J Am Geriatr Soc*. 2008;56:1839-1844.
9. Fried TR, Bradley EH, Towle VR, Allore H. Understanding the treatment preferences of seriously ill patients. *N Engl J Med*. 2002;346:1061-1066.
10. Widmer RJ, Allison TG, Lerman LO, Lerman A. Digital health intervention as an adjunct to cardiac rehabilitation reduces cardiovascular risk factors and rehospitalizations. *J Cardiovasc Transl Res*. 2015;8:283-292. PMCID: PMC4690207
11. Widmer RJ, Allison TG, Lennon R, Lopez-Jimenez F, Lerman LO, Lerman A. Digital health intervention during cardiac rehabilitation: A randomized controlled trial. *Am Heart J*. 2017;188:65-72.
12. Turner-Stokes L. Goal attainment scaling (GAS) in rehabilitation: A practical guide. *Clin Rehabil*.

This material is the property of the NYU School of Medicine and Langone Medical Center. Do not disclose or use except as authorized in writing by the study sponsor

2009;23:362-370.

13. Goel K, Lennon RJ, Tilbury RT, Squires RW, Thomas RJ. Impact of cardiac rehabilitation on mortality and cardiovascular events after percutaneous coronary intervention in the community. *Circulation*. 2011;123:2344-2352.

14. Anderson L, Oldridge N, Thompson DR, et al. Exercise-based cardiac rehabilitation for coronary heart disease: Cochrane systematic review and meta-analysis. *J Am Coll Cardiol*. 2016;67:1-12.

15. Lavie CJ, Milani R V. Effects of cardiac rehabilitation programs on exercise capacity, coronary risk factors, behavioral characteristics, and qualify of life in a large elderly cohort. *Am J Cardiol*. 1995;76:177-179.

16. Smith SC, Benjamin EJ, Bonow RO, et al. AHA/ACCF secondary prevention and risk reduction therapy for patients with coronary and other atherosclerotic vascular disease: 2011 update: A guideline from the American Heart Association and American College of Cardiology Foundation. *Circulation*. 2011;124:2458-2473.

17. Krumholz HM, Anderson JL, Bachelder BL, Fesmire FM, Fihn SD, Foody JM, Ho PM, Kosiborod MN, Masoudi FA, Nallamothu BK. ACC/AHA 2008 performance measures for adults with ST-elevation and Non–ST-elevation myocardial infarction: A report of the American College of Cardiology/American Heart Association task force on performance measures (writing committee to develop performance measures for ST-elevation and Non–ST-elevation myocardial infarction) developed in collaboration with the American Academy of Family Physicians and American College of Emergency Physicians endorsed by the American Association of Cardiovascular and Pulmonary Rehabilitation, Society for Cardiovascular Angiography and Interventions, and Society of Hospital Medicine. *J Am Coll Cardiol*. 2008;52:2046-2099.

18. Doll JA, Hellkamp A, Ho PM, et al. Participation in cardiac rehabilitation programs among older patients after acute myocardial infarction. *JAMA Intern Med*. 2015;175:1700-1702.

19. Roe MT, Messenger JC, Weintraub WS, et al. Treatments, trends, and outcomes of acute myocardial infarction and percutaneous coronary intervention. *J Am Coll Cardiol*. 2010;56:254-263.

20. Blackwell DL, Lucas JW, Clarke TC. Summary health statistics for U.S. adults: National health interview survey, 2012. *Vital Health Stat 10*. 2014;10:1-171.

21. Aragam KG, Moscucci M, Smith DE, et al. Trends and disparities in referral to cardiac rehabilitation after percutaneous coronary intervention. *Am Heart J*. 2011;161:544-551.

22. Thomas RJ, King M, Lui K, Oldridge N, Pia IL, Spertus J. AACVPR/ACCF/AHA 2010 update: Performance measures on cardiac rehabilitation for referral to cardiac rehabilitation/secondary prevention services. *J Am Coll Cardiol*. 2010;56:1159-1167.

23. Beatty AL, Fukuoka Y, Whooley MA. Using mobile technology for cardiac rehabilitation: A review and framework for development and evaluation. *J Am Heart Assoc*. 2013;2:e000568. PMCID: PMC3886753.

24. Helbostad J, Vereijken B, Becker C, et al. Mobile health applications to promote active and healthy ageing. *Sensors*. 2017;17:622. PMCID: PMC5375908.

25. Mosca L, Mochari H, Liao M, et al. A novel family-based intervention trial to improve heart health: FIT heart: Results of a randomized controlled trial. *Circ Cardiovasc Qual Outcomes*. 2008;1:98-106. PMCID: PMC2801902.

26. Sivarajan Froelicher ES, Miller NH, Christopherson DJ, et al. High Rates of sustained smoking cessation in women hospitalized with cardiovascular disease: The Women's Initiative for Nonsmoking (WINS). *Circulation*. 2004;109:587-593.

27. DeBusk RF, Miller NH, Superko HR, et al. A case-management system for coronary risk factor modification after acute myocardial infarction. *Ann Intern Med*. 1994;120:721-729.

28. James J. Health policy brief: Patient engagement. *Health Affairs*. February 14, 2013; http://www.healthaffairs.org/healthpolicybriefs/brief.php?brief_id=86. Accessed May 3, 2018.

29. Bonaccorso S, Sturchio JL. Perspectives from the pharmaceutical industry. *BMJ.* 2003;327:863-864. PMCID: PMC214048.

This material is the property of the NYU School of Medicine and Langone Medical Center. Do not disclose or use except as authorized in writing by the study sponsor

30. Schwarzer R. Modeling health behavior change: How to predict and modify the adoption and maintenance of health behaviors. *Appl Psychol*. 2008;57:1-29.

31. Sniehotta FF, Scholz U, Schwarzer R. Action plans and coping plans for physical exercise: A longitudinal intervention study in cardiac rehabilitation. *Br J Health Psychol*. 2006;11:23-37.

32. Venkatesh V, Bala H. Technology acceptance model 3 and a research agenda on interventions. *Decision Sciences*. 2008;39:273-315.

33. Gell NM, Rosenberg DE, Demiris G, LaCroix AZ, Patel K V. Patterns of technology use among older adults with and without disabilities. *Gerontologist*. 2015;55:412-421. PMCID: PMC4542705.

34. Hong SG, Trimi S, Kim DW. Smartphone use and internet literacy of senior citizens. *J Assist Technol*. 2016;10:27-38.

35. Goswami A, Dutta S. Gender Differences in Technology Usage — A literature review. *Open Journal of Business and Management*. 2016;4:51-59.

36. Cole H, Schoenthaler A, Braithwaite RS, et al. Community-based settings and sampling strategies: Implications for reducing racial health disparities among black men, New York City, 2010–2013. *Prev Chronic Dis*. 2014;11:E105. PMCID: PMC4068114.

37. Schoenthaler A, Luerassi L, Teresi JA, et al. A practice-based trial of blood pressure control in African Americans (TLC-Clinic): Study protocol for a randomized controlled trial. *Trials*. 2011;12. PMCID: PMC3264527.

38. Schoenthaler A, De La Calle F, Barrios-Barrios M, et al. A practice-based randomized controlled trial to improve medication adherence among Latinos with hypertension: Study protocol for a randomized controlled trial. *Trials*. 2015;16:290. PMCID: PMC4488119.

39. Schoenthaler A, Luerassi L, Silver S, et al. Comparative effectiveness of a practice-based comprehensive lifestyle intervention vs. single session counseling in hypertensive blacks. *Am J Hypertens*. 2016;29:280-287. PMCID: PMC4751244.

40. Ogedegbe G, Chaplin W, Schoenthaler A, et al. A practice-based trial of motivational interviewing and adherence in hypertensive African Americans. *Am J Hypertens*. 2008;21:1137-1143. PMCID: PMC3747638.

41. Ogedegbe G, Tobin JN, Fernandez S, et al. Counseling African Americans to control hypertension (CAATCH) trial: A multi-level intervention to improve blood pressure control in hypertensive blacks. *Circ Cardiovasc Qual Outcomes*. 2009;2:249-256. PMCID: PMC2800792.

42. Dodson JA, Geda M, Krumholz HM, et al. Design and rationale of the comprehensive evaluation of risk factors in older patients with AMI (SILVER-AMI) study. *BMC Health Serv Res*. 2014;14. PMCID: PMC4239317.

43. Gill TM, Pahor M, Guralnik JM, et al. Effect of structured physical activity on prevention of serious fall injuries in adults aged 70-89: Randomized clinical trial (LIFE study). *BMJ*. 2016;352. PMCID: PMC4772786.

44. Gill TM, Guralnik JM, Pahor M, Church T, et al. Effect of structured physical activity on overall burden and transitions between states of major mobility disability in older persons: Secondary analysis of a randomized trial. *Ann Intern Med*. 2016;165:833-840. PMCID: PMC5476905.

45. Bhasin S, Gill TM, Reuben DB, et al. Strategies to reduce injuries and develop confidence in elders (STRIDE): A cluster-randomized pragmatic trial of a multifactorial fall injury prevention strategy: Design and methods. *J Gerontol A Biol Sci Med Sci.* 2017:glx190-glx190.

46. Inouye SK, Bogardus ST, Charpentier PA, et al. A multicomponent intervention to prevent delirium in hospitalized older patients. *N Engl J Med*. 1999;340:669-676.

47. Gill TM, Baker DI, Gottschalk M, Peduzzi PN, Allore H, Byers A. A program to prevent functional decline in physically frail, elderly persons who live at home. *N Engl J Med*. 2002;347:1068-1074.

48. Gill TM. Disentangling the disabling process: Insights from the precipitating events project. *Gerontologist*. 2014;54:533-549. PMCID: PMC4155452.

CONFIDENTIAL
This material is the property of the NYU School of Medicine and Langone Medical Center. Do not disclose or use except as authorized in writing by the study sponsor

49. Balke B. A simple field test for the assessment of physical fitness. *Rep Civ Aeromed Res Inst US.* 1963;53:1-8..

50. O'Keeffe ST, Lye M, Donnellan C, Carmichael DN. Reproducibility and responsiveness of quality of life assessment and six minute walk test in elderly heart failure patients. *Heart*. 1998;80:377-382. PMCID: PMC1728807.

51. Bittner V, Weiner DH, Yusuf S, et al. Prediction of mortality and morbidity with a 6-minute walk test in patients with left ventricular dysfunction. *JAMA*. 1993;270:1702-1707.

52. ATS Committee on Proficiency Standards for Clinical Pulmonary Function Laboratories. ATS statement: Guidelines for the six-minute walk test. *Am J Respir Crit Care Med*. 2002;166:111-117

53. Beatty AL, Schiller NB, Whooley MA. Six-minute walk test as a prognostic tool in stable coronary heart disease: Data from the Heart and Soul Study. *Arch Intern Med*. 2012;172:1096-1102. PMCID: PMC3420342.

54. Shah MR, Hasselblad V, Gheorghiade M, et al. Prognostic usefulness of the six-minute walk in patients with advanced congestive heart failure secondary to ischemic or nonischemic cardiomyopathy. *Am J Cardiol*. 2001;88:987-993.

55. Cacciatore F, Abete P, Mazzella F, et al. Six-minute walking test but not ejection fraction predicts mortality in elderly patients undergoing cardiac rehabilitation following coronary artery bypass grafting. *Eur J Prev Cardiol*. 2012;19:1401-1409.

56. La Rovere MT, Pinna GD, Maestri R, et al. The 6-minute walking test and all-cause mortality in patients undergoing a post-cardiac surgery rehabilitation program. *Eur J Prev Cardiol*. 2015;22:20-26.

57. Bellet RN, Adams L, Morris NR. The 6-minute walk test in outpatient cardiac rehabilitation: Validity, reliability and responsiveness-a systematic review. *Physiotherapy*. 2012;98:277-287.

58. O'Connor CM, Whellan DJ, Lee KL, et al. Efficacy and safety of exercise training in patients with chronic heart failure: HF-ACTION randomized controlled trial. *JAMA*. 2009;301:1439-1450. PMCID: PMC2916661.

59. Minneboo M, Lachman S, Snaterse M, et al. Community-based lifestyle intervention in patients with coronary artery disease. *J Am Coll Cardiol*. 2017;70:318-327.

60. Alharbi M, Gallagher R, Kirkness A, Sibbritt D, Tofler G. Long-term outcomes from healthy eating and exercise lifestyle program for overweight people with heart disease and diabetes. *Eur J Cardiovasc Nurs*. 2016;15:91-99.

61. Oliveira GU, Carvalho VO, de Assis Cacau LP, et al. Determinants of distance walked during the six-minute walk test in patients undergoing cardiac surgery at hospital discharge. *J Cardiothorac Surg*. 2014;9:95. PMCID: PMC4064506.

62. Peixoto TC, Begot I, Bolzan DW, et al. Early exercise-based rehabilitation improves health-related quality of life and functional capacity after acute myocardial infarction: a randomized controlled trial. *Can J Cardiol*. 2015;31:308-313.

63. Tinetti, M, Naik, A, Dodson JA. Moving from disease-centered to patient goals–directed care for patients with multiple chronic conditions: Patient value-based care. *JAMA Cardiol*. 2016;1:9-10.

64. Hurn J, Kneebone I, Cropley M. Goal setting as an outcome measure: A systematic review. *Clin Rehabil*. 2006;20:756-772.

65. Ware JE, Kosinski M, Keller SD. A 12-Item Short-Form Health Survey: Construction of scales and preliminary tests of reliability and validity. *Med Care*. 1996;34:220-233.

66. Chan PS, Jones PG, Arnold SA, Spertus JA. Development and validation of a short version of the Seattle Angina Questionnaire. *Circ Cardiovasc Qual Outcomes*. 2014;7:640-647. PMCID: PMC4282595.

67. Rumsfeld JS, Alexander KP, Goff DC, et al. Cardiovascular health: The importance of measuring patient-reported health status. A scientific statement from the American Heart Association. *Circulation*. 2013;127:2233-2249.

68. Porter ME, Larsson S, Lee TH. Standardizing patient outcomes measurement. *N Engl J Med*.

CONFIDENTIAL
This material is the property of the NYU School of Medicine and Langone Medical Center. Do not disclose or use except as authorized in writing by the study sponsor

2016;374:504-506.

69. Arnold SV, Alexander KP, Masoudi FA, Ho PM, Xiao L, Spertus JA. The effect of age on functional and mortality outcomes after acute myocardial infarction. *J Am Geriatr Soc*. 2009;57:209-217.

70. Katz S. Assessing self-maintenance: Activities of daily living, mobility, and instrumental activities of daily living. *J Am Geriatr Soc*. 1983;31:721-727.

71. Dodson JA, Arnold SV, Reid KJ, et al. Physical function and independence 1 year after myocardial infarction: Observations from the Translational Research Investigating Underlying disparities in recovery from acute myocardial infarction: Patients' health status registry. *Am Heart J*. 2012;163:790-796. PMCID: PMC3359897.

72. Bradley EH, Curry L, Horwitz LI, et al. Contemporary evidence about hospital strategies for reducing 30-day readmissions: A national study. *J Am Coll Cardiol*. 2012;60:607-614. PMCID: PMC3537181.

73. Jolly K, Lip GYH, Taylor RS, Raftery J, et al. The Birmingham Rehabilitation Uptake Maximisation study (BRUM): A randomised controlled trial comparing home-based with centre-based cardiac rehabilitation. *Heart*. 2009;95:36-42.

74. Ades PA, Pashkow FJ, Fletcher G, Pina IL, Zohman LR, Nestor JR. A controlled trial of cardiac rehabilitation in the home setting using electrocardiographic and voice transtelephonic monitoring. *Am Heart J*. 2000;139:543-548.

75. U.S. National Library of Medicine. *Empowered With Movement to Prevent Obesity and Weight Regain (EMPOWER)*. https://clinicaltrials.gov/ct2/show/NCT02923674. Published 2018. Accessed May 8, 2018.

76. McDermott MM, Spring B, Berger JS, et al. Effect of a home-based exercise intervention of wearable technology and telephone coaching on walking performance in peripheral artery disease: The HONOR randomized clinical trial. *JAMA*. 2018;319:1665-1676. PMCID: PMC5933394.

77. United States Department of Health and Human Services Office of Disease Prevention and Health. *Physical activity guidelines: Active older adults*. https://health.gov/paguidelines/guidelines/chapter5.aspx. Accessed May 1, 2018.

78. Lennon O, Carey A, Gaffney N, Stephenson J, Blake C. A pilot randomized controlled trial to evaluate the benefit of the cardiac rehabilitation paradigm for the non-acute ischaemic stroke population. *Clin Rehabil*. 2008;22:125-133.

79. Pahor M, Guralnik JM, Ambrosius WT, et al. Effect of structured physical activity on prevention of major mobility disability in older adults: The LIFE study randomized clinical trial. *JAMA*. 2014;311:2387-2396. PMCID: PMC4266388.

80. Snyder PJ, Bhasin S, Cunningham GR, et al. Effects of Testosterone Treatment in Older Men. *N Engl J Med*. 2016;374:611-624. PMCID: PMC5209754.

81. Yang L, Freed D, Wu A, Wu J, Pollak JP, Estrin D. Your activities of daily living (YADL): An image-based survey technique for patients with arthritis. Proceedings of the 10th EAI International Conference on Pervasive Computing Technologies for Healthcare; May 2016; Cancun, MX. *Pervasive Health.* 41-44.

82. Fried LP, Tangen CM, Walston J, Newman AB, Hirsch C, Gottdiener J, Seeman T, Tracy R, Kop WJ, Burke G, McBurnie MA. Frailty in older adults: Evidence for a phenotype. *J Gerontol A Biol Sci Med Sci*. 2001;56:M146-156.

83. O'Brien HL, Toms EG. What is user engagement? A conceptual framework for defining user engagement with technology. *J Am Soc Inf Sci Technol*. 2008;59:938-955.

84. Mickey RM, Greenland S. The impact of confounder selection criteria on effect estimation*. Am J Epidemiol*. 1989;129:125-137.

85. Rothman KJ, Greenland S, Lash TL. *Modern epidemiology.* 3. rev. ed. Philadelphia, PA: Lippincott, Williams & Wilkins; 2012.

86. Gunzler D, Chen T, Wu P, Zhang H. Introduction to mediation analysis with structural equation modeling. *Shanghai Arch Psychiatry*. 2013;25:390-394. PMCID: PMC4054581.

CONFIDENTIAL
This material is the property of the NYU School of Medicine and Langone Medical Center. Do not disclose or use except as authorized in writing by the study sponsor

87. Muthén B, Asparouhov T. Causal effects in mediation modeling: An introduction with applications to latent variables. *Struct Equ Modeling*. 2015;22:12-23.

88. Robins JM, Greenland S. Identifiability and exchangeability for direct and indirect effects. *Epidemiology*. 1992;3:143-155.

89. Krasny-Pacini A, Hiebel J, Pauly F, Godon S, Chevignard M. Goal attainment scaling in rehabilitation: A literature-based update. *Ann Phys Rehabil Med*. 2013;56:212-230.

90. Collins LM, Lanza ST. *Latent class and latent transition analysis.* Hoboken, NJ: Wiley; 2010.

91. Everitt BS. A note on parameter estimation for Lazarsfeld's latent class model using the EM algorithm. *Multivariate Behav Res*. 1984;19:79-89.

92. Agresti A. *Categorical data analysis.* 3. ed. Hoboken, NJ: Wiley; 2013.

93. Akaike H. Factor analysis and AIC. *Psychometrika.* 1987;52:317-332.

94. Schwartz G. Estimating the dimension of a model*. Annals of Statistics*.1978;6:461-464.

95. McLachlan GJ, Peel D. *Finite mixture models*. 2. print. ed. New York, NY: Wiley; 2001.

96. Nagin D. *Group-based modeling of development*. Cambridge, MA: Harvard Univ. Press; 2005.

97. Patton MQ. *Qualitative evaluation and research methods.* 2nd ed. Newbury Park, CA: Sage; 2002.

98. Perera S, Mody SH, Woodman RC, Studenski SA. Meaningful change and responsiveness in common physical performance measures in older adults. *J Am Geriatr Soc*. 2006;54:743-749.

99. Gremeaux V, Troisgros O, Benaïm S, Hannequin A, Laurent Y, Casillas JM, Benaim C. Determining the minimal clinically important difference for the six-minute walk test and the 200-meter fast-walk test during cardiac rehabilitation program in coronary artery disease patients after acute coronary syndrome. *Arch Phys Med Rehabil*. 2011;92:611-619.

100. Niehaves B, Plattfaut R. Internet adoption by the elderly: Employing IS technology acceptance theories for understanding the age-related digital divide. *Eur J Inf Syst*. 2014;23:708-726.

101. Smith A. *Older adults and technology use*. Pew Research Center. http://www.pewinternet.org/2014/04/03/older-adults-and-technology-use. Accessed May 2, 2018.

102. Morawski K, Ghazinouri R, Krumme A, al et. Association of a smartphone application with medication adherence and blood pressure control: The MediSAFE-BP randomized clinical trial. *JAMA Intern Med*. April 2018.

103. Gardner AW, Parker DE, Montgomery PS, Blevins SM. Step-monitored home exercise improves ambulation, vascular function, and inflammation in symptomatic patients with peripheral artery disease: A randomized controlled trial. *J Am Heart Assoc*. 2014;3:e001107. PMCID: PMC4323792.

104. National Institutes of Health. Accrual of human subjects (milestones) policy. https://www.nhlbi.nih.gov/research/funding/human-subjects/accrual-guidelines. Accessed May 1, 2018.

105. Masoudi FA, Ponirakis A, de Lemos JA, et al. Trends in U.S. Cardiovascular Care: 2016 Report From 4 ACC National Cardiovascular Data Registries. *J AM Coll Cardiol*. 2017;69:1427-1450.

106. National Institutes of Health. NIH Policy and Guidelines on the Inclusion of Chilldren as Participants in Research Involving Human Subjects. https://grants.nih.gov/grants/guide/notice-files/not98-024.html. Accessed May 22, 2018.

107. Ogedegbe G, Schoenthaler A, Richardson T, et al. An RCT of the effect of motivational interviewing on medication adherence in hypertensive African Americans: Rationale and design. *Contemp Clin Trials*. 2007;28:169-181.

108. Yancey AK, Ortega AN, Kumanyika SK. Effective recruitment and retention of minority research participants. *Annu Rev Public Health*. 2006;27:1-28.

109. Russell C, Palmer JR, Adams-Campbell LL, Rosenberg L. Follow-up of a large cohort of Black women. *Am J Epidemiol*. 2001;154:845-853.

110. Staffileno BA, Coke LA. Recruiting and retaining young, sedentary, hypertension-prone African

CONFIDENTIAL
This material is the property of the NYU School of Medicine and Langone Medical Center. Do not disclose or use except as authorized in writing by the study sponsor

American women in a physical activity intervention study. *J Cardiovasc Nurs*. 2006;21:208-216.

111. Janson SL, Alioto ME, Boushey HA. Attrition and retention of ethnically diverse subjects in a multicenter randomized controlled research trial. *Control Clin Trials*. 2001;22:236S-43S.

112. Rosal MC, White MJ, Borg A, et al. Translational research at community health centers: challenges and successes in recruiting and retaining low-income Latino patients with type 2 diabetes into a randomized clinical trial. *Diabetes Educ*. 2010;36:733-749.

113. Gibbons RJ, Balady GJ, et al. ACC/AHA 2002 guideline update for exercise testing: Summary article: A report of the American College of Cardiology/American Heart Association Task Force on Practice Guidelines (Committee to Update the 1997 Exercise Testing Guidelines). *Circulation*. 2002;106:1883-1892.

114. Diniz LS, Neves VR, Starke AC, Barbosa MPT, Britto RR, Ribeiro ALP. Safety of early performance of the six-minute walk test following acute myocardial infarction: A cross-sectional study. *Brazilian J Phys Ther*. 2017;21:167-174. PMCID: PMC5537468.

115. Dharmarajan K, Hsieh A, Dreyer RP, et al. Relationship Between Age and Trajectories of Rehospitalization Risk in Older Adults. *J Am Geriatr Soc*. 2016;65:421-426.

116. Arnold SV, Jang JS, Tang F, Graham G, Cohen DJ, Spertus JA. Prediction of residual angina after percutaneous coronary intervention. *Eur Hear J Qual Care Clin Outcomes*. 2015;1:23-30. PMCID: PMC5862017.

117. Grodzinsky A, Arnold SV, Gosch K, et al. Angina frequency after acute myocardial infarction in patients without obstructive coronary artery disease. *Eur Hear J Qual Care Clin Outcomes*. 2015;1:92-99. PMCID: PMC5321550.

118. Dolovich L, Oliver D, Lamarche L, et al. A protocol for a pragmatic randomized controlled trial using the Health Teams Advancing Patient Experience: Strengthening Quality (Health TAPESTRY) platform approach to promote person-focused primary healthcare for older adults. *Implement Sci*. 2016;11:49.

This material is the property of the NYU School of Medicine and Langone Medical Center. Do not disclose or use except as authorized in writing by the study sponsor

19 Attachments

These documents are relevant to the protocol, but they are not considered part of the protocol. They are stored and modified separately. As such, modifications to these documents do not require protocol amendments.

Attachment 1: Sample Key Study Information Form
Attachment 2: Sample Informed Consent Form
Attachment 3: Data Safety and Monitoring Charter

CONFIDENTIAL
This material is the property of the NYU School of Medicine and Langone Medical Center. Do not disclose or use except as authorized in writing by the study sponsor

RESILIENT STATISTICAL ANALYSIS PLAN

**Authors:**	**Samrachana Adhikari, Ph.D. & Andrea B. Troxel, Sc.D.**
**Version Number**	**1.0**
**Date**	**May 20, 2024**
**Study PI**	**John Dodson, M.D.**
**Study Statisticians**	**Samrachana Adhikari, Ph.D. & Andrea B. Troxel, Sc.D.**

Version History
Version 0.1 Submitted to the grant
Version 0.2 Initial draft to discuss with the study team on 03/22/2024
Version 0.3 Multiple imputation added as a part of primary outcome analysis and Bayesian analysis added as sensitivity analysis on April 15, 2024
Version 1.0 Finalized SAP prior to conducting primary outcome analysis May 20, 2024

Table of Contents

1. INTRODUCTION 60
2. STUDY OVERVIEW AND PATIENT POPULATION 60
3. RANDOMIZATION OF PARTICIPANTS 60
4. STUDY OBJECTIVES 60
4.1 Primary Study Objective 60
4.2 Secondary Study Objectives 61
4.3 Additional Objectives 61
5. SAMPLE SIZE JUSTIFICATION AND POWER CONSIDERATIONS 61
6. COMPLIANCE WITH STUDY PROCEDURES 62
6.1 Violations of Inclusion/Exclusion Criteria 62
6.2 Adherence to Assigned Treatment Strategy 62
6.2.1 Receipt of traditional cardiac rehab (CR) 62
6.2.2 Engagement with mHealth-CR 62
7. GENERAL STRATEGY FOR PRIMARY AND SECONDARY ENDPOINTS ANALYSIS 62
7.1 General 62
7.2 Primary Endpoint Analysis 63
7.3 Additional Supportive Analyses for the Primary Endpoint 63
7.3.1 Bayesian analysis of study endpoints 64
7.3.2 Plan for per-protocol analysis accounting for differential engagement with ambulatory cardiac rehab 64
7.4 Secondary Endpoint Analysis 64
7.5 Planned Subgroup Analyses to Assess Heterogenous Treatment Effect 65
7.6 Engagement Analysis 65
8. SAFETY ANALYSIS 66
9. AMMENDMENTS TO STATISTICAL PLAN 66
10. REFERENCES 66

1. INTRODUCTION

This statistical analysis plan (SAP) incorporates all of the elements of study design, statistical monitoring, and analysis of primary and secondary endpoints for RESILIENT trial. This SAP is a standalone section that describes the statistical design and analysis considerations for this randomized clinical trial.

2. STUDY OVERVIEW AND PATIENT POPULATION

RESILIENT is a phase II, multicenter, prospective, pragmatic randomized clinical trial (RCT). Participants with ischemic heart disease (IHD) age ≥65 years will be identified at the time of acute myocardial infarction (AMI) or coronary revascularization (percutaneous coronary intervention [PCI] or coronary artery bypass grafting [CABG]), and will be randomized in a 3:1 ratio to a mobile health cardiac rehabilitation program (mHealth-CR) or to usual care. We will randomize 400 participants. The initial sites chosen were the NYU School of Medicine / NYU Langone Health System (inclusive of NYU main campus, Bellevue Hospital, and NYU Long Island), and Yale New Haven Health. The Yale was discontinued on 02/08/2021 due to low enrollment, and a new site (University of Massachusetts) was added to the trial on 05/05/2021.

The main comparisons between randomized groups in this trial will be performed according to the principle of "intention-to-treat"; that is, participants will be analyzed according to the treatment group to which they were randomized, regardless of subsequent crossover. The intention-to-treat (ITT) population will include all subjects who receive a randomized treatment assignment, except for those with a documented violation of trial eligibility criteria.

3. RANDOMIZATION OF PARTICIPANTS

After providing informed consent and completing the baseline assessment, enrolled participants will be randomized via Research Electronic Data Capture (REDCap) in a 3:1 ratio to either intervention (mHealth-CR) or control (usual care), using permuted block randomization created by the trial statistician (Dr. Adhikari), with variable block sizes of 4 and 8. The rationale for 3:1 randomization is to efficiently address both the primary comparison of mHealth-CR and usual care with respect to the 6MWD and to allow an adequate sample size to estimate engagement trajectories (Aim 5). The proposed 3:1 randomization enables a 50% increase in the sample size for Aim 3, while also providing excellent power for the primary hypothesis test. Randomization is be stratified by hospital (NYU Tisch, NYU Bellevue, NYU Long Island, Yale New Haven, University of Massachusetts) to ensure balance across treatment arms given between-hospital population differences.

4. STUDY OBJECTIVES

4.1 Primary Study Objective

The study includes objectives related to both efficacy and engagement. The *primary efficacy objective* is to determine whether mHealth-CR improves functional capacity as assessed by 6 Minute Walk Distance (6MWD), compared with usual care.

Primary hypothesis: mHealth-CR will improve functional capacity as measured by change in 6MWD between intervention and control arms at 3 months.

4.2 Secondary Study Objectives

The *secondary efficacy objectives* are to evaluate whether mHealth-CR improves the endpoints of (1) goal attainment, as measured by goal attainment scaling (GAS); (2) health status (general and disease-specific); (3) basic and instrumental activities of daily living (ADLs); (4) hospital readmission; and (5) death, in participants receiving mHealth-CR versus usual care. Endpoints (1) through (3) are evaluated at 3 months, while endpoints (4) and (5) are evaluated at 12 months.

4.3 Additional Objectives

The *ancillary efficacy objectives* are to assess whether mHealth-CR improves the quantitative endpoints of (1) mean systolic blood pressure at 3-month follow-up visit; (2) change in frailty status; (3) attendance rate at traditional (ambulatory) CR; (4) change in PHQ-8 depression score (baseline to 3 months). An additional ancillary objective is qualitative, and involves identifying barriers to and facilitators of participation using interviews in a subsample of participants.

The *primary engagement objective* is to characterize engagement of participants with mHealth-CR and elucidate barriers to participation.

5. SAMPLE SIZE JUSTIFICATION AND POWER CONSIDERATIONS

We will randomize 400 participants using a 3:1 allocation, resulting in a projected 300 participants in the intervention group and 100 participants in the usual care group. While we will aim to minimize participant dropout, we include adjustment for up to 20% dropout in each arm, resulting in about 320 participants with evaluable endpoints (240 intervention, 80 usual care). We wish to be able to detect a clinically meaningful difference between groups in our primary efficacy endpoint, change in 6MWD from baseline to 3 months. A recent meta-analysis [1] found an average difference in 6MWD of about 60 meters before and after traditional CR. Perera et al. determined this degree of change in 6MWD to be clinically meaningful based on its relationship to other health status measures.[2] Minneboo et al., however, also estimated an improvement in 6MWD in a control group of approximately 35 meters.[3] We therefore require adequate power to detect a difference between groups of less than 25 meters in change in 6MWD (i.e., the difference between a 60-meter improvement in the intervention group and a 35-meter improvement in the usual care group); this is the same amount estimated by Gremeaux et al. as a minimal clinically important difference in patients with IHD.[4] Assuming a conservative standard deviation estimate of 60 meters,[1] 320 participants provide approximately 90% power to detect a difference between groups of 25 meters, using a two-sided, 0.05-level test; there is 80% power to detect a difference between groups as small as 22 meters. This sample size also provides at least 80% power to detect reasonable effect sizes in secondary endpoints.

While the sample size was determined to provide sufficient power to detect a clinically meaningful difference in the primary outcome of 6MWD, it enables comparisons of secondary efficacy endpoints as well. The table below shows clinically meaningful effect sizes for these outcomes, and gives the power to detect these effect sizes with our expected sample of 320 patients, using two-sided, 0.05-level tests; we will not implement multiple comparisons correction for these secondary outcomes. Baseline event rates and standard deviations are estimated form the literature.[5-8] For example, we have 90% power to detect a difference between groups of 5 units in the SF-12 score.

Outcome	Endpoint definition	Expected difference	Estimated sd	Power
Goal attainment scaling	GAS score	5	15	73%
Health status	SF-12	5	12	90%
Health status	Residual angina on SAQ	15-20%	n/a	67-93%
ADLs	Standardized ADL score	15-20%	1	64-87%
Hospital readmission	Admission by 12 months	15%	n/a	91%
Death	Death by 12 months	10%	n/a	82%

6. COMPLIANCE WITH STUDY PROCEDURES

6.1 Violations of Inclusion/Exclusion Criteria

The number of patients with violations of the inclusion/exclusion criteria will be presented. Completion of follow-up visits. Summaries of study visit completion and timeliness will be created and displayed by site.

6.2 Adherence to Assigned Treatment Strategy

6.2.1 Receipt of traditional cardiac rehab (CR)

Participants in both arms will receive information about traditional (ambulatory) CR, which is the standard of care for patients with ischemic heart disease (either following hospitalization for AMI or a coronary revascularization procedure). In the interest of a pragmatic trial, referral to traditional CR is not mandated as part of the study protocol, but is left to the discretion of the outpatient cardiologist. We will summarize the referral rate as well as the proportion of those referred who attend traditional CR.

6.2.2 Engagement with mHealth-CR

The mHealth-CR intervention includes a number of activities expected of participants randomized to this arm. The primary engagement endpoint is an 11-point score, tabulated weekly, indicating completion of each component of the mHealth-CR intervention: (1–7) daily entry of exercise data and rated perceived exertion (1 point for each day with total possible 7 points per week); (8) completed phone call with exercise therapist; (9) at least one electronic communication with exercise therapist; (10) watching educational video; (11) at least one home BP measurement. Engagement will be assessed as a pseudo-continuous outcome, as the score can range from 0% (0 activities completed) to 100% (all activities completed).

7. GENERAL STRATEGY FOR PRIMARY AND SECONDARY ENDPOINTS ANALYSIS

7.1 General

Statistical comparisons will be performed using two-sided significance tests and two-sided confidence intervals. We will begin all analyses with descriptive summary statistics and graphical displays of all variables, with attention to assessing balance in these characteristics by intervention group, and with

assessment of the distribution of variables, relevant to the choice of statistical tests.

Rules for missing values will be discussed in appropriate places in the analysis plan below. In general, all available data will be included in data listings and tabulations. Population denominators will be displayed in column headers. Individual denominators will be displayed for each summary such that the number of missing values will be evident. Patients who withdraw from the study or are lost to follow-up will still be included in the denominators for any proportions where data are available.

We will use covariate adjusted analysis for primary and secondary endpoints, adjusting for prognostic covariates and sites. The prognostic baseline variables will be informed by the literature as predictors of the primary and secondary endpoints. We will use linear regression for continuous outcomes and logistic regression for binary outcomes. We will report effect estimates as difference in mean or difference in proportion with 95% confidence intervals.

7.2 Primary Endpoint Analysis

The primary endpoint of this study is the change in the 6MWD from baseline to 3 months. For primary endpoint analysis we will use a linear regression model. We will regress 3-month 6MWD on a binary indicator of treatment group, with adjustment for baseline 6MWD, the stratification factor (enrollment site), age, and presence of diabetes. The use of prognostic baseline covariates for adjustment can improve efficiency for estimating and testing treatment effects in RCTs. [9]

The primary analysis will be based on the principle of intention to treat (ITT); participants randomized to receive the study intervention will be considered as receiving mHealth-CR even if they failed to reliably engage with the platform. If there are differential dropouts or missingness for the primary endpoint by treatment arm, we will incorporate imputation as part of the primary analysis. To account for missing endpoints we will impute the corresponding outcomes and baseline factors among all randomized participants (ITT sample).

To assess whether there is differential missingness based on baseline covariates (evaluation of missing at random assumption), we will first summarize baseline covariates among those with and without missingness. The assumption of missing at random or MAR implies that the missing data are independent of unobserved factors conditional on observed data. We will next use multiple imputation using chained equations (MICE) for imputation, with all baseline variables and treatment arm included in the imputation model. Multiple imputation is a statistical technique to sequentially estimate a set of plausible values for the missing data using the observed data distribution.[10] First, we will replace missing values for m independent imputed data. We will use predictive mean matching for continuous variables and logistic regression to impute missing binary variables. One we have imputed m different copies of the data, for each imputed copy, we will assess the 6MWD using the regression approach described above to estimate the difference in 3-month 6MWD between the two arms. Finally, we will combine the results from all m imputed datasets using Rubin's rule to account for imputation uncertainty.

Based on the published recommendations, we will consider all variables (including the primary and secondary outcomes and planned interaction effects) that are part of the analysis in the imputation model to avoid bias. Further, the number of imputations (m) are based on fraction of missing information which is often approximated by the percent of missing data. Consistent with the rule of thumb, we will set m to be greater than or equal to the percent of cases that are missing. The package "MICE" within statistical software R will be used for the imputation.

7.3 Additional Supportive Analyses for the Primary Endpoint

7.3.1 Bayesian analysis of study endpoints

We will conduct supportive analyses using the Bayesian framework to assess posterior predictive distribution of the difference in 6-minute walk distance change between the two arms. We will assign prior distribution to the model parameters and sample from the posterior conditional on the data and prior using a Markov Chain Monte Carlo (MCMC) approach. We will summarize the median and 95% credible interval of the posterior difference in mean. Further, we will compute the probability that the posterior difference is above 25 meters change in 6-minute walk distance.

In the Bayesian model specification, we will assign independent non-informative normal prior distributions with mean 0 and a diffuse variance of 100 for the regression coefficients. We will conduct sensitivity analyses according to different choice of prior distributions. To fit the Bayesian models, we will use three MCMC chains after appropriate burn-in and thinning from dispersed initial values and assess model convergence visually based on trace plots, and using the Gelman-Rubin convergence diagnostic.[11]

7.3.2 Plan for per-protocol analysis accounting for differential engagement with ambulatory cardiac rehab

We realize that engagement with the mHealth-CR program may also affect attendance and engagement with traditional (ambulatory) CR programs post randomization. Therefore, to adjust for any potential post randomization confounding due to differential attendance at ambulatory CR programs, we will conduct a sensitivity analysis adjusting for inverse probability weights.[12-15] First, we will estimate the propensity of attending traditional ambulatory CR programs for each participant by fitting a logistic regression model, separately for each arm. The models will include time since randomization (in days) as well as baseline covariates. We will standardize the inverse probability weights constructed from these models for stability. We will then proceed with the outcome analysis by applying the inverse probability weights in the regression analysis. Bootstrapped 95% confidence intervals along with estimate of mean will be computed. We also acknowledge that patients in the treatment arm may have varying level of engagement with the m-health CR intervention. To account for differential adherence to treatment, we will apply appropriate causal inference strategies for estimating per-protocol effects.[16]

7.4 Secondary Endpoint Analysis

Protocol-defined secondary endpoints are: (1) goal attainment, as measured by goal attainment scaling (GAS); (2) change in participant reported health status: general (SF-12) and disease specific (SAQ-7); (3) change in activities of daily living (ADLs); (4) hospital readmission; and (5) death, in participants receiving mHealth-CR versus usual care. Analysis of secondary efficacy endpoints will proceed in a similar fashion as primary endpoint analysis. As with the models for the primary outcome, we will consider multiple imputation for the analysis of all secondary endpoints. Supportive analyses described for the primary endpoint will also be considered for the secondary endpoints as appropriate.

Ancillary endpoints are (1) change in mean systolic blood pressure at 3-month follow-up visit; (2) change in frailty status; (3) attendance rate at traditional (ambulatory) CR; (4) change in PHQ-8 depression score (baseline to 3 months).

An additional ancillary objective is qualitative, and involves identifying barriers to and facilitators of participation using interviews in a subsample of participants.

Goal attainment will be assessed at baseline and 3 months. At baseline, participants will set 1 personal goal related to improving daily function or symptom control (e.g., walking with less dyspnea, attending

family functions). At 3 months, goal attainment will be rated using a 5-point GAS, scored as follows: no change from baseline (-2); less than expected (-1), expected (0), better than expected (+1), and much better than expected (+2). To preserve the ordinal nature of the data, we will calculate a median GAS score (-2 to +2) for each participant, and then compare the treatment and control groups using a Wilcoxon-Mann-Whitney rank-sum test.[17] Secondly, we will transform each participant's GAS score into a single aggregated T-score using a simplified formula for one-goal GAS: GAS=50+(10x), where x is the numerical value (-2 to $+2$) of the attainment level. A T-score of 50 indicates that on average the expected level of goal attainment was achieved.[18] We will then compare mean T-scores for treatment and control groups at 3 months using a two-sample t-test. We will also compare the percentage of participants in the treatment and control groups who met their expected level of goal attainment (dichotomized as GAS score 0,+1,+2 vs. -1,-2) using a chi-square test.[19]

Health status at 3 months will be assessed using linear regression with adjustment for baseline levels and a binary treatment indicator; if necessary, health status scores will be log-transformed to improve the approximation to normality. Activities of daily living (Basic ADLs and Instrumental ADLs) will be assessed using longitudinal models for weekly scores, with indicators to incorporate time, a binary indicator of treatment group, and patient-level random effects to accommodate repeated assessments within individuals; if weekly scores are not approximately normally distributed, suitable transformations will be sought.

Hospital readmission and death will be evaluated using Kaplan-Meier estimates and tested with logrank statistics, as well as investigated using Cox proportional hazard models with adjustment for confounders if necessary.

7.5 Planned Subgroup Analyses to Assess Heterogenous Treatment Effect

In order to understand treatment effect heterogeneity, we will perform exploratory analyses to estimate treatment effects within each of subgroup for our primary and secondary endpoints. The prespecified subgroups will include (1) age 65-74 vs. ≥75, (2) sex, (3) body mass index (<25 vs. 25-30 vs. ≥30), (4) diabetes, (5) heart failure, (6) osteoarthritis, (7) chronic lung disease, (8) chronic kidney disease (GFR <60), (9) frailty status (frail vs. prefrail vs. robust), (10) disability (defined as any ADL or IADL impairment), (11) depression (PHQ-8 ≥10), (12) living alone, (13) indication for CR (AMI vs. CABG vs. elective PCI).

7.6 Engagement Analysis

We hypothesize that (1) among participants offered mHealth-CR, there will be distinct trajectories of engagement (e.g., sustained engagement, disengagement, reengagement, deteriorating engagement); and (2) there will be significant differences in participant characteristics among these groups. While our Aim is largely exploratory (given the paucity of data on mHealth-CR engagement), we will test whether the following characteristics are significant: age (≥80 years), sex, race/ethnicity, comorbidity burden (≥2 chronic medical conditions), frailty, social support (based on living alone), and depressive symptoms (based on PHQ-8). We selected these factors based on literature related to engagement in other technologies. We will conduct latent class analysis to identify profiles of engagement and explore whether these factors indicate membership in a class; these models use maximum likelihood estimation, implemented with the iterative EM algorithm, to identify a latent class solution for the set of indicators. We will evaluate model fit using the G^2 statistic and compare models with the likelihood-difference test for nested models and the Akaike and Bayesian information criteria (AIC, BIC) for non-nested models. While we have identified four potential classes a priori, we will use the parametric bootstrap likelihood ratio test to select the optimal number of classes supported by the data in conjunction with the AIC and

BIC. The output of the model will be a set of "item-response" probabilities giving the likelihood of a particular characteristic within each latent class, and a set of posterior predicted probabilities of latent class membership; uncertainty in predicting class membership will be summarized using the odds of correct classification (OCC) diagnostic tool. We will use R package 'lcmm' for the latent class analysis.[20]

To elucidate barriers to participation, we will use procedures recommended by Patton and others that focus on developing coding protocols to highlight issues, problems, and potential recommendations.[21] The goals of these analyses are to identify key barriers and facilitators, as well as any emerging themes related to the use of mHealth-CR to improve functional recovery and other patient-centered outcomes. Focus groups and interviews will employ semi-structured interview guides. Two independent viewers will record, transcribe, and code sessions. Coding will involve breaking responses into discrete units (e.g., sentences, phrases) and categorizing concepts into thematic groups. Discrepancies in coding will be resolved by reaching consensus among two investigators.

Among participants randomized to the intervention arm, we will also analyze exercise characteristics including Fitbit data (e.g. step count, percent time in activity categories) and data entered by participants into the Moving Analytics platform (e.g. minutes of exercise, type of exercise). These data will provide an estimate of the overall weekly activity level among these individuals.

8. SAFETY ANALYSIS

Adverse events and severe adverse events as specified in the study protocol will be tabulated and presented overall and by treatment arm.

9. AMMENDMENTS TO STATISTICAL PLAN

The statistical analysis plan will be finalized based on the final protocol approved for implementation by the study enrolling centers. This plan will be amended as the study proceeds to document all changes to data conventions and operational decisions that are implemented during the trial. The amended plan will include the analysis approaches to the evaluation of the impacts of all changes to the study design and implementation. No changes to the SAP will occur after database lock and unmasking of patient data. The SAP will be reviewed with the PI and study investigators.

10. REFERENCES

1. Bellet, R. N., Adams, L., & Morris, N. R. (2012). The 6-minute walk test in outpatient cardiac rehabilitation: validity, reliability and responsiveness--a systematic review. Physiotherapy, 98(4), 277–286. https://doi.org/10.1016/j.physio.2011.11.003
2. Perera, S., Mody, S. H., Woodman, R. C., & Studenski, S. A. (2006). Meaningful change and responsiveness in common physical performance measures in older adults. Journal of the American Geriatrics Society, 54(5), 743–749. https://doi.org/10.1111/j.1532-5415.2006.00701.x
3. Minneboo, M., Lachman, S., Snaterse, M., Jørstad, H. T., Ter Riet, G., Boekholdt, S. M., Scholte Op Reimer, W. J. M., Peters, R. J. G., & RESPONSE-2 Study Group (2017). Community-Based Lifestyle Intervention in Patients With Coronary Artery Disease: The RESPONSE-2 Trial. Journal of the American College of Cardiology, 70(3), 318–327. https://doi.org/10.1016/j.jacc.2017.05.041
4. Gremeaux, V., Troisgros, O., Benaïm, S., Hannequin, A., Laurent, Y., Casillas, J. M., & Benaïm, C. (2011). Determining the minimal clinically important difference for the six-minute walk test and the 200-meter fast-walk test during cardiac rehabilitation program in coronary artery disease

patients after acute coronary syndrome. Archives of physical medicine and rehabilitation, 92(4), 611–619. https://doi.org/10.1016/j.apmr.2010.11.023

5. Dharmarajan, K., Hsieh, A., Dreyer, R. P., Welsh, J., Qin, L., & Krumholz, H. M. (2017). Relationship Between Age and Trajectories of Rehospitalization Risk in Older Adults. Journal of the American Geriatrics Society, 65(2), 421–426. https://doi.org/10.1111/jgs.14583
6. Arnold, S. V., Jang, J. S., Tang, F., Graham, G., Cohen, D. J., & Spertus, J. A. (2015). Prediction of residual angina after percutaneous coronary intervention. European heart journal. Quality of care & clinical outcomes, 1(1), 23–30. https://doi.org/10.1093/ehjqcco/qcv010
7. Grodzinsky, A., Arnold, S. V., Gosch, K., Spertus, J. A., Foody, J. M., Beltrame, J., Maddox, T. M., Parashar, S., & Kosiborod, M. (2015). Angina Frequency After Acute Myocardial Infarction In Patients Without Obstructive Coronary Artery Disease. European heart journal. Quality of care & clinical outcomes, 1(2), 92–99. https://doi.org/10.1093/ehjqcco/qcv014
8. Dolovich, L., Oliver, D., Lamarche, L., Agarwal, G., Carr, T., Chan, D., Cleghorn, L., Griffith, L., Javadi, D., Kastner, M., Longaphy, J., Mangin, D., Papaioannou, A., Ploeg, J., Raina, P., Richardson, J., Risdon, C., Santaguida, P. L., Straus, S., Thabane, L., … Price, D. (2016). A protocol for a pragmatic randomized controlled trial using the Health Teams Advancing Patient Experience: Strengthening Quality (Health TAPESTRY) platform approach to promote person-focused primary healthcare for older adults. Implementation science : IS, 11, 49. https://doi.org/10.1186/s13012-016-0407-5
9. Ye, T., Shao, J., Yi, Y., & Zhao, Q. (2023). Toward Better Practice of Covariate Adjustment in Analyzing Randomized Clinical Trials. Journal of the American Statistical Association, 118(544), 2370–2382. https://doi.org/10.1080/01621459.2022.2049278
10. White, I. R., Royston, P., & Wood, A. M. (2011). Multiple imputation using chained equations: Issues and guidance for practice. Statistics in medicine, 30(4), 377–399. https://doi.org/10.1002/sim.4067
11. Gelman, A. (2004). Bayesian data analysis. 2nd ed. Texts in statistical science. Boca Raton, Fla.: Chapman & Hall/CRC. xxv, 668 p
12. Robins, J. M., Blevins, D., Ritter, G., & Wulfsohn, M. (1992). G-estimation of the effect of prophylaxis therapy for Pneumocystis carinii pneumonia on the survival of AIDS patients. Epidemiology (Cambridge, Mass.), 3(4), 319–336. https://doi.org/10.1097/00001648-199207000-00007
13. Robins, J. M., & Finkelstein, D. M. (2000). Correcting for noncompliance and dependent censoring in an AIDS Clinical Trial with inverse probability of censoring weighted (IPCW) log-rank tests. Biometrics, 56(3), 779–788. https://doi.org/10.1111/j.0006-341x.2000.00779.x
14. Robins, J. M., & Tsiatis, A. A. (1991). Correcting for non-compliance in randomized trials using rank preserving structural failure time models. Communications in Statistics - Theory and Methods, 20(8), 2609–2631. https://doi.org/10.1080/03610929108830654
15. Zhang, M., Tsiatis, A. A., Davidian, M., Pieper, K. S., & Mahaffey, K. W. (2011). Inference on treatment effects from a randomized clinical trial in the presence of premature treatment discontinuation: the SYNERGY trial. Biostatistics (Oxford, England), 12(2), 258–269. https://doi.org/10.1093/biostatistics/kxq054
16. Hernán, M. A., & Robins, J. M. (2017). Per-Protocol Analyses of Pragmatic Trials. The New England journal of medicine, 377(14), 1391–1398. https://doi.org/10.1056/NEJMsm1605385
17. Turner-Stokes L. (2009). Goal attainment scaling (GAS) in rehabilitation: a practical guide. Clinical rehabilitation, 23(4), 362–370. https://doi.org/10.1177/0269215508101742
18. Krasny-Pacini, A., Hiebel, J., Pauly, F., Godon, S., & Chevignard, M. (2013). Goal attainment scaling in rehabilitation: a literature-based update. Annals of physical and rehabilitation medicine, 56(3), 212–230. https://doi.org/10.1016/j.rehab.2013.02.002
19. Proust-Lima, C., Philipps, V., & Liquet, B. (2017). Estimation of Extended Mixed Models Using Latent Classes and Latent Processes: The R Package lcmm. Journal of Statistical Software, 78(2), 1–56. https://doi.org/10.18637/jss.v078.i02

20. McGarrigle, L., & Rockwood, K. (2020). The responsiveness of goal attainment scaling using just one goal in controlled clinical trials: an exploratory analysis. Journal of patient-reported outcomes, 4(1), 35. https://doi.org/10.1186/s41687-020-00196-8
21. Patton MQ. Qualitative evaluation and research methods. 2nd ed. Newbury Park, CA: Sage; 2002
