# Supplementary material for: Rehabilitation at Home Using Mobile Health for Older Adults Hospitalized for Ischemic Heart Disease: The RESILIENT Randomized Clinical Trial
Source: JAMA Netw Open. Author manuscript; Available in PMC 2025 Jul 11. (PMC12247035; doi:10.1001/jamanetworkopen.2024.53499)
Supplement: Supplement 2 — eAppendix 1. List of Study Sites for the RESILIENT Trial and Adverse Event Reporting eMethods eAppendix 2. Additional Statistical Analysis Details eTable 1. Characteristics of Participants With Completed vs Missing Measurement of Primary End Point at 3 Months eTable 2. Missing End Point Data by Study Site eTable 3. Analysis by Receipt of Any CR eTable 4. Adverse Events eFigure 1. Sample Layout of mHealth-CR Software eFigure 2. Results of Bayesian Analysis eFigure 3. mHealth-CR Engagement Among Intervention Arm Participants [file NIHMS2086641-supplement-Supplement_2.pdf]

Supplementary Online Content

Dodson JA, Adhikari S, Schoenthaler A, et al. Rehabilitation at home using mobile health for older adults hospitalized for ischemic heart disease: the RESILIENT randomized clinical trial. *JAMA Netw Open*. 2025;8(1):e2453261. doi:10.1001/jamanetworkopen.2024.53261

eAppendix 1. List of Study Sites for the RESILIENT Trial and Adverse Event Reporting
eMethods
eAppendix 2. Additional Statistical Analysis Details
eTable 1. Characteristics of Participants With Completed vs Missing Measurement of Primary End Point at 3 Months
eTable 2. Missing End Point Data by Study Site
eTable 3. Analysis by Receipt of Any CR
eTable 4. Adverse Events
eFigure 1. Sample Layout of mHealth-CR Software
eFigure 2. Results of Bayesian Analysis
eFigure 3. mHealth-CR Engagement Among Intervention Arm Participants

This supplementary material has been provided by the authors to give readers additional information about their work.

© 2025 Dodson JA et al. *JAMA Network Open*.

eAppendix 1

List of study sites for the RESILIENT trial

Study Site	Location	Site Principal Investigator(s)	Number of participants enrolled
NYU Langone - Main Campus	New York, NY	John Dodson, MD	210
NYU Langone – Long Island	Mineola, NY	Barbara George, EdD<br>Kevin Marzo, MD	52
Bellevue Hospital	New York, NY	Mathew Vorsanger, MD	29
University of Massachusetts	Worcester, MA	Laura Kovell, MD	107
Yale University*	New Haven, CT	Sarwat Chaudhry, MD	2
*Yale site was discontinued 02/08/2021 due to low enrollment.			

Adverse event reporting

Data safety monitoring board (DSMB) members:
Hank Wu, MD, MPH, *Chair* (Brown University)
Pamela Peterson, MD, MSPH (University of Colorado Denver)
Chris C. Cho, MS (University of Wisconsin)

NIH (NIA) Program Official: Sergei Romashkan, MD

Definition of Adverse Events:
An adverse event (AE) is any symptom, sign, illness or experience that develops or worsens in severity during the course of the study. Intercurrent illnesses or injuries should be regarded as adverse events. Abnormal results of diagnostic procedures are considered to be adverse events if the abnormality. Adverse events can be classified as serious or non-serious. A serious adverse event is any AE that is:

- fatal
- life-threatening
- requires or prolongs hospital stay
- results in persistent or significant disability or incapacity
- a congenital anomaly or birth defect
- an important medical event

Adverse event reporting
Adverse events are reported to the NYU SoM IRB, the NIH Program Officer, and the study's DSMB as appropriate (i.e. relationship to study intervention, severity). The Principal Investigators inform fellow investigators and study personnel, via email, of all adverse events that occur during the conduct of this research project as they are reviewed. Adverse events and serious adverse events occurring during the active period of the study (3 months after baseline visit) are be monitored and reported to DSMB for all study subjects.

To prevent a discrepancy between randomization groups, monthly chart reviews of active participants in both arms are screened for AEs and SAEs. Other ways of discovering AEs and SAEs are speaking with the

© 2025 Dodson JA et al. *JAMA Network Open*.

subjects during the research coordinator phone calls, learning about these events through the interventionists, or learning about the event by receiving patient chart alert.

Once the study team records an adverse event using the NIH template and REDcap form, it is adjudicated by the site PI (a physician) in regards to the severity, relationship to intervention, actions taken, outcome, expected and if serious or non-serious. If the adverse event is classified as serious, another NIH template is filled out. For unanticipated serious events that are possibly or definitely related to study procedures, the investigator submits the form to the NIA Program Officer and to the DSMB Chair and DSMB members within 48 hours of study team being notified of the event. The investigator follows all reportable events until resolution or stabilization.

In the event of participant deaths related to the study, in compliance with NIA requirements for investigators conducting human intervention studies, all deaths require expedited reporting (approximately 24 hours after the study team is notified of the death). This report is be submitted to the NIA Program Officer and to the DSMB Chair or designated DSMB member.

© 2025 Dodson JA et al. *JAMA Network Open*.

eMethods

Eligibility criteria

Inclusion:

- Age ≥65 years
- Hospital visit for acute myocardial infarction and/or coronary revascularization (PCI or CABG)

Exclusion:

- Non-ambulatory or regular use of walker for ambulation
- Moderate or severe cognitive impairment (defined as cognitive impairment that interferes with daily function)
- Unable/unwilling to consent
- PCI-related groin hematoma that precludes brisk walking
- Incarcerated
- Unable to use mHealth-CR software in English or Spanish
- Severe osteoarthritis, or joint replacement within last 3 months
- Parkinson's disease or other progressive movement disorder
- Projected life expectancy <3 months
- Clinical judgment concerning other safety or nonadherence issues
- Adverse event during screening 6 minute walk test (e.g. drop in SBP ≥15 mmHg, severe angina, ventricular arrhythmia, syncope)

© 2025 Dodson JA et al. *JAMA Network Open*.

Study schematic

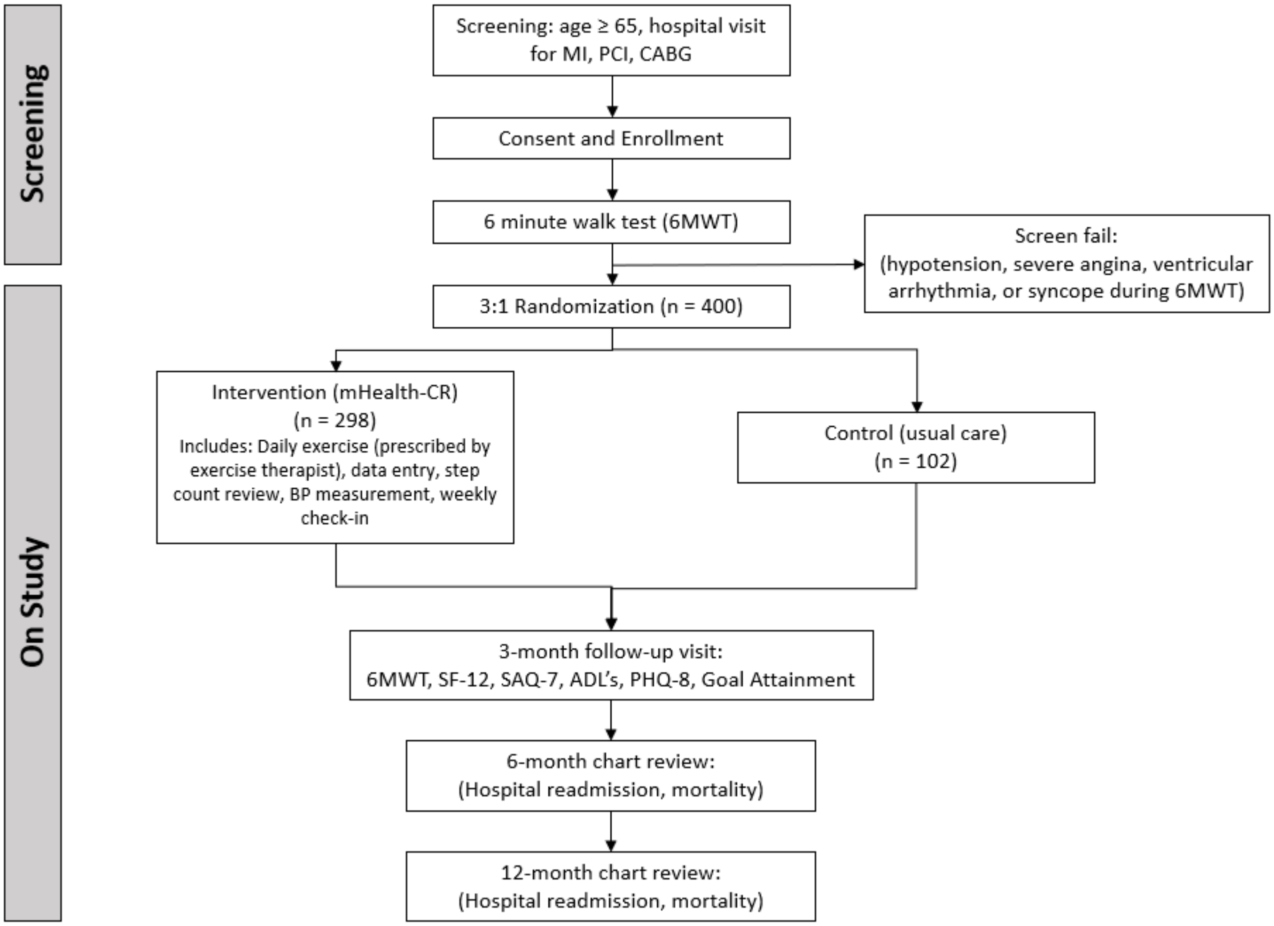

© 2025 Dodson JA et al. *JAMA Network Open*.

Schedule of study activities

	Baseline	Home activities	3 months
Intervention and usual care arms[A]	In-person assessment<br>• Demographics<br>• Height, weight, blood pressure<br>• 6 minute walk test[B]<br>• Health status (SF-12, SAQ-7)<br>• Activities of Daily Living (ADLs), Instrumental Activities of Daily Living (IADLs)<br>• Cognition (MiniCog)<br>• Goal attainment scaling (GAS)<br>• Depression (PHQ-8)<br>• Frailty elements[C]<br>Chart abstraction<br>• Comorbidities, medications, laboratory values	• Monthly ADLs/IADLs assessment (telephone)	In-person assessment<br>• Weight, blood pressure<br>• 6 minute walk test<br>• Health Status (SF-12, SAQ-7)<br>• ADLs/IADLs<br>• Goal attainment scaling (GAS)<br>• Depression (PHQ-8)<br>• Frailty elements<br>• Hospital readmissions<br>Chart abstraction<br>• Hospital readmission (verification)[D], attendance at traditional CR
Intervention arm	Exercise therapist assessment<br>• Education on cardiac risk factor management<br>• Ascertainment of home environment/mobility barriers<br>• Introduction to mHealth-CR software platform<br>• Personalized exercise plan	• Daily therapist-directed activity (walking, upper extremity resistance training)<br>• Daily mHealth data entry<br>• Weekly therapist phone call (counseling/activity review)<br>• Weekly video education<br>• Weekly blood pressure<br>• Fitbit activity tracking and review	• System Usability Scale

Abbreviations: SF-12 = Short Form 12; SAQ-7 = Seattle Angina Questionnaire 7

[A] Intervention and usual care participants also receive information about traditional (ambulatory) CR at hospital discharge, but referral is at the discretion of their outpatient cardiologist.

[B] 6 minute walk test is performed by an exercise therapist or research nurse who is blinded to treatment assignment.

[C] Based on 3/5 criteria: unintentional weight loss, weak grip strength (dynamometer), exhaustion, slow gait, low physical activity.

[D] Hospital readmission is also be ascertained at 6 and 12 months through electronic health record review

© 2025 Dodson JA et al. *JAMA Network Open*.

Measurement of primary endpoint (change in 6 minute walk distance)

Chance in 6 minute walk distance is calculated based on the 6 minute walk test (6MWT). The 6MWT is a submaximal exercise test at a self-selected pace. To minimize bias and ensure safety, 6MWT is performed by a blinded research nurse or blinded exercise therapist who is unaffiliated with the study intervention. The 6MWT is performed during baseline study visit, and at the 3-month follow-up visit. The protocol is based on recommendations from the American Thoracic Society (*Am J Respir Crit Care Med* 2002; 166: 111–117).

A 6MWT script and directions sheet is used to administer the 6MWT which outlines that the patient will walk as far as possible for 6 minutes without running or jogging. A walking course of at least 50 feet is used when feasible, and the test is performed at a clinical location. Vital signs (SpO2 levels, heart rate and blood pressure) are taken prior to starting the 6MWT, immediately after the 6MWT is completed, and 5 mins after rest from the 6MWT. The Borg Exertion Scale is also administered. Once the 6MWT has been completed, the blinded assessor signs off on the 6MWT worksheet. Randomization occurs following completion of the 6MWT protocol. Concerning signs or symptoms (systolic blood pressure decline ≥15 mmHg, severe angina, unstable arrhythmia, or syncope) during 6MWT constitute a “screen fail” and patients who experience these issues are not randomized, in order to minimize the risk of subsequent events during mHealth-CR. In the event of a screen fail, the patient’s cardiologist is notified. For participants who are randomized to receive mHealth-CR, results from the 6MWT are made available to the study exercise therapist, in order to help inform the exercise prescription.

© 2025 Dodson JA et al. *JAMA Network Open*.

eAppendix 2. Additional Statistical Analysis Details

Details on multiple imputation

We used multiple imputation using chained equations (MICE) for imputation of missing values (baseline covariates and outcomes). Multiple imputation is a robust statistical technique to sequentially estimate a set of plausible values for the missing data using the observed data distribution. Because missingness in the primary outcome (6MWD) differed by treatment arm, and because there was differential missingness as a function of baseline covariates, we inferred that missing at random was a more plausible assumption than missing completely at random, and therefore decided to use MICE for imputation as opposed to complete case analysis.

In addition to the outcomes measured during follow-up, we included all observed baseline variables (demographics, comorbidities, frailty and treatment arm) in the imputation model. We used predictive mean matching for continuous variables and logistic regression to impute missing binary variables. As recommended by White (https://doi.org/10.1002/sim.4067), we set the number of imputations to 50 and maximum iterations for each imputation to 100. For each imputed copy, we assessed the 6MWD using the covariate-adjusted regression approach to estimate the difference in 3-month 6MWD between the two treatment arms. Finally, we combined the results from all 50 imputed datasets using Rubin's rule to account for imputation uncertainty. A similar approach was used for secondary outcomes analysis. The package "MICE" within statistical software R was used for the imputation.

Details on estimation of average controlled direct effect

We have performed a post hoc analysis to estimate the average effect of the mHealth CR for patients attending traditional CR and the average effect for those not attending traditional CR. These types of causal effects are often called controlled direct effects in casual inference literature. Because participation to traditional CR was not randomized we estimated the propensity score for the traditional rehab attendance adjusting for treatment assignment, baseline 6MWD, age, sex and number of comorbidities. Logistic regression was used for estimating propensity score. We then used inverse probability weighting (IPW) and marginal structural model to estimate the average controlled direct effect (CDE) assuming patients attended traditional rehab and average CDE assuming patients did not attend traditional rehab post randomization (doi:10.1017/S0003055416000216). IPW weighted linear regression model with sandwich estimator for the variance was used for the marginal model. We believe that this analysis shows a substantial (though still not statistically significant) effect of mHeath CR among those who did not attend traditional CR that is greater than 25 meters.

© 2025 Dodson JA et al. *JAMA Network Open*.

eTable 1. Characteristics of participants with completed vs. missing measurement of primary end point (6 minute walk test [6MWT]) at 3 months

Characteristic	3 month 6MWT completed (N=356)	3 month 6MWT missing (N=44)
Median age, range (yr)	71.0 [65.0, 91.0]	72.5 [65.0, 89.0]
Male sex – no. (%)	263 (73.9%)	28 (63.6%)
Female sex – no. (%)	93 (26.1%)	16 (36.4%)
Race – no. (%)[a]		
Asian	16 (4.5%)	1 (2.3%)
Black	27 (7.6%)	9 (20.5%)
Multiple races or other	36 (10.1%)	8 (18.2%)
White	277 (77.8%)	26 (59.1%)
Hispanic ethnicity – no. (%)	31 (8.7%)	3 (6.8%)
Hypertension – no. (%)	297 (83.4%)	40 (90.9%)
Diabetes – no. (%)	114 (32.0%)	22 (50.0%)
Heart failure – no. (%)	42 (11.8%)	5 (11.4%)
Atrial fibrillation – no. (%)	41 (11.5%)	6 (13.6%)
Chronic lung disease – no. (%)	50 (14.0%)	3 (6.8%)
Median body mass index, range (kg/m^2)	27.4 [14.7, 46.8]	27.5 [18.8, 40.3]
Estimated glomerular filtration rate		
Mean – ml/min/1.73 m^2	77.2	70.6
Distribution – no./total no. (%)		
<30 ml/min/1.73 m^2	4 (1.1%)	2 (4.5%)
30-59 ml/min/1.73 m^2	53 (14.9%)	9 (20.5%)
≥60 ml/min/1.73 m^2	299 (84.0%)	33 (75.0%)
History of tobacco use (any) – no. (%)	86 (24.2%)	11 (25.0%)
ADL or IADL impairment – no. (%)	54 (15.2%)	19 (43.2%)
Frailty – no. (%)[b]		
Frail	37 (10.4%)	6 (13.6%)
Prefrail	194 (54.5%)	24 (54.5%)
Robust	103 (28.9%)	4 (9.1%)
Depressive symptoms – no. (%)[c]	40 (11.2%)	5 (11.4%)
Medication use at discharge – no. (%)		
Beta blocker	240 (67.4%)	32 (72.7%)
Calcium channel blocker	99 (27.8%)	12 (27.3%)
Long-acting nitrate	46 (12.9%)	3 (6.8%)
Aspirin	326 (91.6%)	39 (88.6%)
P2Y12 inhibitor	325 (91.3%)	42 (95.5%)
Statin	328 (92.1%)	41 (93.2%)
Enrollment criteria – no. (%)		
Acute coronary syndrome		
Acute myocardial infarction with PCI	84 (23.6%)	12 (27.3%)
Acute myocardial infarction without PCI	8 (2.2%)	1 (2.3%)
Unstable angina with PCI	19 (5.3%)	4 (9.1%)
Elective percutaneous coronary intervention	228 (64.0%)	26 (59.1%)
Coronary artery bypass graft	17 (4.8%)	1 (2.3%)

a Race categories based on self-identified category. “Other race” includes American Indian or Alaska Native, Native Hawaiian or Other Pacific Islander, or participants self-selecting “other” without further specification.
b Frailty defined using Fried criteria (19)
c Depressive symptoms based on PHQ-8 (18)

© 2025 Dodson JA et al. *JAMA Network Open*.

eTable 2. Missing end point data by study site

Site	Missing	Present	Total
Bellevue	7 (24.1%)	22 (75.9%)	29 (100.0%)
Tisch	30 (14.3%)	180 (85.7%)	210 (100.0%)
UMass	6 (5.6%)	101 (94.4%)	107 (100.0%)
Winthrop	1 (1.9%)	51 (98.1%)	52 (100.0%)
Yale	0 (0.0%)	2 (100.0%)	2 (100.0%)
Total	44 (11.0%)	356 (89.0%)	400 (100.0%)

© 2025 Dodson JA et al. *JAMA Network Open*.

eTable 3. Analysis by receipt of any CR

Outcome	Randomized to mHealth-CR (N=298) Mean Δ6MWD	Randomized to usual care (N=102) Mean Δ6MWD	Average controlled direct effect in Mean Δ6MWD Mean [95%CI] (b)
**Attended traditional CR (a)**	34.7 meters<br>(N=38)	43.5 meters<br>(N=26)	0.04 [-64.1, 65.2]
**Did not attend traditional CR**	42.2 meters<br>(N=260)	19.3 meters<br>(N=76)	25.7 [-8.7, 60.2]

(a) Indicates attendance of at least one traditional CR session during 3 month follow-up period. Mean number of traditional CR sessions attended among this population = 18 (range 1-36 sessions).

(b) Average controlled direct effect (CDE) estimates the propensity score weighted difference in 6MWD change between the two arms when patients attended traditional cardiac rehab and when patients did not attend traditional cardiac rehab. Because participation in traditional CR was not randomized, we estimated the propensity score for the traditional rehab attendance adjusting for treatment assignment, baseline 6MWD, age, sex and number of comorbidities.

© 2025 Dodson JA et al. *JAMA Network Open*.

eTable 4. Adverse events

Adverse Events through Week 12.			
**Event**	**Intervention (N=298)**	**Control (N=102)**	**All**
Adverse Event			
Any	64	18	82
Cardiac	24	11	35
Chest pain	12	2	14
Planned procedure	4	7	11
Other	8	2	10
Non-cardiac	40	7	47
Body pain	11	3	14
Fall	8	1	9
Other	21	3	24
Serious Adverse Event	19	4	23
Any	19	4	23
Hospitalization	19	4	23
Death	0	0	0
Cardiac	9	2	11
Chest pain, tightness, angina	2	0	2
Planned procedure	2	1	3
Heart failure	1	0	1
Myocardial infarction	0	1	1
Arrythmia, claudication, hypertensive or hypotensive urgency	4	0	4
Non-cardiac	10	2	12
Fall	2	0	2
Abnormal labs	1	0	1
Urinary obstruction	0	1	1
Planned procedure	0	1	1
Cancer	1	0	1
Dizziness, syncope	1	0	1
COVID, infection	1	0	1
GI issues	3	0	3
Difficulty swallowing	1	0	1

© 2025 Dodson JA et al. *JAMA Network Open*.

eFigure 1. Sample layout of mHealth-CR software (Moving Analytics platform)

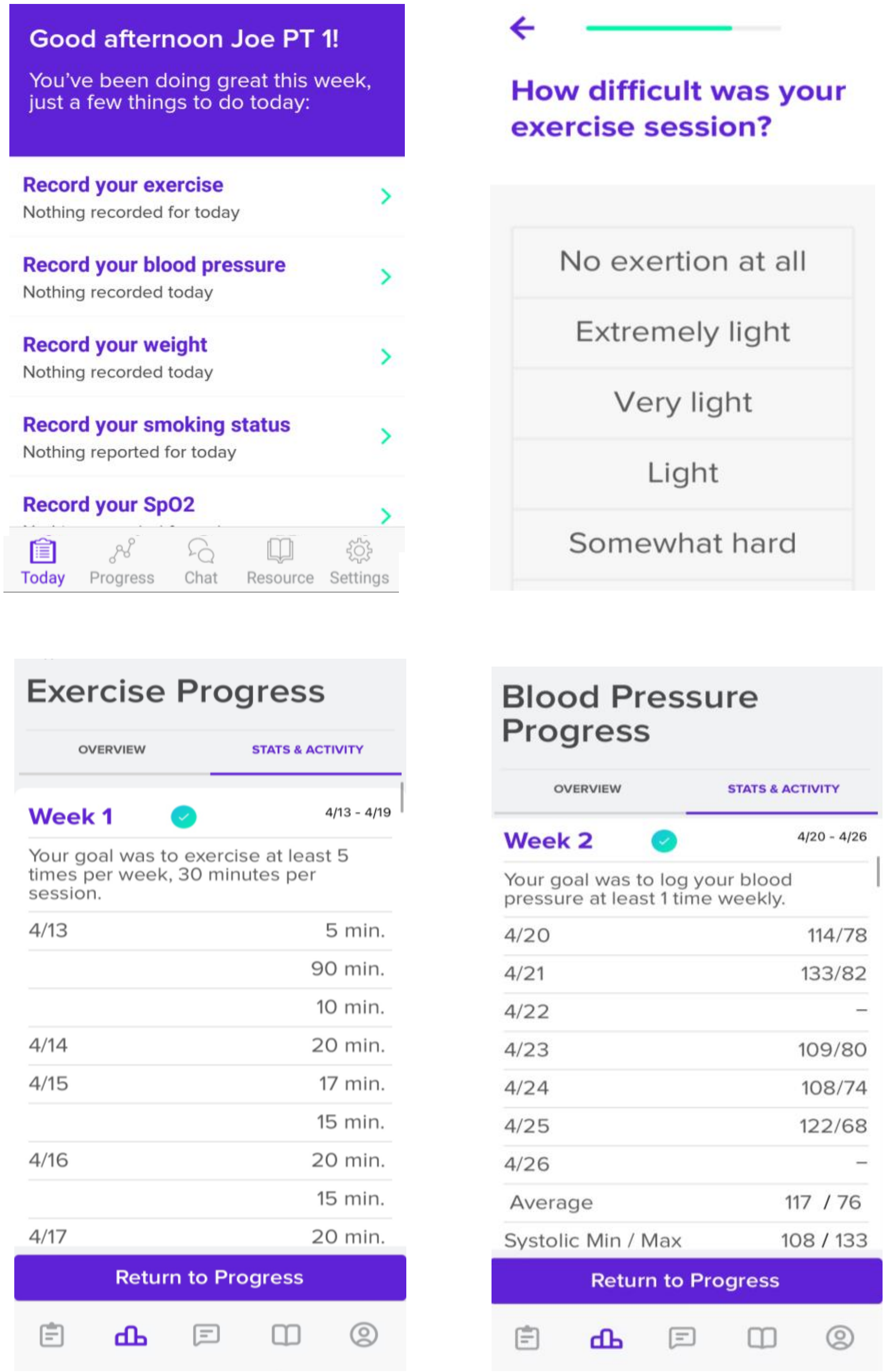

eFigure 1 demonstrates user interface for Moving Analytics software, which is designed for daily recording of exercise activity and rating of perceived exertion. For purposes of RESILIENT, the software was delivered on Android-capable device (Samsung Galaxy) with cellular functionality, paid for by the study, to obviate the need for a home WiFi network.

© 2025 Dodson JA et al. *JAMA Network Open*.

eFigure 2. Results of Bayesian analysis

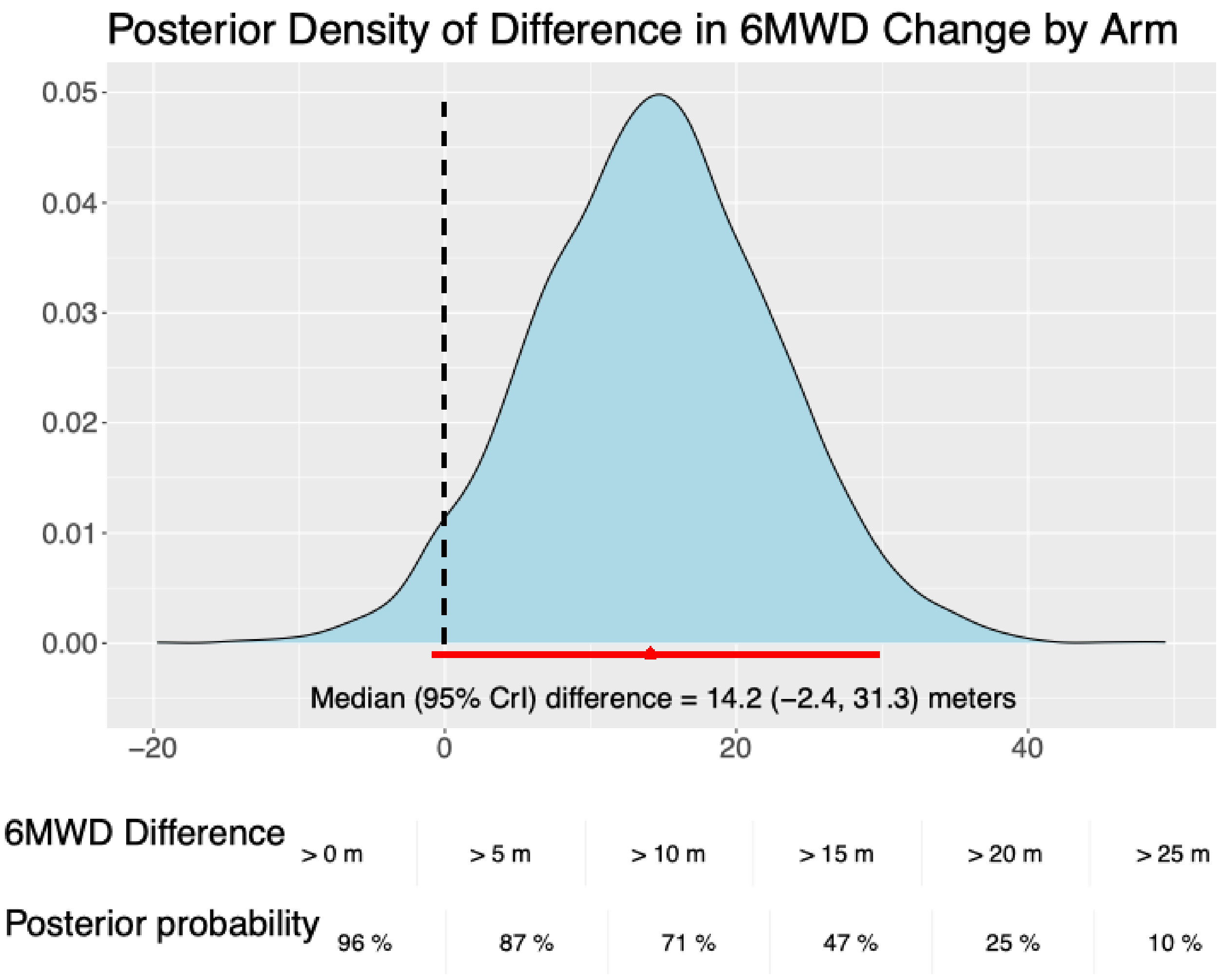

eFigure 2 shows an exploratory Bayesian analysis of the primary outcome (change in 6MWD). The posterior median and 95% credible interval of the 6MWD change was 14.2 [-2.4, 31.3] meters. A density plot of the full posterior region with 95% credible interval (in red) is shown in eFigure 2. The posterior probability that the change was >0 meters was 96%, >10 meters was 71%, >20 meters was 25%, and >25 meters was 10%. Therefore, based on the posterior probability, there is a high chance that the difference in improvement was greater than 0. However, the likelihood that the improvement is clinically significant (typically >25 meter change based on prior literature) is low.

© 2025 Dodson JA et al. *JAMA Network Open*.

eFigure 3. mHealth-CR engagement among intervention arm participants

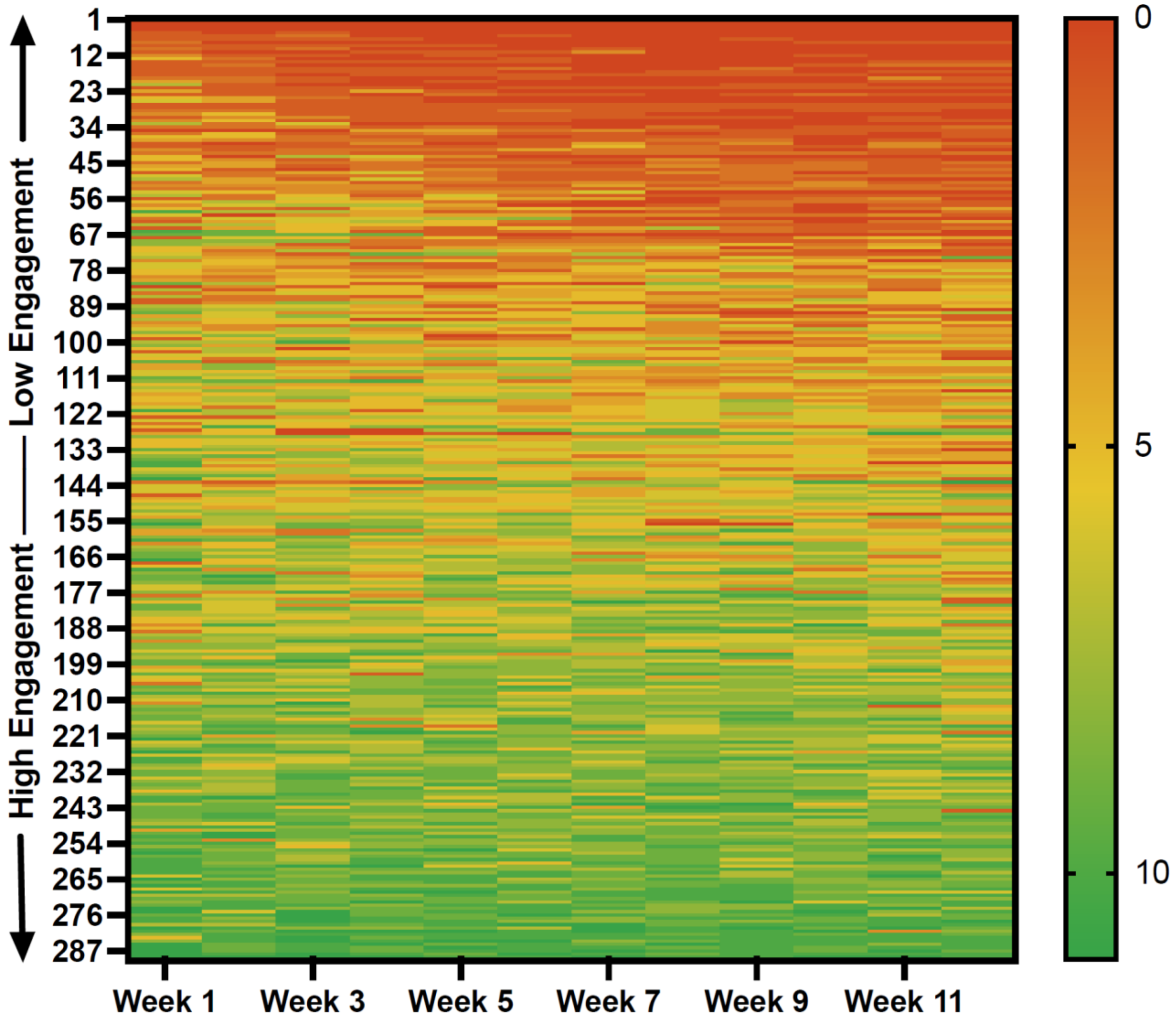

eFigure 3 shows mHealth-CR engagement patterns for the 298 participants in the intervention arm. Weekly engagement levels are a composite variable based on logging of daily exercise in the Moving Analytics Platform (score 1-7 based on number of days), completing a weekly phone call with the exercise therapist (8), sending a weekly electronic communication to the exercise therapist (9), viewing an assigned educational video weekly (10), and weekly measurement of blood pressure and logging (11). A score of 11 represents completion of all elements in a given week. The color spectrum represents high engagement (green) to low engagement (red), with study participants rank ordered by aggregate engagement score (lowest engagement at top of Figure).

© 2025 Dodson JA et al. *JAMA Network Open*.
