# Supplementary material for: Rehabilitation at Home Using Mobile Health for Older Adults Hospitalized for Ischemic Heart Disease: The RESILIENT Randomized Clinical Trial
Source: JAMA Netw Open. Author manuscript; Available in PMC 2025 Jul 11. (PMC12247035; doi:10.1001/jamanetworkopen.2024.53499)
Supplement: Supplement 3 — Data Sharing Statement [file NIHMS2086641-supplement-Supplement_3.pdf]

Data Sharing Statement

Dodson. Rehabilitation at Home Using Mobile Health for Older Adults Hospitalized for Ischemic Heart Disease. *JAMA Netw Open*. Published January 08, 2025.
doi:10.1001/jamanetworkopen.2024.53499

Data
Additional Information: NCT03978130
Data available: Yes
Data types: Deidentified participant data
How to access data: Data will be made available to other investigators upon reasonable request to the Principal Investigator, following final publication of the primary and secondary study results.
When available: beginning date: 01-01-2026

Supporting Documents
Document types: None

Additional Information
Who can access the data: Researchers whose proposed use of the data has been approved.
Types of analyses: For purpose of publishing exploratory findings within the trial, not reported elsewhere.
Mechanisms of data availability: With a signed data access agreement.
